# Femtosecond Laser Generation of LaCoO_3_ Perovskite Nanocatalysts for Preferential CO Oxidation

**DOI:** 10.1021/acsanm.5c03993

**Published:** 2025-10-30

**Authors:** Niusha Lasemi, Nevzat Yigit, Gerhard Liedl, Jürgen Fleig, Günther Rupprechter

**Affiliations:** † Institute of Materials Chemistry, TU Wien, Wien 1060, Austria; ‡ Institute of Production Engineering and Photonic Technologies, TU Wien, Wien 1060, Austria; § Institute of Chemical Technologies and Analytics, TU Wien, Wien 1060, Austria

**Keywords:** ultrafast laser ablation, LaCoO_3_ nanoperovskite, defect engineering, dislocations, twinning, CO-PROX

## Abstract

Green synthesis and defect engineering of LaCoO_3_ model nanocatalysts by femtosecond pulsed laser ablation in liquid (fs-PLAL) led to the formation of two types of nanoperovskites: stoichiometric LaCoO_3_ and nonstoichiometric cobalt-rich nanoparticles. Micro-Raman analysis revealed pronounced second-order phonon scattering, suggesting a high defect density. The defect spatial distribution was evaluated by high-resolution electron microscopy, employing Fourier filtering and image reconstruction. Increasing the laser fluence increases the surface defect density due to the fast cooling of primary nanoparticles, a process intensified by the inherently ultrashort pulses. Laser-produced nanoparticles exhibited internal defects, a characteristic absent in those produced by a chemical method. Chemically derived nanoparticles, originally perfectly crystalline, formed grain/twin boundaries during calcination when their irregular shapes coalesced. Compared to a chemically synthesized reference catalyst, nanoparticles laser-synthesized at 5.8 J cm^–2^ showed the highest CO conversion during PROX in excess H_2_ at 400 °C. Perovskite produced at 5.8 J cm^–2^ and 5.1 J cm^–2^ also showed higher CO_2_ selectivity (89% and 83%, respectively, versus 28% of the reference), as well as excellent stability at 350–400 °C.

## Graphical abstract

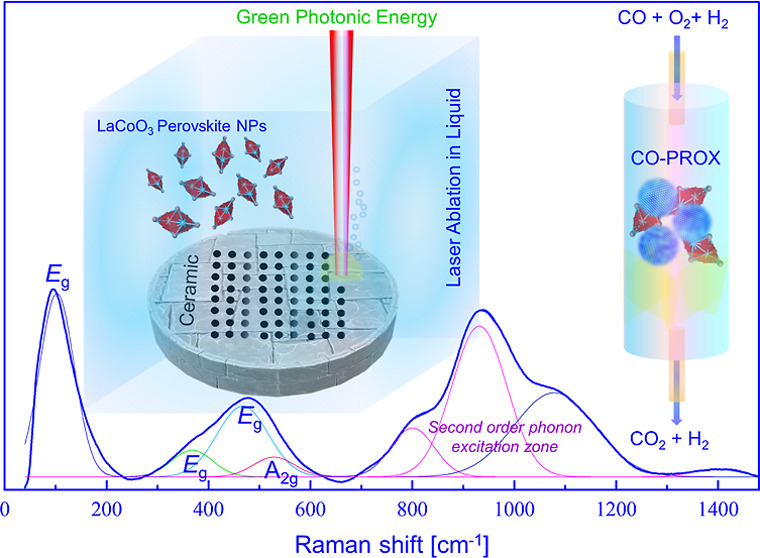

## 1 Introduction

Perovskite nanomaterials have shown favorable performance in heterogeneous catalysis as well as many other applications due to their unique structural and electronic properties.^1,2^ Specially, lanthanum cobalt oxide (LaCoO_3_) nanoperovskite has gained significant interest owing to its excellent catalytic activity in numerous reactions, including oxidation and reduction.^3–6^ In preferential CO oxidation (PROX), the challenge lies in selectively oxidizing CO without oxidizing H_2_ and promoting CO methanation. Usually, the choice of catalyst material plays a crucial role in PROX. Noble metals were frequently used for CO oxidation and have demonstrated high activity.^7,8^ However, this high activity also leads to oxidation of H_2_. Transition metal oxides, such as CuO,^9^ Co_3_O_4_ ^10^ and Pd/MgO^11^ were also used and offer better selectivity. Generally, active metals can cycle between different oxidation states in redox processes during CO oxidation. For example, in cobalt-based catalysts Co can exist in different oxidation states (e.g., Co^2+^ and Co^3+^) which may interconvert during the reaction.^10^ This facilitates redox cycles which is essential for catalytic activity in CO oxidation. Another advantage is the abundance of oxygen vacancies in LaCoO_3_, which aids in promoting CO oxidation by facilitating oxygen transfer for redox reaction cycles.^12,13^ Up to now, the focus in perovskite-based catalysis has often been on exsolution and oxygen vacancies, whereas little attention was paid to crystalline defects within the nanocatalysts. Therefore, further studies are required to reveal the role of specific defects in catalyst performance.

Clearly, the synthesis technique affects the formation and abundance of active sites and defects. Prior studies have demonstrated the benefits of pulsed laser ablation in liquid (PLAL) for nanoparticle (NP) generation.^14–25^ Laser-induced defect engineering may drive innovation in heterogeneous catalysis by offering a novel approach to material design. For example, defect-rich CuZn model catalysts, generated by femtosecond laser ablation, have shown potential in hydrogenation, with extensive pre- and postreaction electron microscopy revealing a high density of defect sites.^23^ During PLAL, the high plasma temperature induced by ultrashort pulses^26^ and rapid cooling and fast solidification of primary nanoparticles led to insufficient time to achieve complete crystalline ordering.^20,21,23,27^ According to theoretical studies, femtosecond laser pulses exhibit the fastest cooling rate (10^12^ K/s) among various pulse durations.^28^ Hence, defects and low-coordinated surface sites form intrinsically, without additional mechanical (e.g., ball milling) or physicochemical (e.g., laser post-processing) intervention.

The abundance, distribution, and nature of defect structures play a crucial role in determining catalytic performance.^21,23,29–33^ Grain boundaries and twin boundaries are common crystalline defects in PLAL samples. They may both improve the reaction kinetics by enhancing reactant adsorption at low coordinated sites and may facilitate reaction between species at neighboring sites. In every grain boundary, i.e. interface between grains, there is a region of structural disorder.^34^ This disorder manifests as higher density of defects including low-coordinated sites and oxygen vacancies in the catalyst. Experimental and theoretical studies of Cu­(O) catalyst in CO-PROX revealed that O_2_ dissociation is favored at low-coordinated sites along steps and grain boundaries.^35^ However, studies specifically probing the impact of defects in perovskite NPs on PROX activity are lacking.

To date, there exist few studies regarding the synthesis of perovskite structures by laser illumination, such as CeAlO_3_ produced with a nanosecond diode pumped laser,^36^ BaTiO_3_ prepared with a picosecond laser^37^ or an Ytterbium-doped Potassium Gadolinium Tungstate laser (Yb/KGW) with a duration of 1 ps or 170 fs,^38^ or synthesized with an Ytterbium-doped fiber laser (200 ns).^39^ However, the application of the PLAL method to the specific synthesis of LaCoO_3_ nanoperovskites in liquid remained unexplored, to the best of our knowledge. This presents an opportunity to investigate for the first time both the feasibility of laser-driven LaCoO_3_ nanoparticle formation and the potential catalytic properties of the resulting material.

Given the inherent difficulty to create defect-rich nanostructures by conventional chemical methods, herein femtosecond laser ablation in ethanol was utilized to synthesize defect-rich LaCoO_3_ nanoperovskites. The perovskite nanoparticles were applied as catalyst for preferential CO oxidation (PROX). Perovskite targets were characterized before and after laser ablation using optical microscopy, scanning electron microscopy (SEM), profilometry, X-ray diffraction (XRD), and confocal micro-Raman spectroscopy. Next, the generated perovskite (colloidal) nanoparticles were analyzed using UV/vis spectroscopy, dynamic light scattering (DLS), XRD, and micro-Raman spectroscopy. Transmission electron microscopy (TEM), high-resolution TEM (HRTEM), complemented by Fast Fourier Transform (FFT) filtering and Inverse Fast Fourier Transform (IFFT) image reconstruction, high-angle annular dark-field scanning TEM (HAADF-STEM), and energy-dispersive X-ray (EDX) mapping were used for comprehensive defect-structure analysis.

## 2 Materials and Methods

### 2.1 Perovskite Pellet Preparation

LaCoO_3_ perovskite was produced by the Pechini method using La_2_O_3_ and Co powders (99.99%, Sigma-Aldrich). The powders were stoichiometrically mixed and dissolved in nitric acid followed by using citric acid (TraceSELECT, 99.99%) for chelation. The resulting powder was calcined at 1000 °C and isostatically pressed at 150 MPa to pellets, and subsequently sintered in air at 1200 °C for 12 h to obtain LaCoO_3_ targets.

### 2.2 Polishing

The LaCoO_3_ targets (1 cm^2^) were polished in an ALLIED grinding device using a two-step process with SiC grinding papers of 1200 and 2400 grit. After polishing, the targets were cleaned ultrasonically (LAVAMIN).

### 2.3 Femtosecond Laser-Synthesis of Perovskite NPs

Figure 1 illustrates the experimental PLAL setup which is detailed in our prior work.^18^ The setup comprised a newly designed aluminum cell (15 mm in height) housing the LaCoO_3_ target screwed onto the cell bottom. The reactor was closed with a 1 mm thick round quartz window, sealed by Kalrez-*O*-rings and clamped between the cell body and an aluminum cap by four screws. Then, the PLAL reactor was mounted on a motorized XYZ stage, being oriented with its window perpendicular to the horizontal laser beam.

**Figure 1:**
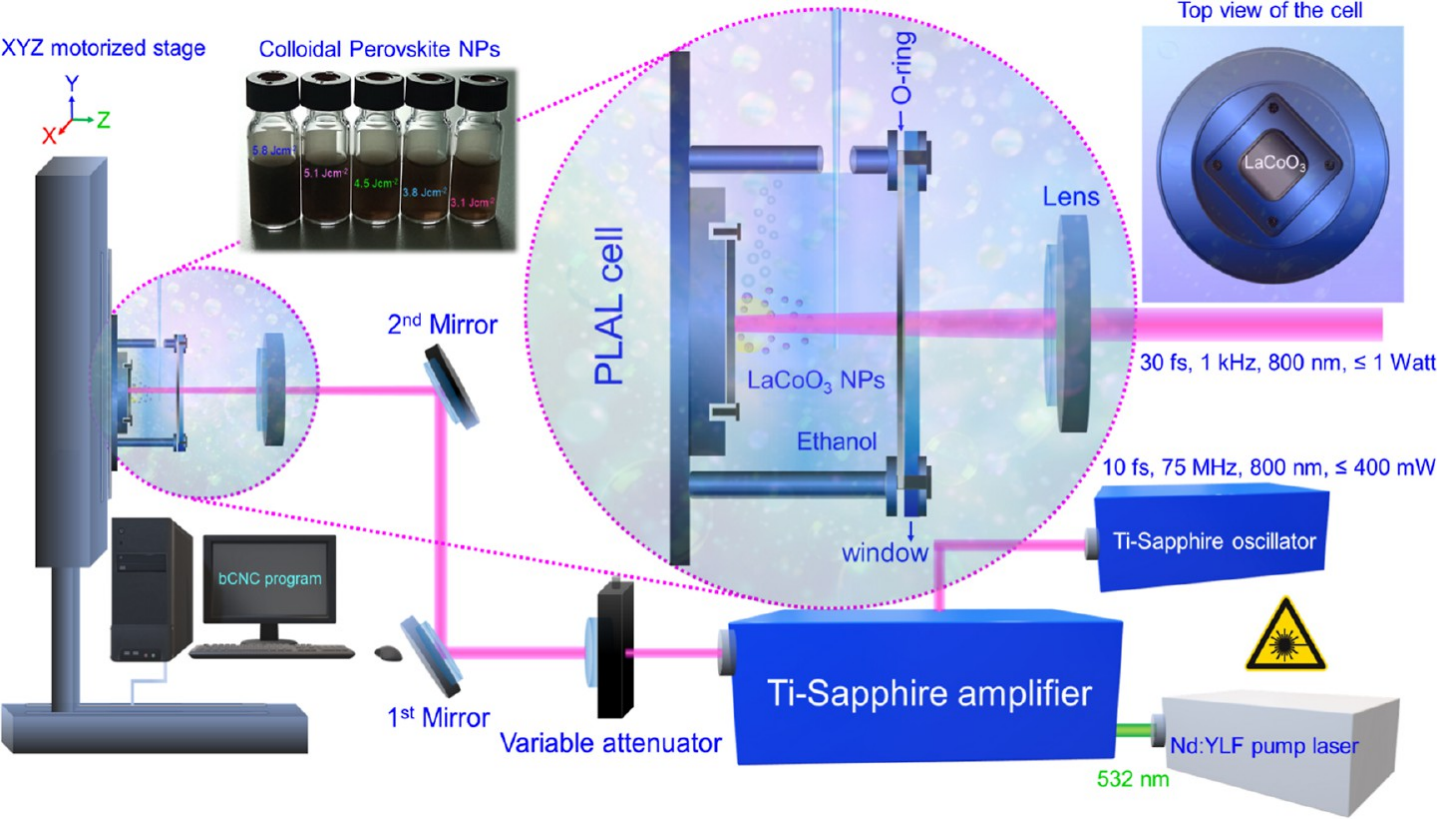
Experimental setup for femtosecond pulsed laser ablation in liquid (PLAL). The schematic illustrates the femtosecond laser system which comprises a Ti/sapphire oscillator, Ti/sapphire amplification unit, and a Nd/YLF pump source. The PLAL cell is situated on a motorized XYZ scanning stage. Magnified: The newly designed aluminum cell in side and top view, illustrating production of colloidal LaCoO_3_ perovskite nanoparticles. The photograph of vials displays nanoparticles fabricated in ethanol at different fluences.

The femtosecond laser system (FEMTOPOWER compact PRO) comprised a seed laser, a commercial unit from SPECTRA-PHYSICS (*P* ≤ 400 mW, 800 nm, 10 fs, 75 MHz) and a continuous-wave Nd/YLF pump laser (532 nm). Chirped pulse amplification (CPA) was employed by applying third-order dispersion precompensating mirrors to stretch the pulse prior to amplification. After directing the laser beam to the amplification stage (Ti-sapphire crystal) and pumping with the green laser, the resulting pulse underwent compression using a pair of prisms. This yielded a final output with characteristics of *P* ≤ 1 W, 30 fs, and 1 kHz.

The LaCoO_3_ target, mounted in the PLAL cell and submerged in ethanol, was ablated by sequences of 1000 laser shots (*N*_1000_) at an output power of 100 mW. To adjust the focal point of the laser beam on the target, a fused silica plano-convex lens (FS lens, EKSMA OPTICS) with a diameter of 25.4 mm and a focal length of 100 mm was utilized, with ablation occurring at different lens-to-target distances. For laser ablation, the femtosecond laser setup was combined with a computer-controlled motorized scanning stage. This automatically translated the PLAL cell, ensuring that fresh target areas were ablated after every 1000 pulses.

A variable attenuator, a combination of a beam splitter and a polarizer with a quartz half-wave plate, was used to adjust the output power, monitored by a power meter (OPHIR Photonics). Next, optical microscopy, coupled with Zeiss AxioVision software, was employed to evaluate the diameter of the ablated region on the perovskite target at different laser fluences (*F*). This evaluation (detailed in Figure S1) considered an average of 30 ablation craters. The calculated laser fluences, ranging from 3.1 to 5.8 J cm^–2^, are summarized in Table S1.

The Supporting Information (SI) provides a thorough description of all characterization techniques employed to analyze the targets and colloidal nanoparticles, including optical microscopy (Supporting Note 2.1), profilometry (Supporting Note 2.2), scanning electron microscopy (SEM) (Supporting Note 2.3), X-ray diffraction (XRD) (Supporting Note 2.4), dynamic light scattering (DLS) (Supporting Note 2.5), UV/vis spectroscopy (Supporting Note 2.6), confocal micro-Raman spectroscopy (Supporting Note 2.7), and transmission electron microscopy (Supporting Note 2.8).

### 2.4 Catalytic Evaluation under Flow Conditions

The catalytic reaction setup consisted of a continuous-flow fixed-bed quartz reactor under atmospheric pressure, coupled to an online mass spectrometer (OmniStar from Pfeiffer Vacuum) and a gas chromatograph (Agilent 6890) featuring a HP-PLOTQ column and a flame-ionization detector (FID) with a methanizer for continuous gas analysis.

The reactor was loaded with ∼600 μg of the perovskite catalyst deposited on inert quartz wool via drop casting. Prior to the reaction, perovskite catalysts were pretreated/cleaned by heating to 400 °C with a heating rate of 10 °C/min in 20 vol % O_2_ and 80 vol % Ar and held at this temperature for 1 h. Once the reactor had cooled to room temperature, CO-PROX was performed in a gas mixture of 1 vol % CO, 1 vol % O_2_, 50 vol % H_2_ and 48 vol % Ar (total flow of 50 mL min^–1^). The reactor was heated to 400 °C at a rate of 2 °C/min, with reactants and products monitored by MS and GC.

## 3 Results and Discussion

As shown below, the observed catalytic activity is promoted by low-coordinated surface sites stemming from defective crystalline structures, such as those generated through fs-PLAL. The laser fluence provides not only a means to tailor the nanoparticle size distribution but, importantly, also the abundance of defects. The following sections detail the femtosecond laser ablation of LaCoO_3_ perovskite and the comprehensive characterization of defect-rich perovskite nanoparticles. Chemically synthesized LaCoO_3_ as reference was also thoroughly studied, including various electron microscopy techniques to investigate the presence and type of defects. Then, the catalytic activity of PLAL-synthesized LaCoO_3_ NPs was explored for CO-PROX, focusing on conversion/selectivity and comparison with the chemically synthesized perovskite NPs. Lastly, postreaction analysis was performed on the best laser-synthesized catalyst to examine whether the initial defects persisted throughout the reaction.

### 3.1 Electron Microscopy of Perovskite NPs

Transmission electron microscopy was employed to investigate the size and morphology of LaCoO_3_ NPs produced at 5 different laser fluences *F*, ranging from 3.1 J cm^–2^ to 5.8 J cm^–2^. These fluences were selected to avoid unwanted nonlinear processes.^17^ HRTEM enabled observation of lattice fringes and defects. The FFT of HRTEM images provided local diffraction information to assess the crystalline or amorphous nature of nanoscale regions. Fourier filtering and IFFT were also applied to enhance the visualization of defects. Additionally, HAADF-STEM coupled with EDX elemental mapping yielded detailed information regarding both the chemical composition and elemental distribution within the perovskite structure.

BF-TEM in Figure 2a shows the shape and distribution of fs-produced LaCoO_3_ perovskite NPs at *F* 5.8 J cm^–2^ with four areas selected for high resolution evaluation. HRTEM in Figure 2a­(i) shows a nanoparticle containing multiple domains. The corresponding FFT pattern exhibits reflections from the (110), (104), and (024) planes which is consistent with a rhombohedral phase. A single twin boundary can be visualized by applying a spot mask on the FFT and performing an IFFT. The FFT pattern related to HRTEM 2a­(i) displays rings with bright spots, indicating a polycrystalline material.

**Figure 2:**
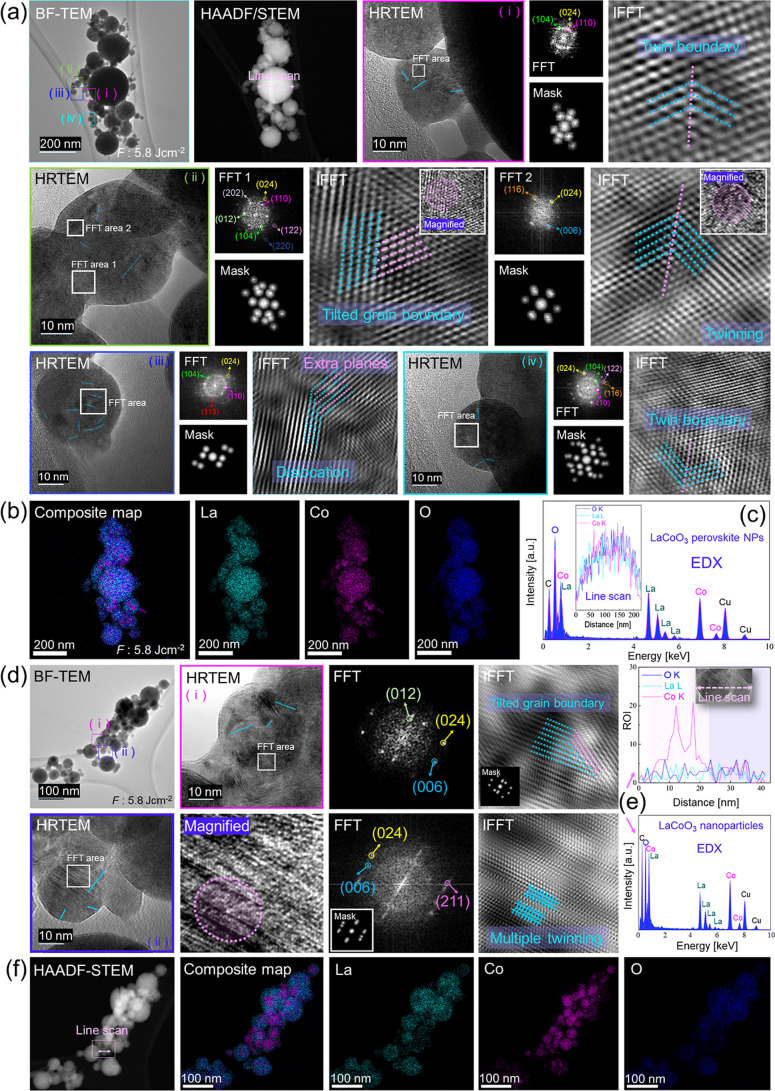
Electron microscopy and EDX mapping analysis of LaCoO_3_ perovskite NPs femtosecond laser-produced (*F*: 5.8 J cm^–2^) in ethanol. (a) First row: BF-TEM, HAADF-STEM, HRTEM (i) reveals a single twin boundary accompanied by its FTT, spot mask and IFFT. Second row: HRTEM (ii) showing single twining along with its FFT1 and corresponding mask/IFFT and FFT2 and corresponding mask/IFFT. A magnified zone is presented as inset. Third row: HRTEM (iii) and HRTEM (vi) with their respective analyzed FFT/spot mask and IFFT illustrating grain boundaries/dislocations and a single twin boundary, respectively. (b) Composite EDX mapping image displaying individual EDX elemental maps including La, Co and O. (c) EDX spectrum obtained from the BF-TEM area with a line scan spectrum shown as inset. (d) BF-TEM image of another area with marked regions i and ii. HRTEM (i), its FFT, IFFT and spot mask (inset) depict a single tilted grain boundary. HRTEM (ii), a magnified area, its FFT with a spot mask (inset) and its IFFT show multiple twining. (e) Line scan profile and EDX spectrum corresponding to BF-TEM image. (f) HAADF-STEM, a composite map with individual EDX elemental maps including La, Co and O.

Further analysis was applied to another nanoparticle, shown in the HRTEM image in [Figure 2a­(ii)]. The resulting FFT1 displays reflections from the (012), (110), (104), (202), (024), (122), and (220) planes. Applying a spot mask and IFFT revealed a tilted grain boundary, which is also clearly visible in the magnified inset. A tilted grain boundary represents an interface where two adjacent crystalline grains exhibit misorientation characterized by a specific tilt angle relative to a shared axis within their common boundary. FFT2 displays reflections from (006), (024), and (116), with the corresponding IFFT indicating a single twin boundary. HRTEM 2a­(iii) shows a nanoparticle with a region rich in grain boundaries. The FFT pattern displays reflections from the (110), (104), (024), (122) and (116) planes, and the corresponding IFFT image displays extra planes and dislocation defects which is visible in the magnified image (inset). HRTEM 2a­(iv) shows another defective perovskite nanoparticle. The FFT pattern displays reflections from the (110), (104), (113), and (024) planes, and the corresponding IFFT image reveals twinning.

Figure 2b shows a composite image of the EDX elemental mapping, illustrating the distribution of La, Co, and O in the sample. Specially for smaller NPs, the cobalt distribution in some areas is more concentrated, those areas are cobalt rich and mostly defective.

An EDX spectrum (Figure 2c) was obtained from the BF-TEM area and its corresponding atomic % contribution is given in Table S2 in the Supporting Information. Two different compositions were identified by EDX: stoichiometric LaCoO_3_ and a cobalt-rich, nonstoichiometric composition. For the first, the EDX line scan (inset in Figure 2c), which corresponds to the area marked in HAADF-STEM in Figure 2a, indicates a comparable amount of lanthanum and cobalt.

Figure 2d shows a BF-TEM image of another area, with two specified regions marked for detailed analysis. At first, HRTEM 2d­(i) imaging reveals a grain boundary defect. The corresponding FFT pattern displays reflections from the (012), (006), and (024) planes. Subsequent IFFT analysis confirms the presence of a tilted grain boundary. HRTEM image 2d­(ii), along with its magnified area, reveals multiple twinning. This observation is further shown and confirmed by the corresponding IFFT analysis. The FFT pattern displays reflections from the (006), (024), and (211) planes. Figure 2e shows the EDX spectrum acquired from the BF-TEM imaging area, and the corresponding atomic percentage contributions are given in Table S3.

The line scan profile from HRTEM 2d­(ii) clearly distinguishes between stoichiometric LaCoO_3_ and nonstoichiometric defective NPs. Figure 2f shows HAADF-STEM alongside a composite EDX elemental map, illustrating the distribution of La, Co, and O within the sample. EDX mapping reveals a Co-rich area.

To further demonstrate the general abundance of defects in fs-PLAL samples, additional analysis was applied to another collection of perovskite NPs (Figure S7). BF-TEM is marked with two areas for further analysis. HAADF-STEM also shows contrast difference due to cobalt rich areas. HRTEM S7­(i) shows a grain boundary-rich NP. Moiré fringes, originating from double diffraction, are also visible in HRTEM S7­(i) which should not be misinterpreted as defects. The FFT pattern shows reflections from the (110), (113), and (024) planes, and the corresponding IFFT pattern reveals a single twin boundary. Both the composite map and individual elemental maps show cobalt-rich zones.

HRTEM (ii) and IFFT in Figure S7c show twinning. While the presence of a Moiré pattern complicates interpretation, the area outside this pattern reveals a single twin boundary. (006) and (211) reflections were measured for the FFT. Figure S7d shows the EDX spectrum obtained from the BF-TEM imaging area, with the corresponding atomic % contributions presented as inset. Descriptions regarding analysis of further areas of the perovskite NPs femtosecond laser-produced at 5.8 J cm^–2^ can be found in the Supporting Information (detailed in Figures S8–S10).

To examine the effect of laser *F* variation, the laser fluence was decreased to 5.1 J cm^–2^. Figure 3a presents a BF-TEM image of the produced perovskite NPs, indicating two selected areas. HRTEM (i) and two corresponding FFTs are shown. The magnified area and IFFT analysis show extra planes resulting in atomic dislocation. The complexity of the defect area is reflected in FFT1, which displays (110), (104), and (204) planes, indicative of a pseudorhombohedral phase. Pseudorhombohedral structures, deviating slightly from ideal rhombohedral symmetry, indicate the presence of local deviations or distortions within some nanoparticles (e.g., dislocations). FFT2 displays (110) and (104) reflections and multiple twinning is evident from the IFFT2 image. HRTEM image (ii) and its corresponding IFFT image show a tilted grain boundary. The FFT pattern of this region displays (110) and (104) reflections.

**Figure 3:**
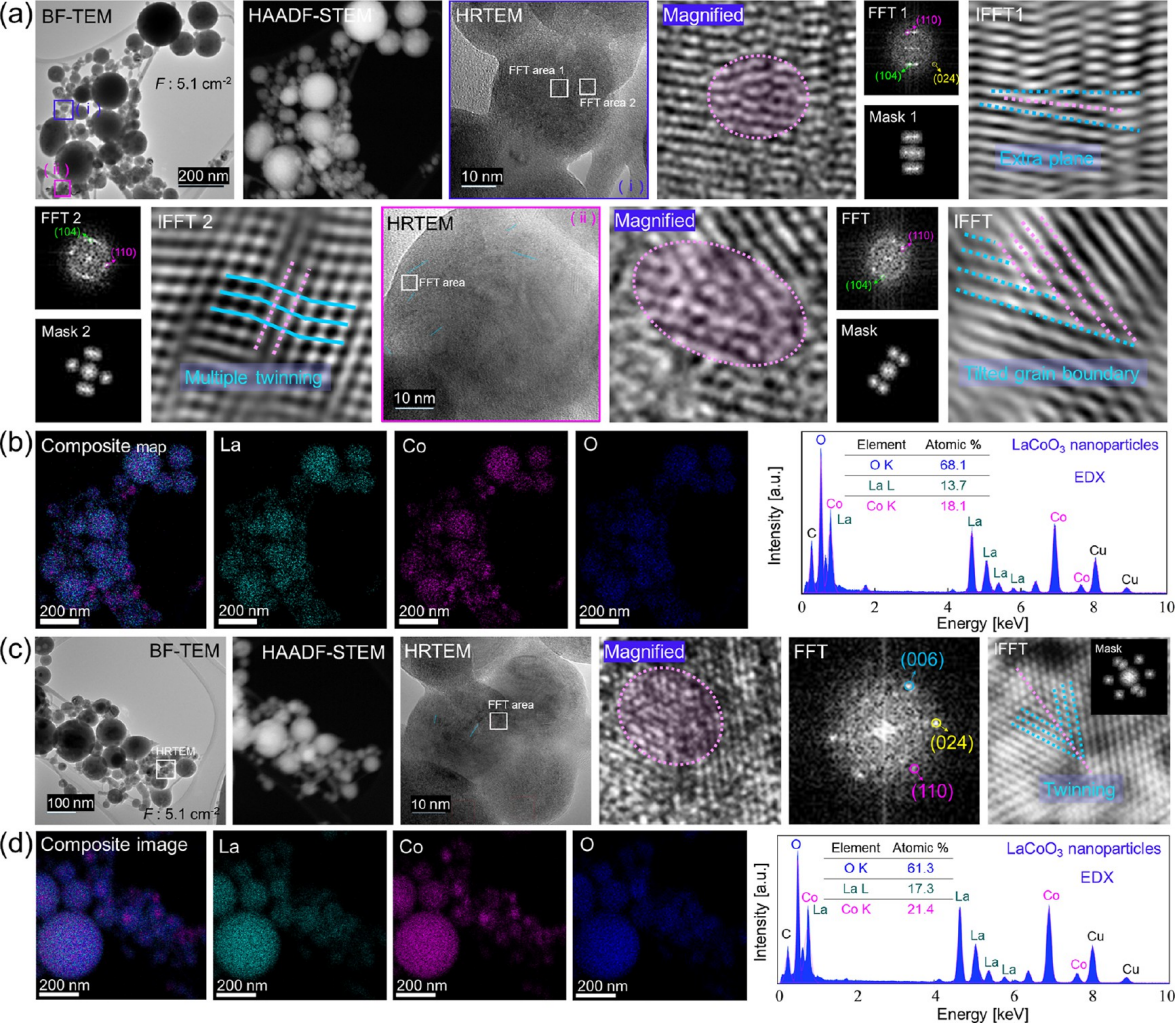
Electron microscopy and EDX mapping analysis of LaCoO_3_ perovskite nanoparticles femtosecond laser-produced (*F*: 5.1 J cm^–2^) in ethanol. (a) BF-TEM, HAADF-STEM, HRTEM (i) showing two marked FFT regions, a magnified zone related to FFT1, FFT1 with its corresponding mask/IFFT. Second row: FFT2 and its corresponding mask/IFFT. IFFT1 and IFFT2 show dislocation and multiple twining, respectively. HRTEM (ii), a magnified area, its FFT, mask and IFFT show a titled grain boundary. (b) Composite EDX mapping image indicating individual EDX elemental maps for La, Co and O along with an EDX spectrum acquired from the BF-TEM area. (c) BF-TEM image of another region, HAADF-STEM, HRTEM, and FFT with its IFFT and spot mask (inset) represent twinning. (d) A composite EDX mapping image with it corresponding individual EDX elemental maps for La, Co and O along with an EDX spectrum.

Figure 3b shows a composite image, individual elemental maps and an EDX spectrum. Similar to the perovskite NPs produced at 5.8 J cm^–2^, the smaller NPs exhibit cobalt-rich composition. Figure 3c shows BF-TEM of another area, as well as HAADF-STEM and HRTEM images. FFT displays reflections from the (110), (006), and (024) planes, and the corresponding IFFT image reveals twinning. The composite map (Figure 3d) shows a homogeneous distribution of La, Co, and O for larger NPs, while smaller NPs contain a higher concentration of Co. Line scan from the larger NP and smaller NP is shown in Figure S11, larger NPs are stoichiometric LaCoO_3_, whereas smaller NPs are Co-rich.

Figure S12a displays a BF-TEM image from another collection of perovskite NPs produced at a laser fluence of 5.1 J cm^–2^. The HRTEM image, with a corresponding FFT and mask as an inset, and the resulting IFFT image, reveal a single twin boundary. The FFT pattern displays reflections from (012), (110), and (024) planes. HAADF-STEM (Figure S12b), composite map (Figure S12c) and EDX spectrum (Figure S12d) show uniform contribution of La, Co and O. Further NP analysis and details are provided in the Supporting Information, including Figure S13 (additional electron microscopy/defect analysis, *F* = 5.1 J cm^–2^), Figure S14 (additional electron microscopy/defect analysis, *F* = 5.1 J cm^–2^) and Figure S15 (extra EDX analysis, *F* = 5.1 J cm^–2^).

The laser *F* was further decreased to 4.5 J cm^–2^ and BF-TEM shown in Figure 4a reveals a distribution of spherical perovskite NPs, consistent with observations at other laser fluences. HRTEM image 4a­(i) and its IFFT image show a single twin boundary. The FFT pattern (inset) exhibits reflections from the (006) and (024) planes. The HRTEM image 4b­(ii) (Figure 4b) displays two selected areas for FFT analysis. FFT1 exhibits reflections corresponding to the (012), (110), (024), (116), and (214) planes, while FFT2 shows diffraction spot for the (110) and (104) planes. Both IFFT images correspond to a twin boundary. Composite map (Figure 4c) shows multiple Co-rich areas.

**Figure 4:**
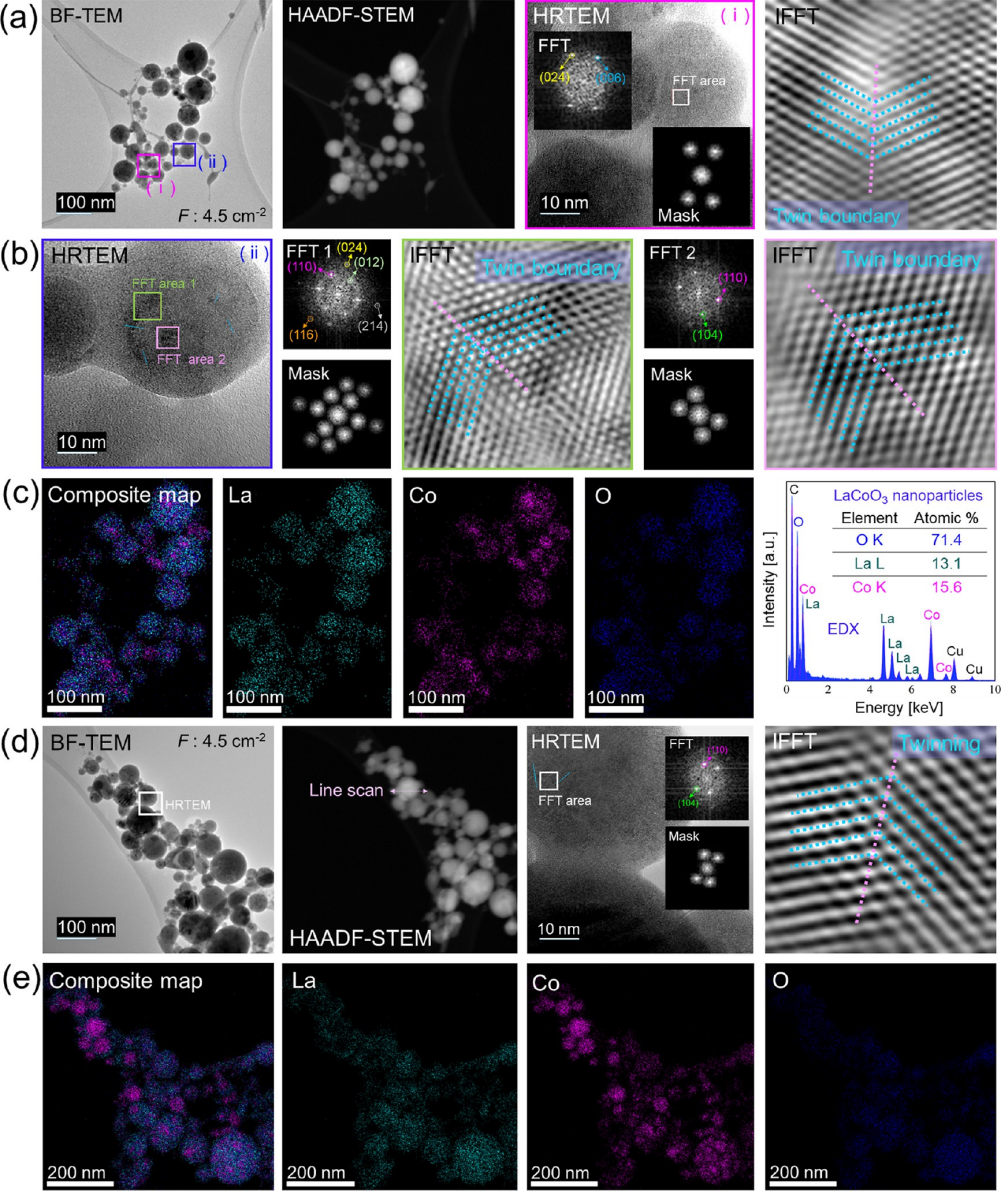
Electron microscopy and EDX mapping analysis of LaCoO_3_ perovskite nanoparticles femtosecond laser-produced (*F*: 4.5 J cm^–2^) in ethanol. (a) BF-TEM, HAADF-STEM, HRTEM (i) with its FFT (inset) and IFFT reveal a single twinning. (b) HRTEM (ii) showing two marked FFT regions, FFT1 and corresponding mask/IFFT and FFT2 and corresponding mask/IFFT also display a single twin boundary. (c) Composite EDX mapping image indicating individual EDX elemental maps including La, Co and O with an EDX spectrum obtained from the BF-TEM area. (d) BF-TEM image of another area, HAADF-STEM, HRTEM with FFT and mask as insets and its IFFT represent twinning. (e) A composite EDX mapping image with its corresponding individual EDX elemental maps for La, Co and O.

BF-TEM, HAADF-STEM and HRTEM images of another area of NP distribution are shown in Figure 4d. The FFT pattern (inset) displays reflections related to (110) and (104) planes. IFFT image reveals a single twin boundary defect. Composite map (Figure 4e) also shows a combination of cobalt-rich areas and stochiometric perovskite NPs. This was further analyzed acquiring an EDX spectrum and EDX line scan, as shown in Figure S16. The elemental composition confirms the presence of a uniform stoichiometric perovskite and areas with Cobalt enrichment. Further analysis of perovskite NPs produced at 4.5 J cm^–2^ is described in the Supporting Information including Figure S17 (additional EDX analysis), Figure S18 (further EDX analysis) and Figure S19 (extra EDX analysis).

Further reduction of the laser fluence aimed at exploring the presence of defects, nanoparticle morphology and size. Twin boundaries were also observed in LaCoO_3_ produced at lower fluences, consistent with previous applied laser fluences. However, due to the infrequent occurrence of defective structures, it was challenging to characterize them. As expected, the number of defects somewhat decreased. The Supporting Information which detail the perovskite nanoparticles synthesized at 3.8 J cm^–2^ includes Figures S20 (additional electron microscopy/defect analysis), Figure S21 (further EDX line scans), Figure S22 (further electron microscopy/defect analysis); Figure S23 (extra EDX mapping analysis) and Figure S24 (supplementary EDX mapping, Co-rich perovskite). Details on the perovskite nanoparticles synthesized at 3.1 J cm^–2^ are provided in the Supporting Information, including, Figure S25 (additional electron microscopy/defect analysis), Figure S26 (extra electron microscopy/defect analysis), Figure S27 (further EDX mapping), Figure S28 (further EDX analysis) and Figure S29 (supplementary EDX analysis of another NP distribution).

A quantitative analysis of the defect density may reveal which catalysts may possess more active sites and should then perform better in catalysis. Following detailed HRTEM/FFT/IFFT analysis of all PLAL-synthesized perovskite samples, further calculations were done to quantify the surface defect densities (ρ_surface defect_) for spherical NPs. This can be calculated by the number of surface defects–obtained from HRTEM images - divided by the surface area of the observed NPs (assuming spherical shape). To simplify the analysis, different defect types were not differentiated. Instead, the defect density was based on all observed defects such as twin boundaries, grain boundaries, and dislocations. This approach provides a concise accounting of the structural imperfections. Table 1 shows the calculated defect densities of LaCoO_3_ produced at various laser *F*. Full details regarding the calculation of the surface defect density are provided in Section 4.2 of the Supporting Information.

**Table 1:** Calculated Surface Defect Densities of LaCoO_3_ Perovskite NPs Femtosecond Laser Produced at Various *F*

*F* [J cm^–2^]	ρ_surface defect_ [nm^–2^]
5.8	6.5 × 10^–4^
5.1	4.8 × 10^–4^
4.5	4.4 × 10^–4^
3.8	3.0 × 10^–4^
3.1	2.8 × 10^–4^

As shown in Table 1, the surface defect density increases as the laser *F* is increased. When nanoparticles were produced with the highest *F* of 5.8 J cm^–2^, defects were more abundant, as observed by HRTEM. Given that femtosecond pulses are considerably shorter than the electron–lattice relaxation time (10^–10^ to 10^–12^ s),^40,41^ the rapid cooling of the hot plasma plum and subsequent fast solidification of nanoparticles inherently promotes defect formation.^20,23^ The plasma plume temperature also increases with increasing laser fluence, leading to an even more abrupt cooling and fast solidification of the nanoparticles, conditions that intrinsically enhance defect formation.

A quantitative analysis of the Co/La elemental ratio obtained at varying laser *F* ranging from 5.8 to 3.1 J cm^–2^ was derived from averaging all EDX data, and yielded the following values: 14:16, 18:15, 16:14, 18:12, 14:13. The cobalt contribution was mostly slightly higher. The Co/La atomic % ratios, obtained via EDX at various laser fluences, are summarized in Table S4.

### 3.2 Spectroscopic and Diffraction Analysis of Perovskite NPs

Micro-Raman spectroscopy, XRD, UV/vis, and Dynamic Light Scattering (DLS) analyses of perovskite nanoparticles produced at various fluences are shown in Figure 5, revealing their chemical composition, crystallinity, optical properties, and hydrodynamic characteristics, correspondingly.

**Figure 5:**
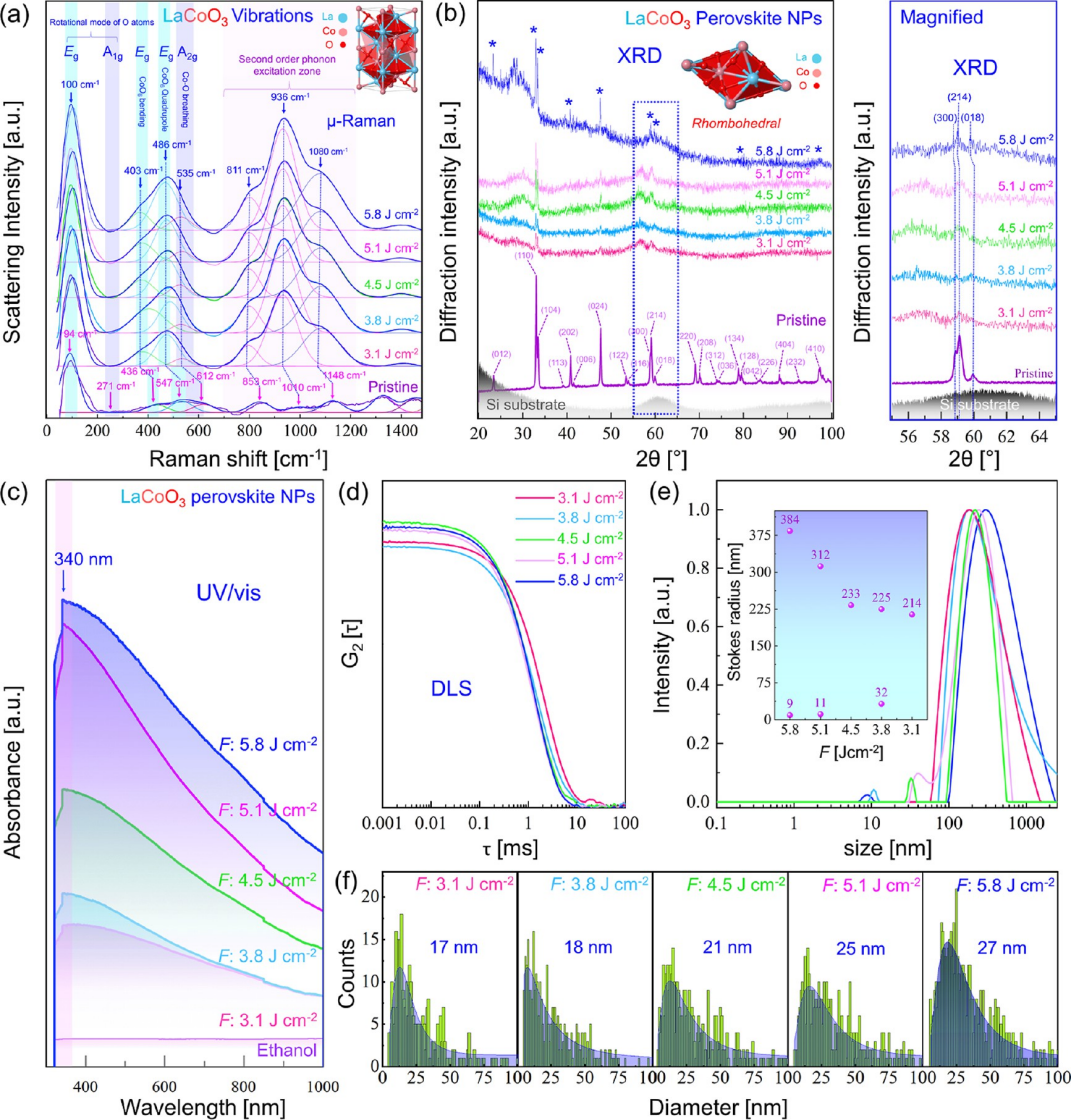
Spectroscopic and diffraction characterization of pristine LaCoO_3_ target and LaCoO_3_ perovskite nanoparticles femtosecond laser-produced at various *F*. (a) Confocal micro-Raman spectra; a Gauss fit was performed on the experimental data, yielding cumulative fits and corresponding deconvoluted peaks, optimized based on the best *R* ^2^-value (*R* ^2^ > 0.95). (b) X-ray diffraction patterns of the pristine target and perovskite NPs deposited on a silicon substrate, including a magnified view of the region of interest. (c) UV/vis spectroscopy of colloidal perovskite NPs. (d) Autocorrelation function, (e) Intensity-weighted hydrodynamic radius distribution for colloidal LaCoO_3_ nanoparticles; inset shows Stokes radius distribution for various applied *F*. (f) Number-weighted size distribution of perovskite NPs produced at various *F* evaluated by TEM imaging (LogNormal Fit).

The rhombohedral crystal structure is a distorted cubic system. In a perfect cubic perovskite structure, many vibrational modes are Raman inactive due to symmetry rules. However, when the cubic structure is distorted, the lowering of symmetry allows previously Raman-inactive phonon modes to become Raman active (Figure 5a). The *A*_1g_ + 4*E*_g_ Raman active modes are characteristic of rhombohedral perovskite structures, while 2*A*_1u_ + 3*A*_2g_ modes are silent.^40^

A very weak *A*_*1g*_ mode (rotational mode of O atoms around the *c* axis) was detected at ∼271 cm^–1^ only for the pristine target. The *E*_g_ mode (rotational mode of O atoms around the *a* and *b* axes) was detected for the target and nanoperovskites at ∼94 and ∼100 cm^–1^, correspondingly.

The *E*_g_ bending and *E*_g_ quadrupole modes were detected at ∼436 and ∼547 cm^–1^ for the pristine target, respectively. The quadrupole mode, mirroring the Jahn–Teller distortion atomic movements, is Raman active and involves either elongation or compression of the axial Co–O bonds.^42^ For nanoperovskite samples, these modes are red-shifted to lower frequencies, appearing at ∼403 and ∼486 cm^–1^. The redshift in nano LaCoO_3_ is due to phonon confinement, leading to changes in vibrational frequencies as compared to the bulk-like LaCoO_3_ target. In agreement with our finding, a redshift (ranging from 12 to 34 cm^–1^) was observed in the transvers and longitudinal optical phonon modes of laser synthesized SiC nanowires, which was attributed to the interplay of phonon confinement and internal stress.^43^

The *A*_2g_ breathing mode (inactive mode) was detected at ∼612 cm^–1^ for the pristine target and at ∼535 cm^–1^ for nanoparticles due to electron–phonon interactions.^44^ Forbidden (silent) *A*_2g_ ^42^ modes can also appear because of local distortion or deviation from the ideal rhombohedral structure (often described as pseudorhombohedral). These deviations can arise from impurities, strain, or defects (e.g., dislocations, twins, and grain boundaries) and their effect can be more pronounced in nanoparticles (potentially evident in second-order peaks). Second-order phonon excitations in the nanoperovskite were observed at ∼811, ∼936 and ∼1080 cm^–1^ with a higher scattering intensity compared to the first-order excitations. In contrast, the pristine target exhibited second-order excitations at ∼853, ∼1010 and ∼1148 cm^–1^ with intensities nearly comparable to the first-order peaks. This enhancement arises from a resonant condition where incident/scattered photons match electronic transitions in the material. Given the band gap values for nano LaCoO_3_ around ∼2.58 eV as reported in literature,^45^ a 532 nm laser (∼2.33 eV) is close to or within the range of these electronic transitions, suggesting the possibility of near-resonant Raman scattering, which leads to enhanced second-order scattering peaks. Furthermore, the intensity of both first- and second-order scattering peaks increased for samples produced at higher fluences, which may be related to the increase in defect abundance in NPs, in agreement with our electron microscopy analysis. The same behavior was observed in MoS_2_ containing strain and defects produced by chemical vapor deposition, exhibiting stronger second-order scattering.^46^

The XRD patterns of both pristine LaCoO_3_ target and nanoparticles synthesized at various *F* (deposited on a Si substrate) is shown in Figure 5b. Intense reflections were detected for the pristine sample consistent with a rhombohedral LaCoO_3_ phase (ICDD PDF No: 04-006-2093). Since the NP sizes are much smaller than the grain size of the target, the characteristic peak broadening in the NPs is attributed to the inverse relationship between crystallite size and peak width. Moreover, the magnified panel shows that reflections from the (300), (214), and (018) planes of the perovskite produced at 5.8 J cm^–2^ were slightly shifted to higher 2θ angles compared to those from samples produced at other *F* and the pristine target material. This indicates a decrease in lattice spacing, due to lattice strain or abundance of defects at the highest *F*, as previously observed by HRTEM/IFFT.

Figure 5c displays UV/vis absorbance spectra of LaCoO_3_ fs-laser produced in ethanol at various *F*. The colloidal nano perovskite exhibits strong absorbance peaks across the 300–800 nm wavelength range consistent with literature,^47^ with a maximum at ∼340 nm. The intense absorption peak at 340 nm is due to a ligand to metal charge transfer (LMCT) transition. Since LMCT typically occurs in the UV region, the peak observed at 340 nm in LaCoO_3_, is due to an electronic transition where an electron from the filled oxygen 2p orbitals is excited to the partially filled cobalt 3d orbitals upon UV light absorption. Notably, the absorbance intensity at 340 nm was highest for the nano perovskite synthesized at the highest *F*. This increased absorbance can be attributed to a higher ablation rate of nanoparticles, which correlates with increasing fluence.

DLS was used to determine the hydrodynamic diameter of LaCoO_3_ perovskite nanoparticles produced at laser *F* ranging from 3.1 to 5.8 J cm^–2^. The near unity signal-to-noise ratio (Figure 5d) observed in samples produced at 4.5, 5.1, and 5.8 J cm^–2^ correlates with a higher ablation rate or the presence of larger nanoparticles, which is expected at these elevated laser fluences. While DLS is valuable for determining the average hydrodynamic size of colloidal NPs, it often overestimates actual dimensions, as observed in prior studies of femtosecond laser-synthesized NiAu,^48^ Si^20^ and CuZn^23^ NPs. In this study, the Stokes radius of perovskite NPs increased with *F*. DLS measurements revealed larger mean sizes at higher *F* (Figure 5e). Although higher *F* yielded better nanoparticle productivity, it resulted in a broader size distribution. This size overestimation by DLS is likely due to NP agglomeration/aggregation and the solvation shell, both of which artificially increase the hydrodynamic diameter. Despite this limitation, DLS still proved capable of reflecting the overall trend in nanoparticle size as a function of *F*.

TEM size distribution analysis (Figure 5f), similar to DLS, shows a trend of increasing nanoparticle size upon increasing the applied *F*. A positively skewed log–normal distribution was observed for all size distributions. The width of the size distribution also increased with increasing *F*, shifting from monodispersity toward polydispersity. Higher *F* led to higher laser-induced plasma temperature, which increased thermal energy driving more vigorous Brownian motion,^49^ resulting in a higher frequency of nanoparticle collisions and thus larger final particle sizes. Furthermore, a comparative analysis was conducted to assess whether small or large NPs may play a more significant role in catalytic performance (which is apparently a surface process). This involved calculating their individual contributions to the overall surface area and volume of the NPs. As illustrated in Figure S30, for NPs produced at 3.1 J cm^–2^, the surface area contribution for smaller (<50 nm) NPs is 54%, while larger (>50 nm) NPs contribute 46%. The surface area ratios for LaCoO_3_ NPs produced at fluences of 3.8, 4.5, 5.1, and 5.8 J cm^–2^ are 52:48, 41:59 and 39:61% correspondingly. Surface-weighted and volume weighted data for LaCoO_3_ perovskite NPs synthesized at various *F* are compiled in SI including Table S5 (*F*: 3.1 J cm^–2^), Table S6 (*F*: 3.8 J cm^–2^), Table S7 (*F*: 4.5 J cm^–2^), Table S8 (*F*: 5.1 J cm^–2^) and Table S9 (*F*: 5.8 J cm^–2^).

### 3.3 Catalytic Testing in Continuous Flow CO-PROX

Three representative perovskite catalysts, synthesized at laser fluences of 4.5, 5.1, and 5.8 J cm^–2^, were chosen to evaluate their catalytic activity in the CO-PROX reaction. These fluences should highlight the impact of varying synthesis conditions on catalytic performance, as they offer a higher defect abundance. To ensure that merely the interaction of the PLAL catalyst with the gaseous reactants was studied (without any contribution from, e.g., an active oxide support), just inert quartz wool was used as support. The inert nature of the quartz wool was confirmed by MS analysis, as presented in Figure S31.

Comparing the catalytic activity of PLAL-synthesized NPs with that of chemically synthesized samples reported in literature may be ambiguous. Published studies often incorporate more active supports (e.g., alumina or ceria), doping, or employ higher loadings of metal nanocatalysts. Accordingly, for a more straightforward comparison, a chemically synthesized perovskite catalysts^6^ was examined herein, also just placed between quartz wool plugs. Accordingly, the activity and selectivity, determined by GC and MS, of the most active PLAL catalyst was compared to the chemically synthesized catalyst. While both catalysts exhibited the desired rhombohedral crystal structure, a morphological difference was observed: PLAL-synthesized NPs were predominantly spherical, whereas chemically synthesized NPs were irregular, multifaceted polygons (details in Section 3.4). The shape difference may also affect the catalytic performance.

The CO conversion of laser-produced LaCoO_3_ and chemically synthesized LaCoO_3_ is displayed in Figure 6a. Perovskite LaCoO_3_ nanoparticles produced at a fluence of 5.8 J cm^–2^ exhibited the highest catalytic activity among all laser produced samples. Its activity started at ∼175 °C, whereas the perovskite catalyst synthesized at 5.1 J cm^–2^ exhibited initial activity at 200 °C. Moderate activity at 300 °C was observed for perovskite produced at 4.5 J cm^–2^. Altogether, at the highest temperature the activity of perovskite produced at 5.8 J cm^–2^ was 2 and 4 times higher than that of perovskite produced at 5.1 and 4.5 J cm^–2^, respectively. Accordingly, this can be well correlated with the defect abundance in the nanostructures. Higher *F* leads to higher defect abundance, in line with previous work on femtosecond laser-synthesized ultrafine-grained Si^20^ NPs and defect-rich CuZn^23^ NPs used for ethylene hydrogenation.

**Figure 6:**
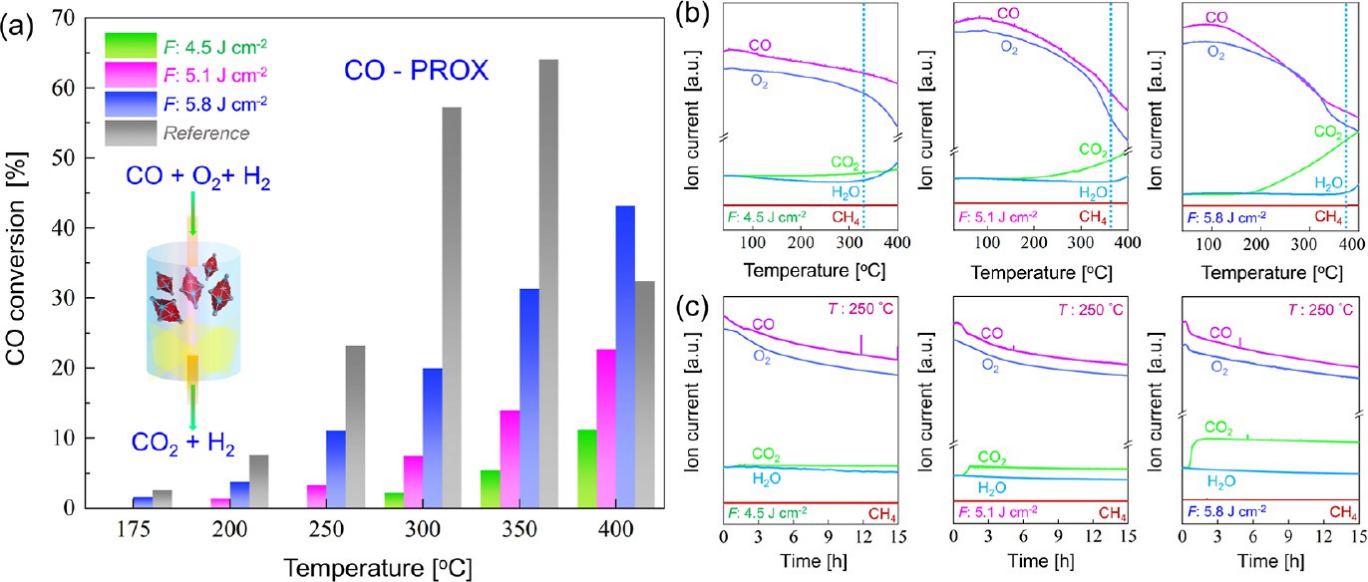
GC and MS analysis of CO-PROX on LaCoO_3_ perovskite NPs produced at femtosecond laser fluences of 4.5, 5.1, and 5.8 J cm^–2^ and of a chemically synthesized reference catalyst, deposited on inert quartz wool. (a) Comparison: CO conversion from GC as a function of temperature. (b) MS shows CO_2_ evolution increases for perovskite NPs femtosecond produced at 4.5 up to 5.8 J cm^–2^. Dashed lines indicate the onset of water formation. (c) MS confirms catalysts stability at 250 °C, over a period of 15 h.

As shown in Figure S32, CO_2_ selectivity was calculated based on the MS intensity of CO_2_ and H_2_O, with the signals normalized by the sensitivity factor (1.05 for CO_2_ and 1.42 for H_2_O), followed by background subtraction. The perovskite nanocatalyst laser-produced at *F* of 4.5 J cm^–2^ achieved 100% selectivity below 300 °C (Figures 6b and S32a). Similarly, catalysts prepared at 5.1 and 5.8 cm^–2^ showed 100% selectivity toward CO_2_ and complete CO conversion below 370 °C (Figures 6b and S32b,c). No methane formation was detected. To assess the stability of the three LaCoO_3_ perovskite nanocatalysts, PROX was performed at 250 °C for 15 h as shown in Figure 6c. No deactivation was detected under these conditions, indicating high stability.

For the reference sample, initial activity also started at ∼175 °C similar to LaCoO_3_ nanoparticles produced at a fluence of 5.8 J cm^–2^ and it showed CO conversion of 64% at ∼350 °C, which was almost 2 times higher than PLAL-NPs (5.8 J cm^–2^) at this temperature. However, at 400 °C, PLAL-NPs showed ∼1.5 times higher CO conversion than the reference sample.

MS data for the chemically synthesized reference catalyst are shown in Figure S33, along with the TEM-based size distribution. CO_2_ formation increased up to 350 °C and then decreased due to more H_2_O formation and occurrence of reverse water–gas shift reaction (RWGS: CO_2_ + H_2_ ⇄ CO + H_2_O).^50^ Thus, CO again increased at higher temperature (Figure S33a). In contrast, the laser-synthesized catalysts (Figure 6b) showed an increasing trend in CO_2_ formation upon increasing the temperature.

Table 2 presents the specific activity per gram of catalyst calculated at different temperatures for both femtosecond laser-produced LaCoO_3_ and the reference. The specific activity, expressed as moles of CO converted per second per gram of catalyst, generally increases with temperature for the laser-produced samples. However, for the reference catalyst, it showed an increase up to 350 °C, followed by a significant decrease due to occurrence of RWGS.

**Table 2:** Summarized Specific Activities at Different Temperatures for CO-PROX Reaction on LaCoO_3_ Perovskite NPs Femtosecond Laser-Produced at Fluences of 4.5, 5.1, and 5.8 J cm^–2^, and a Reference Catalyst

*T* [°C]	[mol s^–1^ g^–1^] 4.5 J cm^–2^	[mol s^–1^ g^–1^] 5.1 J cm^–2^	[mol s^–1^ g^–1^] 5.8 J cm^–2^	[mol s^–1^ g^–1^] Reference
175			0.89 × 10^–5^	1.45 × 10^–5^
200		0.78 × 10^–5^	2.13 × 10^–5^	4.30 × 10^–5^
250		1.84 × 10^–5^	6.26 × 10^–5^	13.1 × 10^–5^
300	1.28 × 10^–5^	4.23 × 10^–5^	11.35 × 10^–5^	32.5 × 10^–5^
350	3.06 × 10^–5^	7.92 × 10^–5^	17.80 × 10^–5^	36.3 × 10^–5^
400	6.39 × 10^–5^	12.9 × 10^–5^	24.56 × 10^–5^	18.4 × 10^–5^

A nominal Turnover Frequency (TOF) can be calculated by assuming that the active sites are exclusively located on a specific crystal plane, such as the (110) plane, and assuming all surfaces are (110). For rhombohedral LaCoO_3_, this *hkl* typically shows the highest intensity in X-ray diffraction (XRD) patterns, and was found in most of FFT analyses, indicating a preferred orientation and greater surface exposure. A summary of specific surface area (SSA) calculations is tabulated in Table S10. Knowing the area of the (110) unit cell (1.23 × 10^–18^ m^2^, containing 6 Co atoms) and the SSA of the LaCoO_3_ nanocatalyst, and considering the CO conversion at each temperature, nominal-TOF values were calculated as presented in Table S11. Up to 350 °C, the reference catalyst exhibited higher activity than the PLAL-synthesized nanoparticles. To investigate whether defects contribute to this observed performance difference, electron microscopy was performed on the reference catalyst (see below).

The CO_2_ selectivity (*S*_CO_2__) of the catalysts varied with respect to the preparation method and applied energy density. Selectivity values for PLAL-synthesized NPs and the reference catalyst are summarized in Table S12. PLAL-produced catalysts prepared at 5.8 and 5.1 J cm^–2^ yielded *S*_CO_2__ values of 100% up to 350 °C and 89% and 83% at 400 °C, respectively. For 4.5 J cm^–2^, *S*_CO_2__ was 100% up to 250 °C and 28% at 400 °C. In comparison, the reference catalyst was selective up to 300 °C, but exhibited a selectivity of only 79% at 350 °C and 29% at 400 °C, due to RWGS. This suggests that PLAL-synthesized perovskite NPs (5.1 and 5.8 J cm^–2^) are more selective to CO_2_ at the higher reaction temperatures. The defects introduced by PLAL synthesis alter the nanoparticle surface energetics by creating low-coordinated sites with higher local electron density. This electron-rich environment fundamentally changes the stability of the CO_2_ reaction intermediates, specifically the formate (HCOO*) or carboxyl (COOH*) species, by making the surface unfavorable for the C–O bond dissociation required to generate the undesirable CO, thus suppressing the RWGS reaction. A comparable finding shows that the stability of the HCOO* intermediate, which is chemically bonded to the defect site (e.g., oxygen vacancy), was crucial for selective CO_2_ hydrogenation, as it suppresses the side reaction to CO.^51^

### 3.4 Electron Microscopy Analysis of Chemically Synthesized LaCoO_3_

BF-TEM in Figure 7a shows that the chemically synthesized LaCoO_3_ NPs did not possess uniform shape, appearing rather polygonal, but they also contained facets. Selected area diffraction (SAED) yielded a diffraction pattern characteristic of rhombohedral LaCoO_3_. A summary of lattice distance analysis is shown in Table S13.

**Figure 7:**
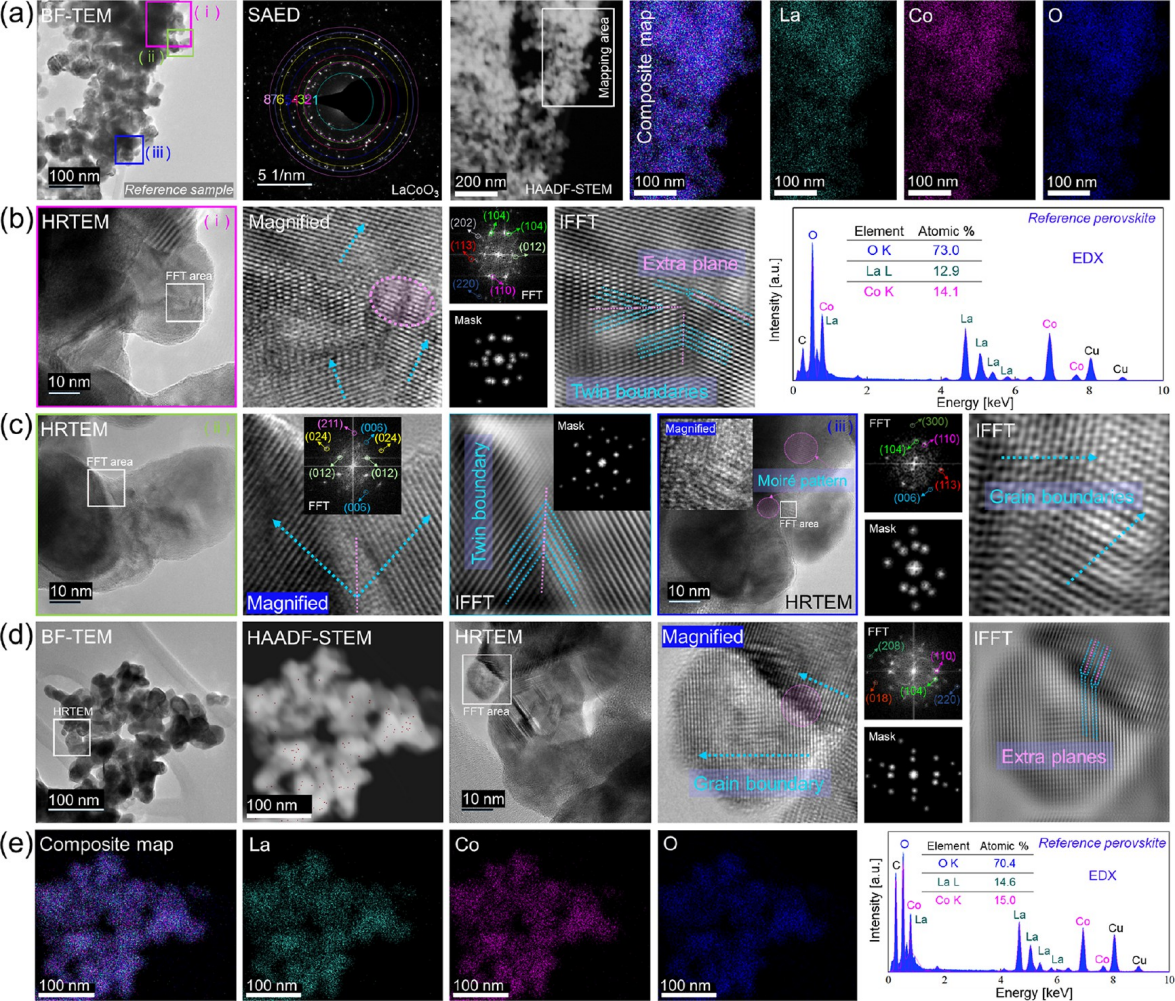
Electron microscopy and EDX mapping analysis of chemically synthesized LaCoO_3_ perovskite nanoparticles. (a) BF-TEM, SAED pattern, HAADF-STEM and composite map with individual EDX elemental maps for La, Co and O; (b) HRTEM (i), magnified area with FFT (inset), followed by its IFFT and a corresponding mask (inset) displays twin boundaries. An EDX spectrum is also provided; (c) HRTEM (ii) showing marked FFT region with magnified area as inset, FFT and corresponding mask and IFFT displays grain boundary defect; (d) BF-TEM image of another area, HAADF-STEM, HRTEM, magnified area, FFT/mask and its IFFT shows extra planes attributed to a grain boundary; (e) Composite EDX map, along with individual elemental maps for La, Co and O, and an associated EDX spectrum obtained from the BF-TEM area.

Furthermore, the EDX elemental map demonstrates a uniform distribution of La, Co, and O. HRTEM analysis, including FFT/spot mask and IFFT (Figure 7b), reveals the presence of grain and twin boundaries. FFT indicates reflections from (012), (110), (104), (113), (202) and (220) planes, characteristic of the rhombohedral phase. Quantitative analysis of the Co/La elemental ratio in the reference sample yielded 14:13. This compares to PLAL-synthesized NPs (5.8 J cm^–2^), which contain a slightly lower amount of La.

HRTEM (ii) in Figure 7c, including IFFT, reveals two grain boundaries converging to form a single twin boundary. FFT analysis shows reflections corresponding to the (012), (006), (024), and (211) planes. The HRTEM image (iii) displays Moiré patterns, different from crystallographic defects. FFT analysis indicates the presence of reflections from the (110), (104), (113), and (006) planes. IFFT clearly shows grain boundaries with differing atomic orientations coming together.

Figure 7d displays a BF-TEM image from another distribution of the reference sample. FFT shows reflections matching to the (110), (104), (018), (220) and (208) planes. The IFFT analysis shows the point where two nanoparticles with distinct atomic orientations have coalesced, resulting in extra planes at their grain boundary. EDX elemental mapping (Figure 7e) also reveals an even distribution of La, Co, and O.

Further analysis was conducted on the reference sample to assess the consistency of defect presence. A BF-TEM image showing an additional distribution of LaCoO_3_ is provided in Figure S34a. EDX mapping combined with HAADF-STEM (overlayer image) reveals a uniform distribution, consistent with other observed areas. HRTEM (i) and IFFT in Figure S34b further prove a grain boundary where two distinct atomic orientations have merged. The FFT pattern indicates the presence of the (110), (104), (113), and (220) planes. The IFFT of HRTEM (ii) also demonstrates a grain boundary where the coalescence of two nanoparticles has introduced extra planes. The (110), (104), and (220) planes are detected in the FFT pattern. An even distribution of La, Co, and O is evident in the EDX composite map (Figure S34d).

Altogether, the analysis of the reference catalyst revealed the presence of mainly grain boundaries and few twin boundaries, but no internal defects (frequent dislocations and twin boundaries), typical of laser-synthesized NPs. Thus, chemically synthesized LaCoO_3_ is not included in Table 1. For chemically synthesized NPs, calcination promotes the coalescence of irregularly shaped NPs, driven by minimization of surface energy. These newly formed grain boundaries likely act as active sites for catalytic activity.

### 3.5 Ex Situ Post-Reaction Analysis of Perovskite NPs

Since the fs-PLAL perovskite NPs produced at *F* 5.8 J cm^–2^ showed the highest activity, this catalyst was selected for postreaction electron microscopy to evaluate potential changes in shape, crystallinity and defect abundance. However, resolving the mechanistic contributions of defects and direct observation of nanocatalysts under realistic working conditions necessitates the future application of in situ and operando TEM analysis.^52^

BF-TEM and HAADF-STEM of two different areas in Figure 8a and b both show agglomeration of perovskite NPs. The composite EDX map shows a homogeneous distribution of LaCoO_3_ without the presence of phase segregation. This agglomeration slightly reduces the exposed surface area. Still, MS data of the catalyst stability test of perovskite NPs produced at 5.8 J cm^–2^ (Figure S35) revealed no deactivation even over 2 days, thus confirming the continued availability of active sites for catalytic reaction.

**Figure 8:**
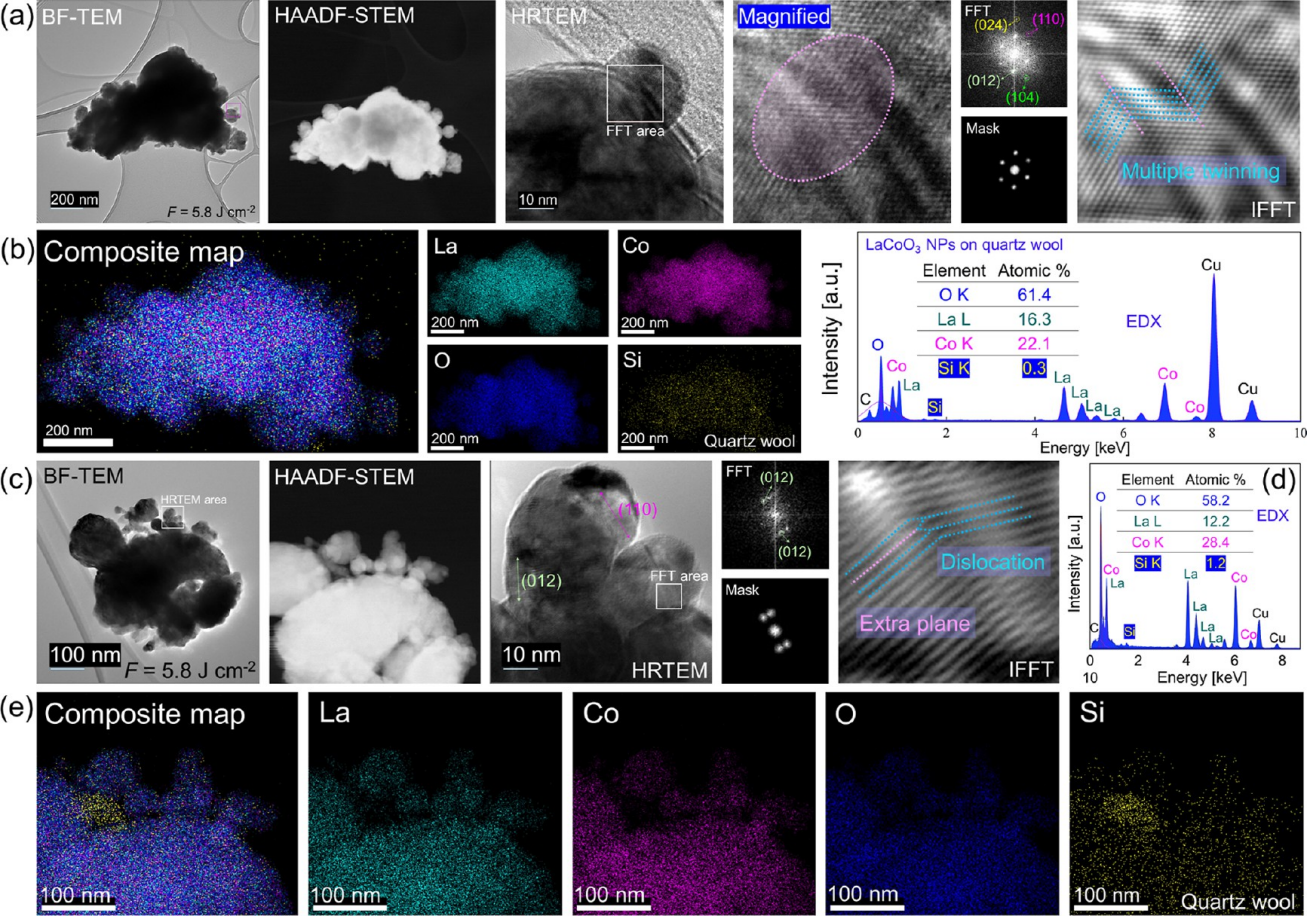
Postreaction TEM and EDX analysis of LaCoO_3_ perovskite NPs, femtosecond laser-produced (5.8 J cm^–2^), on quartz wool support. (a) BF-TEM, HAADF-STEM, HRTEM, magnified view, FFT/spot mask and IFFT image. (b) EDX composite map with individual elemental maps (La, Co, O and Si) and the corresponding EDX spectrum. (c) BF-TEM, HAADF-STEM, HRTEM, FTT/spot mask and IFFT image from another region. (d) EDX spectrum and inset table detailing (at %) composition corresponding to the BF-TEM image in (c). (e) EDX composite map and individual elemental maps for La, Co, O and Si.

The FFT patterns in Figure 8a reveal reflections from (012), (110), (104), and (024) planes, characteristic of the rhombohedral phase. In contrast, the FFT pattern in Figure 8b primarily reflects (012), which, given the complexity of defects present (dislocation), suggests a pseudorhombohedral phase. EDX elemental mapping indicates uniform distribution of La, Co and O on quartz wool. However, the agglomeration of nanoparticles prevents the observation of cobalt-rich structures. Nevertheless, line scans (Figure S36) taken from areas shown in Figure 8a and c also indicate comparably higher Co fraction, consistent with the EDX data. HRTEM combined with FFT/IFFT analysis confirms the presence of defective zones such as multiple twinning (Figure 8a) and dislocations resulting from extra planes (Figure 8c), even after pretreatment and the catalytic reaction.

## 4 Conclusions

Utilizing femtosecond laser pulses for the green synthesis of LaCoO_3_ model nanocatalysts resulted in the formation of two distinct nanoperovskite structures: stoichiometric LaCoO_3_ and nonstochiometric Co-rich nanoparticles. The consistently slightly higher atomic percentage of cobalt than lanthanum observed in quantitative EDX analysis indicates the existence of cobalt-rich zones.

Micro-Raman spectroscopic analysis revealed several Raman active modes in both laser-synthesized nanoparticles and the pristine target. This suggests that the crystal structure deviates from the perfect cubic symmetry, in line with a rhombohedral phase. This is further supported by the understanding that perfect cubic perovskites are basically Raman inactive due to symmetry rules. In addition, Micro-Raman data revealed a significant enhancement of second-order phonon scattering modes in laser-synthesized nanoparticles which correlate with a high defect concentration. XRD and FFT patterns further confirmed the presence of nanoparticles with rhombohedral lattice. However, FFT analysis also revealed that in certain regions, due to localized atomic disorder, specifically those associated with extra planes and dislocations, the nanoparticles exhibit a structure closer to pseudorhombohedral, with lower symmetry than rhombohedral.

The spatial distribution of defects was further proven by high-resolution microscopy in conjunction with Fourier filtering and inverse fast Fourier transform image reconstruction. LaCoO_3_ exhibited a high density of defects, including grain boundaries, twinning (e.g., single and multiple), and dislocations. Defect formation originates from the rapid condensation of primary nanoparticles initiated by high cooling rates during fs-PLAL. This reveals a relationship between laser fluence and the defect density in laser-produced nanoparticles. Thus, an increase in laser fluence resulted in an increased number of defects. This correlation stems from the fact that higher fluence yields more energy per pulse, which, in turn, increases the temperature gradient and drives a more rapid and explosive plasma expansion, followed by the fast quenching intrinsic to femtosecond pulses. This enhanced cooling further restricts the time available for crystalline relaxation, permanently incorporating a larger number of nonequilibrium defects (twinning and dislocations) into the final nanoparticle structure.

Analysis of chemically synthesized LaCoO_3_ NPs revealed the existence of grain and twin boundaries, originating from nanoparticle coalescence, but not of internal defects. The calcination process and thermal treatment facilitate merging of irregularly shaped NPs to lower their surface energy, with the resulting grain boundaries likely acting as active sites for catalytic activity.

Still, the high defect density in PLAL-LaCoO_3_ resulted in a higher catalytic activity. The highest CO conversion was observed for nanoparticles produced at 5.8 J cm^–2^, with activity setting in around 175 °C, a better performance than for other laser fluences. Compared to the corresponding chemically synthesized LaCoO_3_ catalyst, PLAL-synthesized LaCoO_3_ produced at 5.8 J cm^–2^ showed the highest CO_2_ selectivity of 89% and CO conversion above 350 °C, indicating best performance within the 350–400 °C range.

Postreaction analysis revealed agglomeration of PLAL-perovskite NPs, but with no observable phase separation. Furthermore, stability tests showed no deactivation even over 2 days, indicating sustained active site availability. The ability to generate defect-rich nanostructures through femtosecond laser ablation offers a promising path for tailoring catalytic activity in an environmentally friendly manner. Further investigations into the scalability of laser-produced catalysts are necessary to fully comprehend their industrial impact.

The current study mainly focused on visualizing PLAL-induced planar and linear defects using high-resolution microscopy. Furthermore, due to the transient nature of oxygen vacancies and their high propensity for healing under O_2_ flow, studying the direct, steady-state catalytic contribution of these oxygen deficiencies is challenging. Clearly, further complementary techniques such as X-ray Photoelectron Spectroscopy (XPS), Electron Paramagnetic Resonance (EPR) or Electron Energy Loss Spectroscopy (EELS) can be essential for chemical state and point defect analysis. Furthermore, in situ/operando TEM is very promising for future research to clarify the actual mechanistic role of defects and to directly observe nanocatalysts under realistic working conditions.

## Supplementary Material



## Notes

Detailed methodology of laser fluence assessment, characterization methods, profilometry analysis of LaCoO_3_ perovskite targets before and after laser ablation, SEM, XRD and μ-Raman spectroscopy, electron microscopy TEM/HRTEM/HAADF-STEM/SAED/FFT/IFFT/EDX of defect-rich LaCoO_3_ perovskite nanoparticles, reference catalyst and postreaction nanoparticles (fs-synthesized at 5.8 J cm^–2^), size distribution analysis, MS data and calculations including defect density, CO_2_ selectivity, specific activity, specific surface area and nominal TOF (PDF)
