# Supplementary material for: Femtosecond Laser Generation of LaCoO3 Perovskite Nanocatalysts for Preferential CO Oxidation
Source: ACS Appl Nano Mater. 2025 Oct 30;8(45):21948–63. doi: 10.1021/acsanm.5c03993 (PMC12624525; doi:10.1021/acsanm.5c03993)
Supplement: Supplementary file 1 [file an5c03993_si_001.pdf]

Supporting Information

Femtosecond Laser Generation of $LaCoO_3$ Perovskite Nanocatalysts for Preferential CO Oxidation

Niusha Lasemi [1], Nevzat Yigit [1], Gerhard Liedl [2], Jürgen Fleig [3] and Günther Rupprechter [1]*

[1] Institute of Materials Chemistry, TU Wien, 1060 Wien, Austria

[2] Institute of Production Engineering and Photonic Technologies, TU Wien, 1060 Wien, Austria

[3] Institute of Chemical Technologies and Analytics, TU Wien, 1060 Wien, Austria

Correspondence to: guenther.rupprechter@tuwien.ac.at

Note:

The supplementary PDF offers more insights into the analysis of $LaCoO_3$ perovskite targets post-ablation, a description of the experimental equipment and further characterization of perovskite nanoparticles using electron microscopy techniques, presented through detailed discussion, figures, and tables. At the end, one will find the relevant references for the supplementary material.

1. Fluence assessment

Optical microscopy using Zeiss AxioVision software was applied to measure the squared diameter (D^2) of the ablated zones on $LaCoO_3$ target at various laser fluences (F). This measurement averaged 30 craters. The goal of varying F was to determine the optimal conditions for producing nanoparticles with high defect density and achieving high productivity.

Given a Gaussian beam profile, the relationship between D^2, Gaussian beam radius (w_0), threshold fluence (F_{th}) can be described by equation 1.[1] Figure S1 shows the relationship between D^2 as a function of F. Linear fitting provided an evaluated w_0 of 53.35 μm and F_{th} of 1.25 J cm^{-2}. Based on the equation 2, all the fluences are measured and summarized in Table S1.

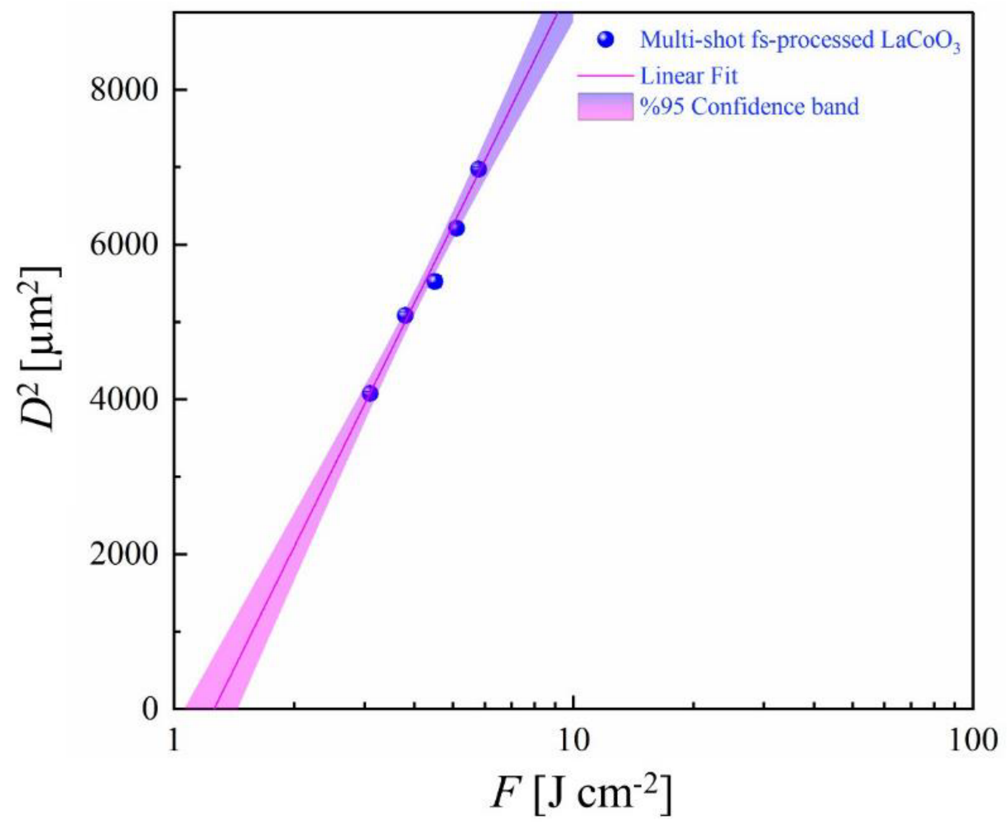

Figure S1. Squared diameter of the ablation area on $LaCoO_3$ perovskite target versus laser fluence (D^2 versus F) in ethanol (N_{1000}, 30 fs, 1 kHz) utilizing optical microscopy. The 95% confidence interval of the linear fit is shown as a colored zone.

Eq. 1 $$D^2 = 2w_0^2 \ln(\frac{F}{Fth})$$

Eq. 2 $$F = (\frac{2E}{\pi w0^2})$$

Table S1. Details the calculated *F* used for femtosecond laser ablation of a $LaCoO_3$ target in ethanol (30 fs, 1 kHz, 800 nm)

Power (mW)	Energy (mJ)	Fluence (J cm^{-2})
140	0.14	3.1
170	0.17	3.8
200	0.20	4.5
230	0.23	5.1
260	0.26	5.8

2. Characterization methodology

2.1. Optical Microscopy

Analysis of crater diameters was performed using an Olympus GX51 inverted metallurgical microscope equipped with an XY mechanical stage. A 20x objective lens was used for imaging. The microscope has a maximum magnification of ×1000 and is equipped with interchangeable lenses (×5, ×10, ×20, ×50, ×100). A Zeiss camera was connected to the microscope for recording.

2.2. Profilometry

A Bruker DektakXT stylus profilometer, featuring a conical diamond tip with a 2 μm radius, was utilized to measure the ablation depth and create three-dimensional (3D) maps of the $LaCoO_3$ craters. The analysis was conducted with a stylus force of 3 mg, and a trace resolution of 12 μm was applied during profilometry to improve the 3D mapping quality.

2.3. Scanning electron microscopy (SEM)

A JCM-6000 PLUS Bench Top SEM (JEOL) was used for further analysis of crater dimensions, shape, surface morphology and microstructures of the $LaCoO_3$ perovskite targets before and after laser ablation. Low vacuum mode (10 kV) with backscattered electron (BSE) imaging was performed to enhance the observation.

2.4. X-ray diffraction (XRD)

X-ray diffraction (Philips XPERT-PRO diffractometer) with Cu K-α radiation (λ = 1.5406 Å) was employed for phase analysis. The data were collected in Bragg-Brentano reflection geometry with operating conditions of 40 kV and 40 mA, over a 2θ range of 5° to 100° with a step size of 0.02° and a dwell time of 0.40 second per step. The obtained diffraction patterns were then analysed for phase identification and subjected to Rietveld refinement using the High Score Plus software.

2.5. Dynamic light scattering (DLS)

A compact goniometer system (ALV/CGS-3) equipped with a helium-neon (He-Ne) laser at a wavelength of 632.8 nm and a power of 22 mW was used for DLS measurements. These measurements were performed on colloidal $LaCoO_3$ perovskite nanoparticles at room temperature and a scattering angle of 90° to ensure a high signal-to-noise ratio. The mean Stokes radius (Rs) was determined by

applying Contin analysis, which involves a regularized fit (unweighted) to the intensity autocorrelation function.

2.6. UV/Vis spectroscopy

To investigate the optical properties of the colloidal perovskite nanoparticles, the colloidal solutions were injected into a high-precision quartz cuvette with a 10 mm path length (sourced from Hellma Analytics) and later analyzed by using a VWR® UV-1600PC UV/Visible spectrophotometer. This single-beam instrument, equipped with a tungsten lamp as a light source, operates within a wavelength range of 300 to 1100 nm (with an accuracy of ±0.3 nm and a wavelength accuracy of ±0.5 nm). It features a spectral bandwidth of 4 nm with a silicon photodiode detector, and a 1200 lines/mm grating.

2.7. Confocal micro-Raman spectroscopy

Raman spectra of the samples including target and nanoparticles were acquired at room temperature via using a Horiba XploRA™ INV confocal micro-Raman system. This system includes a thermoelectrically cooled charge-coupled device (CCD) detector and a motorized stage for precise measurements. Considering the potential for sample damage at higher laser intestines, the 532 nm excitation laser was used at a reduced intensity of 10%. For nanoparticle analysis, samples were placed in glassy-bottom Ibidi μ-dishes and illuminated with a laser beam through a 40x dry objective (Nikon Eclipse TiU). For solid target analysis a 10x objective was used. After full system calibration, spectra were collected from 0 to 3300 cm^{-1} using a holographic grating (1200 grooves/mm), a 200 μm slit, and a 300 μm hole. The LapSpec 6 spectroscopy suite software (HORIBA Scientific) was used to acquire all Raman spectra. Raman point mapping was done by selecting five random positions on the powder samples. To enhance the quality of the spectra, an acquisition time of 1 second and 15 accumulations were applied for each point. Signal processing and peak smoothing were then performed using the Adjacent-Averaging method with a window size of 30 and a polynomial order of 2.

2.8. Transmission electron microscopy

Transmission electron microscopy (TEM) using a FEI Tecnai F20 S-TWIN was employed for analysis of femtosecond produced $LaCoO_3$ perovskite NP at various F. High-resolution TEM (HRTEM) was operated at 200 kV. Bright field (BF) and dark field (DF) TEM imaging were used to determine the size and shape of NPs. Elemental mapping was performed using energy-dispersive X-ray spectroscopy (EDX) in scanning TEM (STEM) mode with a high-angle annular dark-field (HAADF) detector. Fast Fourier transform (FFT), inverse FFT (IFFT), and selected area electron diffraction (SAED) patterns

were analyzed using the Gatan Microscopy Suite (DigitalMicrograph) software. Lattice spacings (d) and Miller indices (hkl) were identified with HighScore software (ICDD database).

3. $LaCoO_3$ target analysis

The presented study contains the ablation of $LaCoO_3$ targets immersed in ethanol using a near-infrared femtosecond laser operating across a range of fluences. In each experimental run, polished samples were positioned at the bottom of a newly designed aluminum body cell (Figure 1) filled with ethanol and subjected to 1000 laser pulses. All ablation processes were conducted at the laser focal point to maintain a zero-defocusing condition. A comprehensive set of analytical techniques was employed to characterize both the targets before and after the femtosecond laser irradiation, as well as the resulting perovskite nanoparticles. Profilometry, optical microscopy and SEM were utilized to visualize the three-dimensional morphology of the ablation craters and to precisely measure their diameters. The chemical composition of targets was determined using confocal micro-Raman spectroscopy. Finally, the crystallinity of the pristine target surface was examined via X-ray diffraction.

3.1. Profilometry

Profilometry and optical microscopy provided insights into the perovskite ablation craters, allowing for the quantification of their depth, ablation volume, and specific ablation rate (as detailed in Figure S2a-h). Given the craters conical morphology, a cone volume calculation was employed. An inverse relationship between laser fluence and crater depth was observed; as F increased, the depth reduced, while the crater diameter expanded.

Three-dimensional imaging revealed multiple scatterings at the bottom of the ablation craters. This scattering became increasingly pronounced with higher F, leading to complex internal reflections, increased liquid turbulence, and laser beam deviation as a result of interactions with the ejected NPs.[2] The maximum ablated volume was observed at F: 5.8 J cm^{-2} corresponding to the pulse energy of 260 μJ. Previous investigations on Au nanoparticle generation reported maximal ablation efficiency at a pulse energy of 200 μJ.[3] Similarly, studies focusing on femtosecond laser processing of Au-coated Ni,[4] SiO_2[5] and CuZn[6] identified 250 μJ as the pulse energy associated with the highest ablation rates. Therefore, nonlinear processes, such as filamentation, did not adversely impact nanoparticle productivity within these fluence ranges.

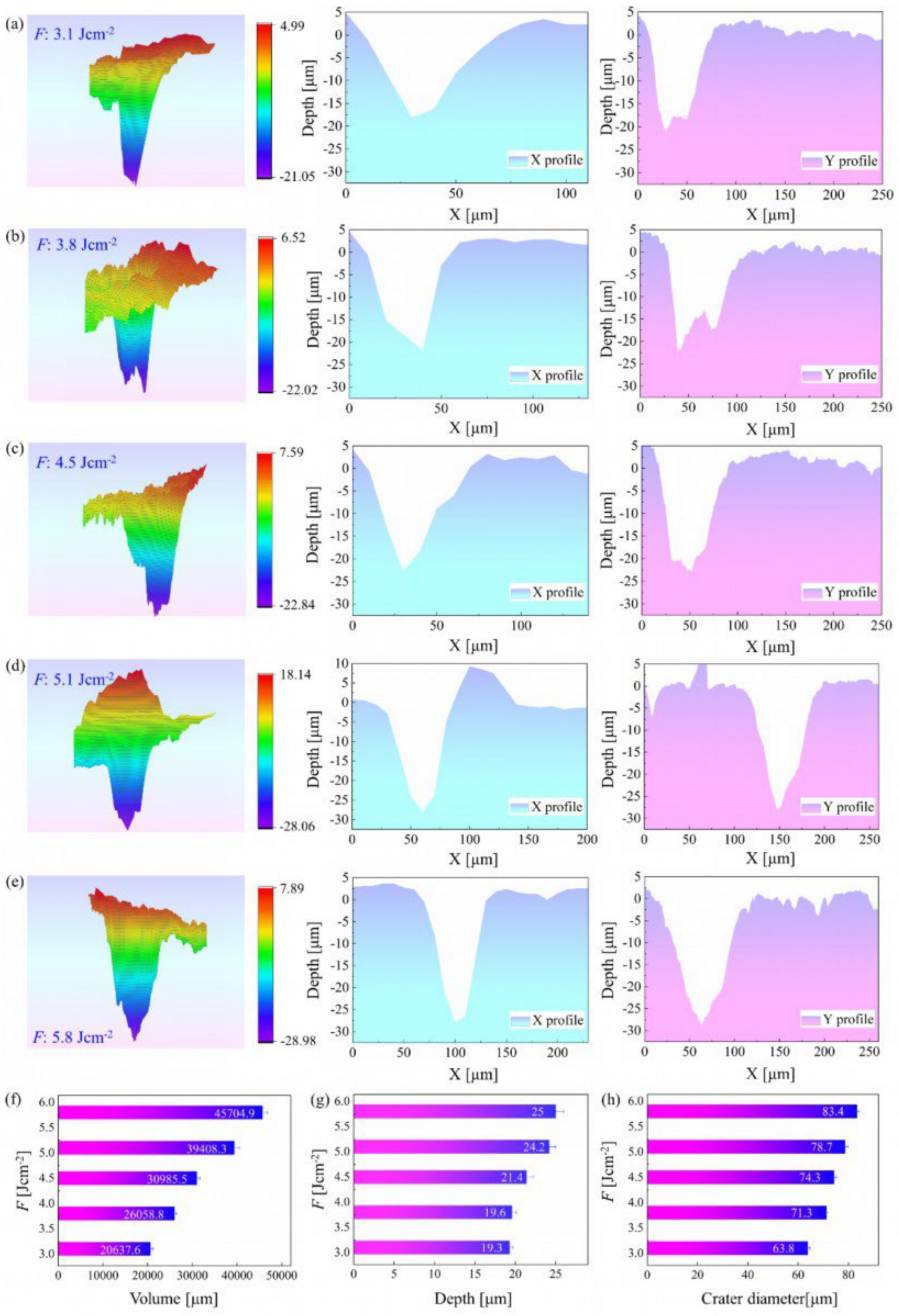

Figure S2. Three-dimensional crater mapping of $LaCoO_3$ perovskite target processed by femtosecond laser (30 fs, 1 kHz, N_{1000}, ethanol) at various *F* with their corresponding XY profiles; (a) *F*: 3.1 J cm^{-2}; (b) *F*: 3.8 J cm^{-2}; (c) *F*: 4.5 J cm^{-2}; (d) *F*: 5.1 J cm^{-2}; (e) *F*: 5.8 J cm^{-2}; (f) Ablated volume versus *F*; (g) Crater depth versus *F*; (h) Crater diameter versus *F*.

3.2. SEM

To determine the minimum laser fluence required to initiate ablation in $LaCoO_3$ immersed in ethanol, several laser fluences were established until the minimum fluence that induced microstructures on the surface, similar to specifically laser-induced surface structures, was identified. Unlike previous research that employed linearly polarized light and resulted in the formation of laser-induced periodic surface structures (LIPSS) on Au-coated Ni,[7] circularly polarized light was used here, leading to the formation of periodic micro-bumps. SEM images of craters at various F are shown in Figure S3a. Figure S3b displays microbombs with a magnified view. Figure S3c shows the profile of these microbombs, with a width of approximately 100 μm. Figure S3d illustrates the boundary between the untreated $LaCoO_3$ target and the laser-treated region.

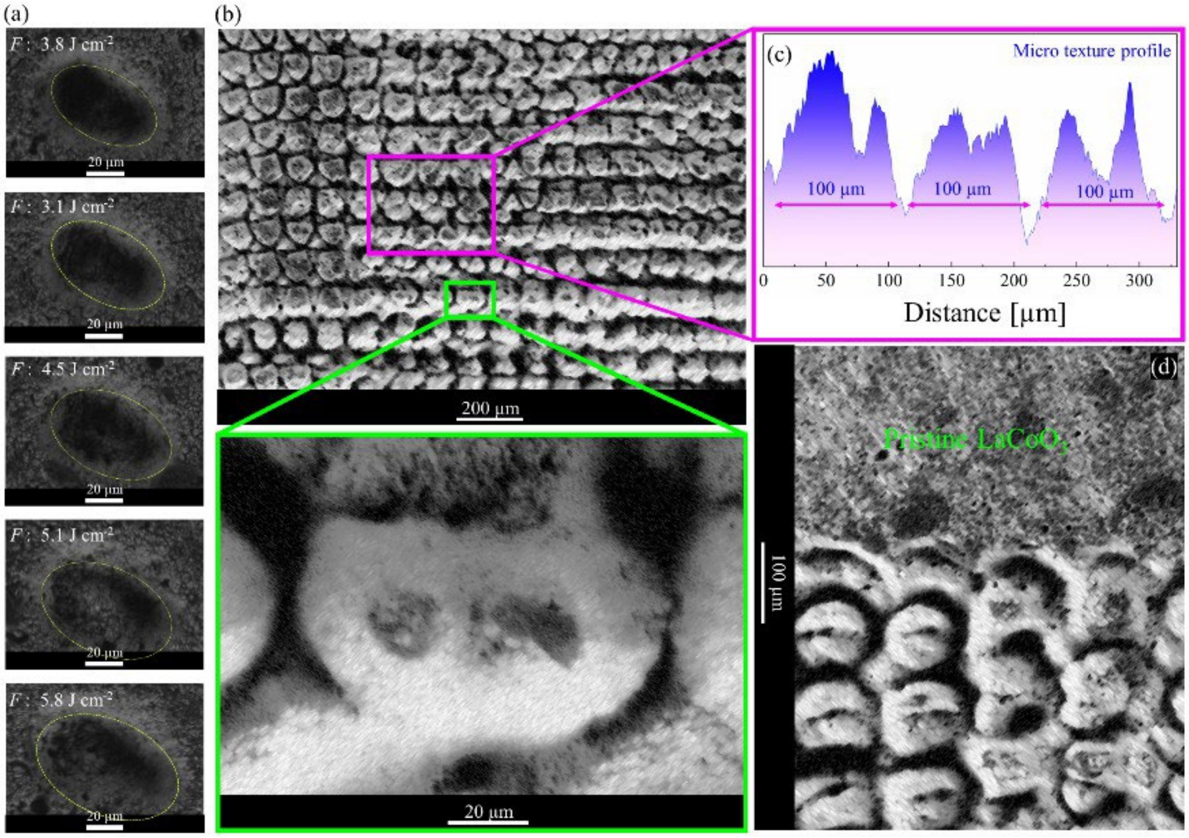

Figure S3. SEM images of ablation craters and surface modifications. (a) $LaCoO_3$ surface processed by femtosecond laser radiation in ethanol at various F (right side of image); (b) Microstructures (micro bumps) produced with femtosecond laser using circularly polarized light near the ablation threshold fluence, along with a magnified image; (c) Surface micro-texture profile of the laser-induced structures; (d) Border region between the pristine $LaCoO_3$ target and the micro structured zone.

3.3. X-ray diffraction

The X-ray diffraction pattern (Figure S4) obtained from the pristine sample displayed prominent reflections that closely matched the characteristic peaks of rhombohedral $LaCoO_3$, as indexed by the International Centre for Diffraction Data (ICDD) Powder Diffraction File (PDF) No: 04-006-2093.

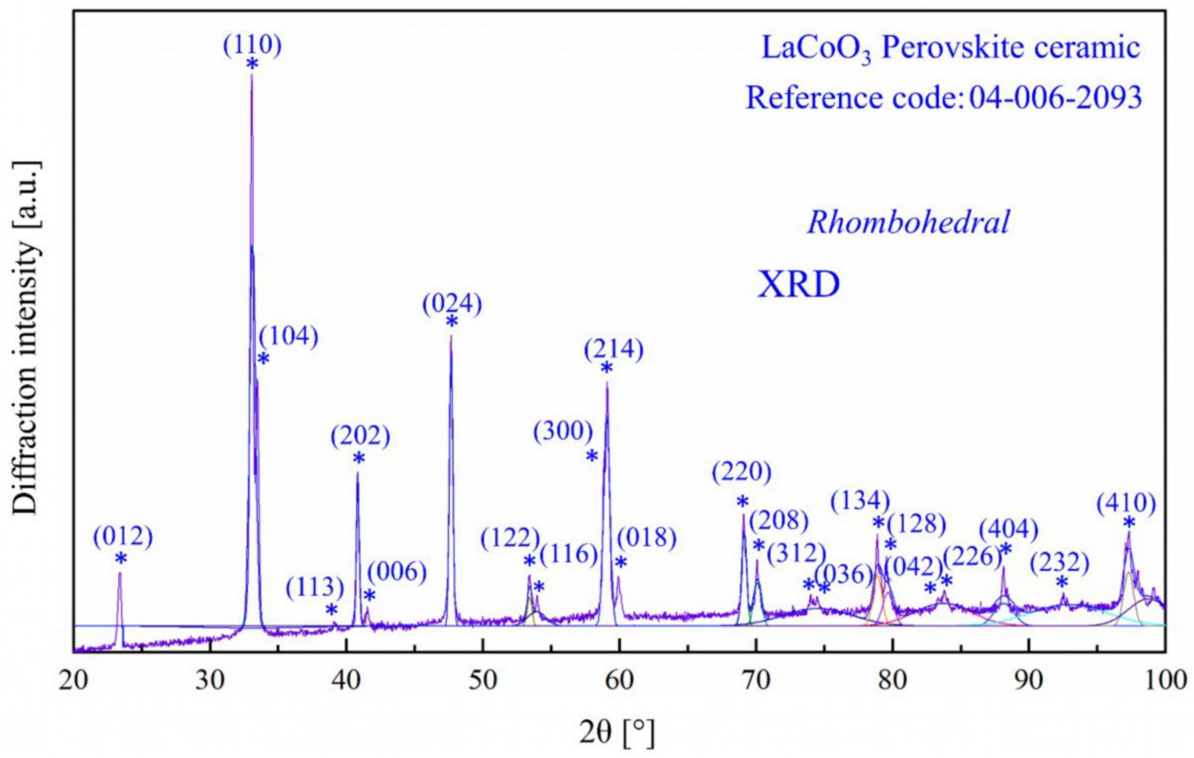

Figure S4. X-ray diffraction pattern obtained from the pristine $LaCoO_3$ target. Applying a Gaussian function to fit the experimental data produced cumulative fits.

3.4. Confocal µ-Raman spectroscopy

Figure S5 presents a Micro-Raman survey of the pristine and ablated areas of $LaCoO_3$, along with optical microscopy images of the craters ablated at various laser fluences. Second-order phonon excitations in the perovskite appeared normal for pristine and ablated areas and did not show the highly intense peaks similar to $LaCoO_3$ nanoparticles.

The A_{1g} mode (Figure S6) was detected at ~271 cm^{-1} for the pristine sample, while for the laser-ablated areas, it appeared at ~282 cm^{-1}. The Eg mode was detected at ~94 cm^{-1} for both the pristine and ablated areas. The Eg bending modes were detected at ~436 and ~444 cm^{-1}, for the pristine target and ablated craters, respectively. The Eg quadrupole mode was detected at ~547 cm^{-1} for the pristine sample, exhibiting a clear peak, while for the ablated areas, it is assumed to be present within a broader peak. The A_{2g} breathing mode was also detected at ~612 cm^{-1} for pristine and red shifted to ~600 cm^{-1} for

ablated areas. Forbidden A_{2g} modes may appear because of formation of distortion or defects in the bulk.

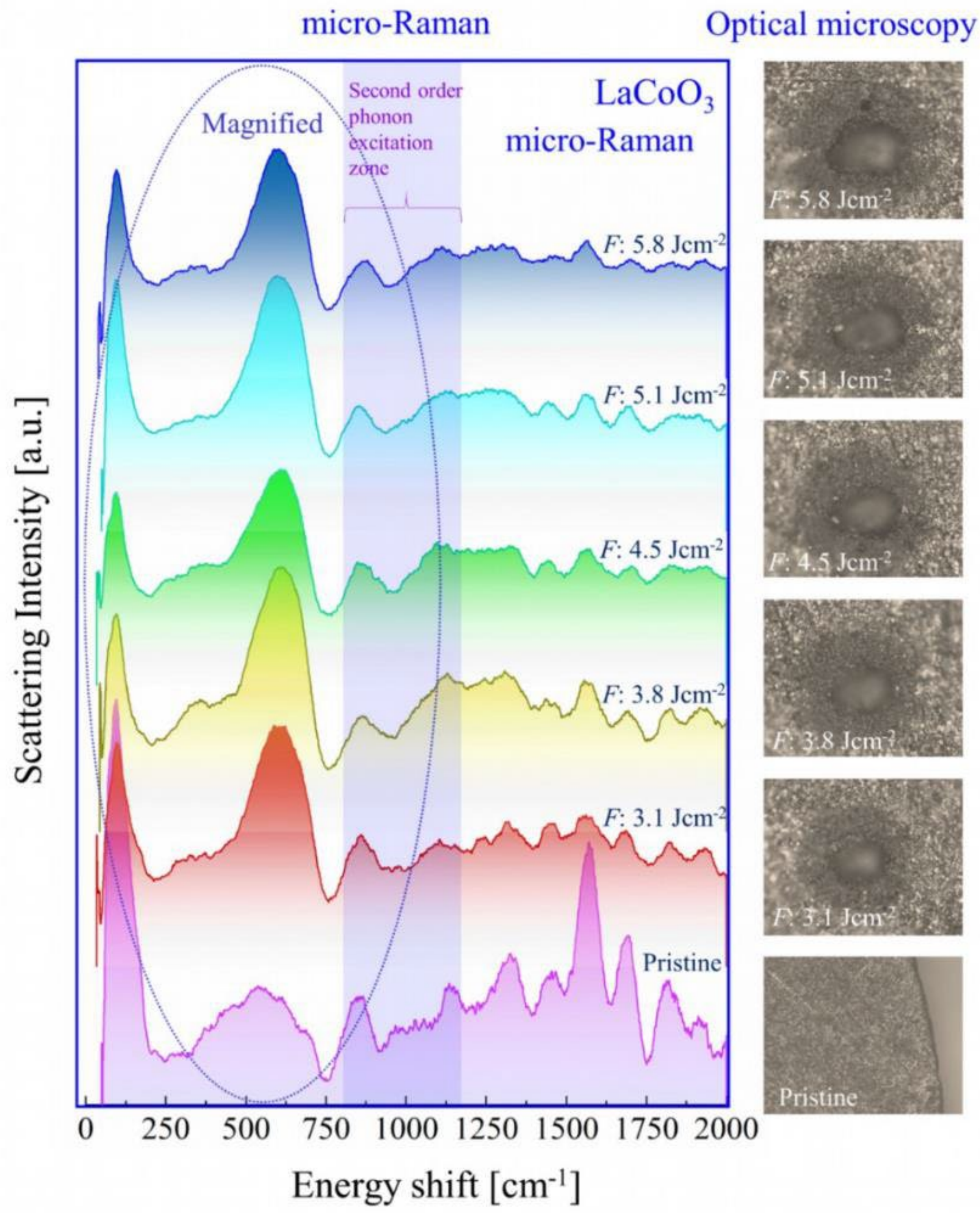

Figure S5. Confocal micro-Raman survey of pristine and femtosecond laser-processed $LaCoO_3$ at various fluences, along with corresponding optical images.

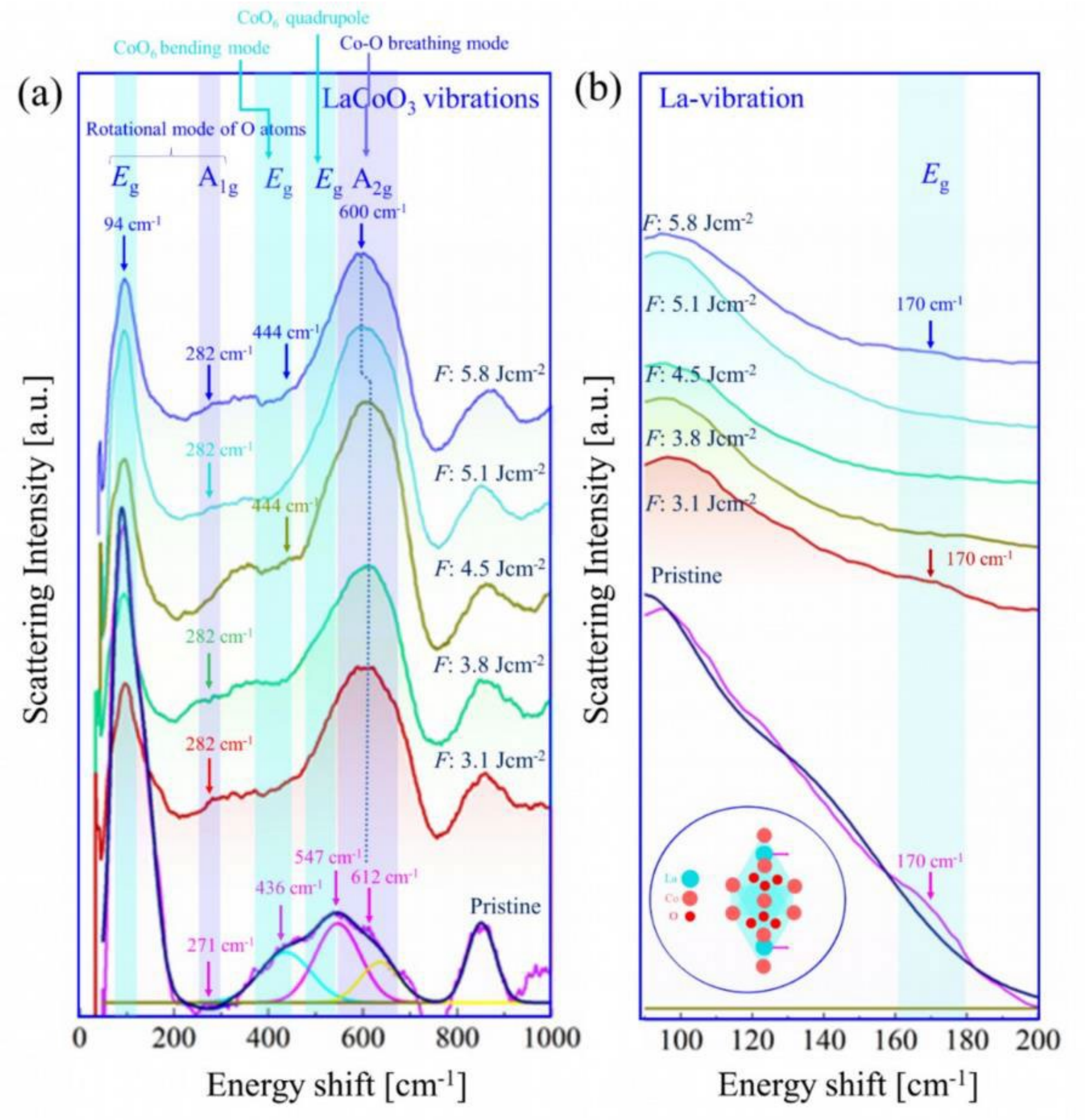

Figure S6. Micro Raman scattering of pristine and $LaCoO_3$ perovskite target femtosecond laser-processed at various laser *F*. (a) $LaCoO_3$ vibrations including rotational modes for O atoms and Co-O bending, quadrupole and breathing mode; (b) Magnified area showing La-vibration mode.

4. $LaCoO_3$ nanoparticle analysis

4.1. TEM/HRTEM/STEM/EDX of $LaCoO_3$ NPs

Table S2. Elemental Composition of $LaCoO_3$ perovskite nanoparticles femtosecond laser-produced via EDX mapping (*F*: 5.8 J cm^{-2}) related to Figure 2c.

Element	Atomic %
O K	70.4
La L	14.5
Co K	15

Table S3. Elemental Composition of $LaCoO_3$ perovskite nanoparticles femtosecond laser-produced via EDX mapping (*F*: 5.8 J cm^{-2}) related to Figure 2e.

Element	Atomic %
O K	64.7
La L	13.4
Co K	21.9

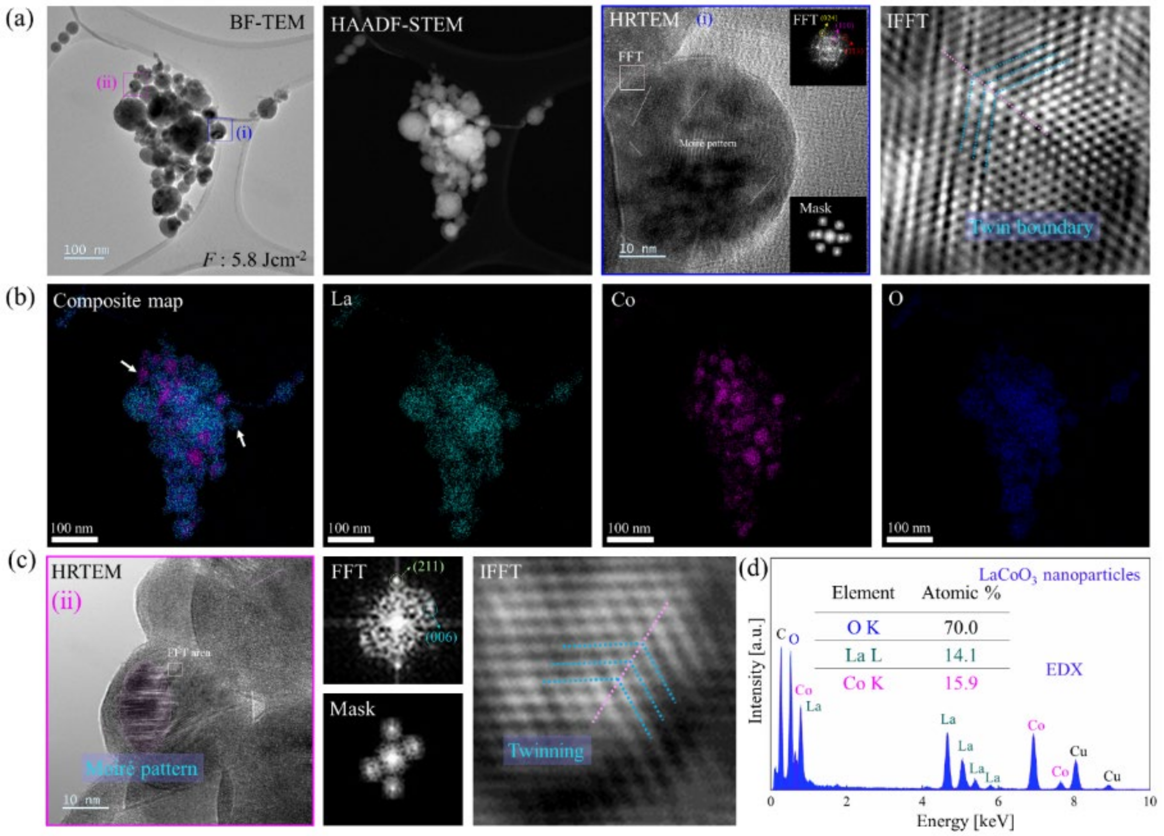

Figure S7. Electron microscopy and EDX mapping analysis of $LaCoO_3$ perovskite nanoparticles femtosecond laser-produced (*F*: 5.8 J cm^{-2}). (a) BF-TEM, HAADF-STEM, HRTEM (i) with its FFT and mask (inset) and IFFT indicating a single twin boundary; (b) A composite EDX map with it corresponding individual EDX elemental maps for La, Co and O; (c) HRTEM (ii) with FFT as inset, FFT/spot mask and IFFT showing twinning; (d) EDX spectrum obtained from the BF-TEM area.

Figure S8 shows a BF-TEM image of the area containing core-shell perovskite NPs, a structure confirmed by the EDX elemental map. The EDX map for cobalt reveals a cobalt-rich core. The line scan analysis further distinguishes two types of NPs: stoichiometric $LaCoO_3$ (Figure S8c) and non-stoichiometric cobalt-rich core NPs with a stoichiometric $LaCoO_3$ shell. EDX spectrum and table of atomic% contribution (inset) are shown in Figure S8d.

Figure S9 shows a BF-TEM image and a HAADF-STEM image. The corresponding EDX map indicates a homogenous distribution of elements. However, the cobalt elemental map specifically reveals that smaller NPs within the imaged area exhibit a higher cobalt concentration. The EDX spectrum obtained from the BF-TEM area, along with the table of atomic% contributions (inset), are shown in Figure S9d.

The composite map in Figure S10a shows a uniform distribution of La, Co, and O elements within the imaged area. The EDX spectrum obtained from the BF-TEM area, along with the table of atomic% contributions (inset), are shown in Figure S10b.

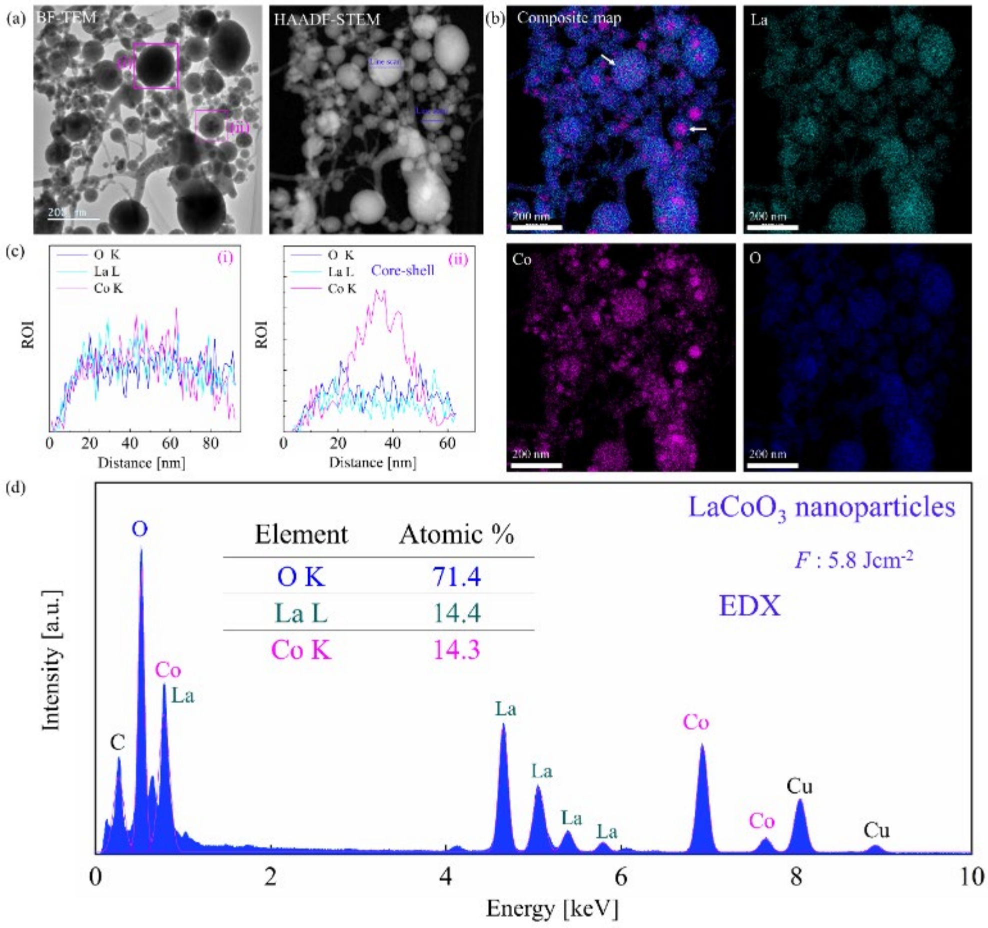

Figure S8. Electron microscopy and EDX mapping analysis of $LaCoO_3$ perovskite nanoparticles femtosecond laser-produced (*F*: 5.8 J cm^{-2}). (a) BF-TEM and HAADF-STEM images; (b) Composite EDX mapping image showing individual EDX elemental maps for La, Co, and O; (c) EDX spectrum acquired from the BF-TEM area.

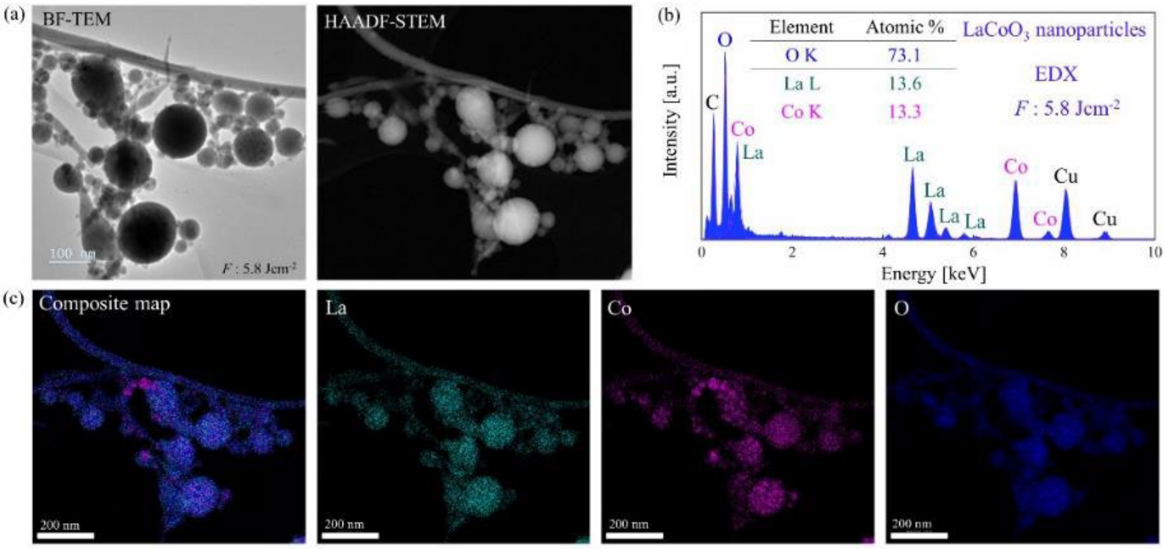

Figure S9. Electron microscopy and EDX mapping analysis of $LaCoO_3$ perovskite nanoparticles femtosecond laser-produced (*F*: 5.8 J cm^{-2}). (a) BF-TEM and HAADF-STEM images; (b) EDX spectrum acquired from the BF-TEM area; (c) Composite EDX mapping image showing individual EDX elemental maps for La, Co, and O.

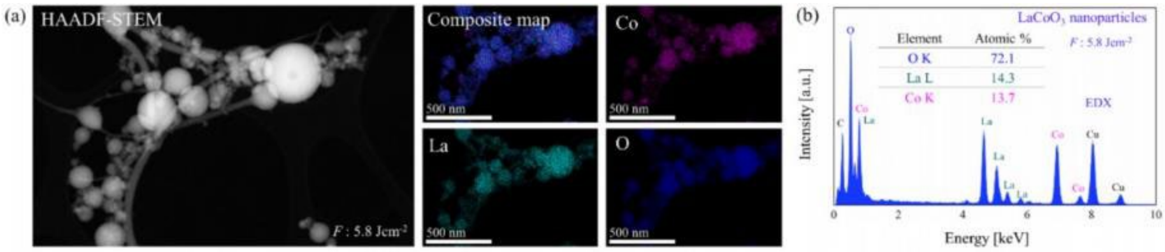

Figure S10. Electron microscopy and EDX mapping analysis of $LaCoO_3$ perovskite nanoparticles femtosecond laser-produced (*F*: 5.8 J cm^{-2}). (a) HAADF-STEM image and a composite EDX mapping image showing individual EDX elemental maps for La, Co, and O; (b) EDX spectrum acquired from the HAADF-STEM area.

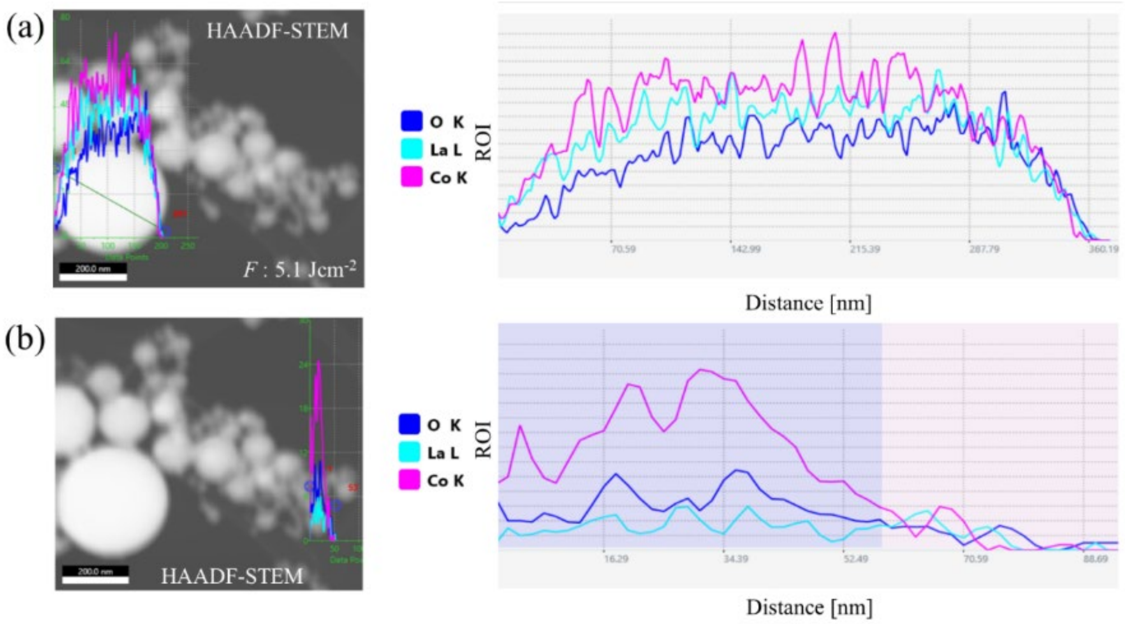

Figure S11. HAADF-STEM images and corresponding line scan profiles of $LaCoO_3$ perovskite nanoparticles femtosecond laser-produced (*F*: 5.1 J cm^{-2}), related to Figure 3c.

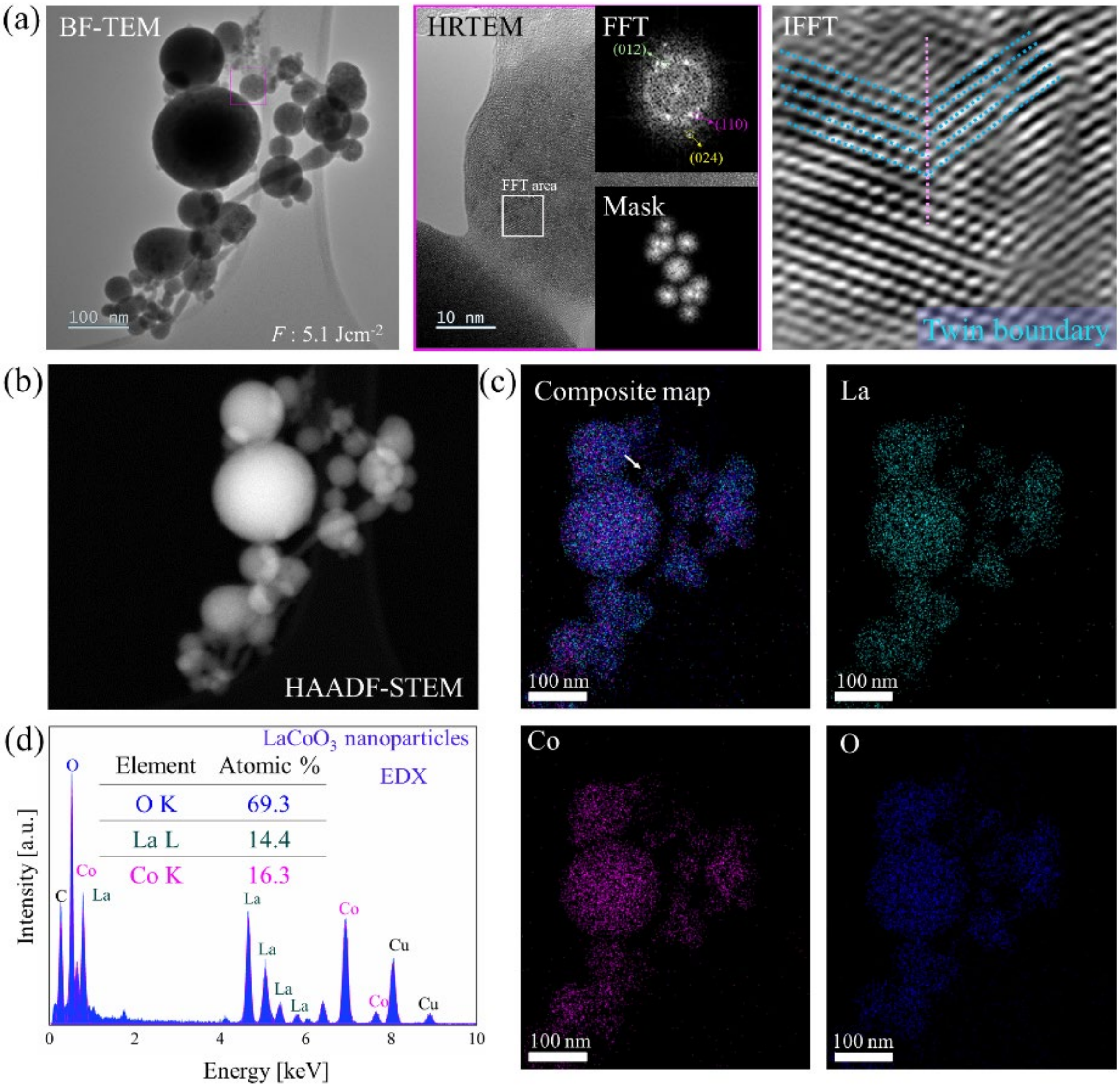

Figure S12. Electron microscopy and EDX mapping analysis of $LaCoO_3$ perovskite nanoparticles femtosecond laser-produced (*F*: 5.1 J cm^{-2}). (a) BF-TEM, HRTEM with FFT/spot mask (inset) and IFFT showing single twining; (b) HAADF-STEM image; (c) A composite EDX mapping image showing individual EDX elemental maps for La, Co, and O; (d) EDX spectrum obtained from the BF-TEM area.

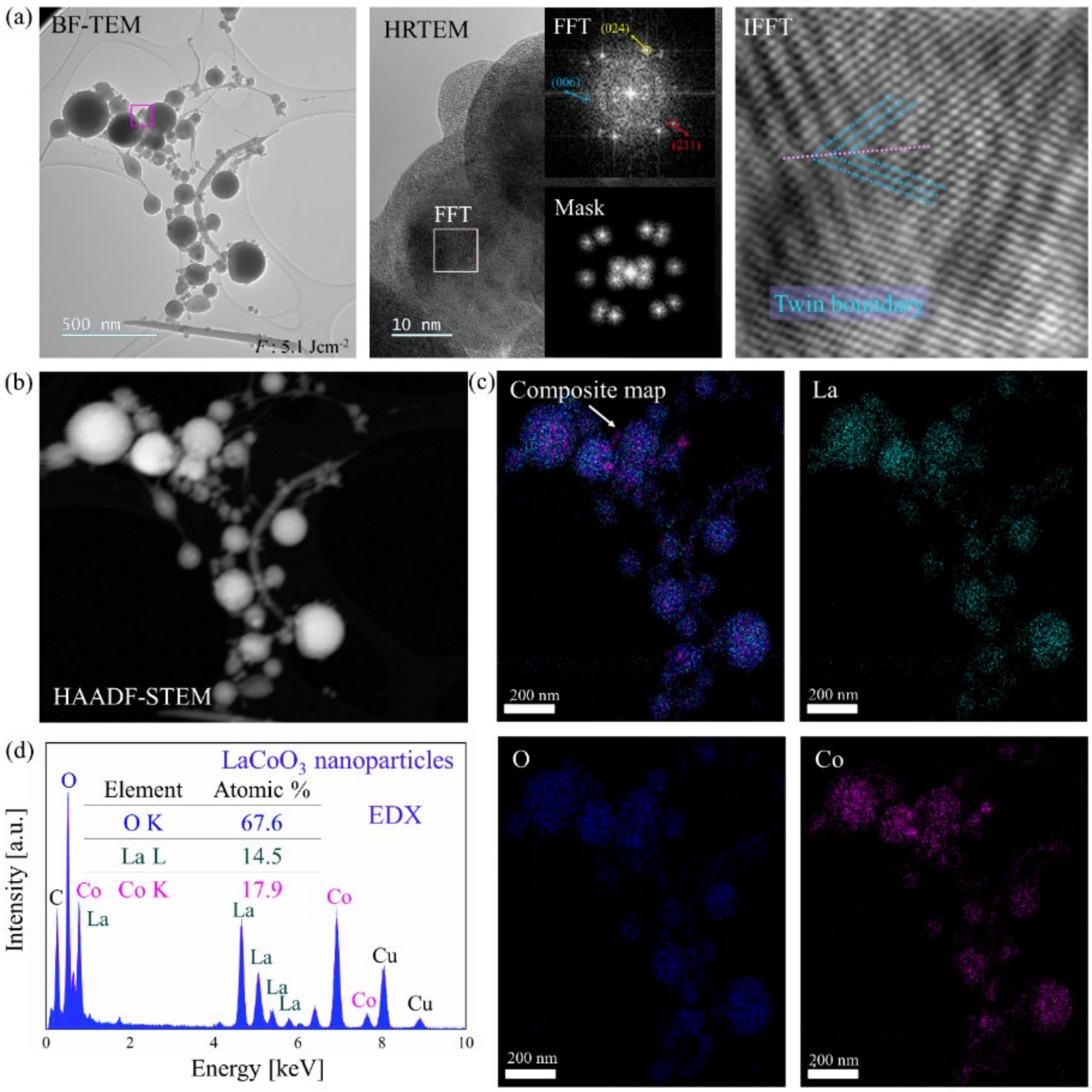

Figure S13. Electron microscopy and EDX mapping analysis of $LaCoO_3$ perovskite nanoparticles femtosecond laser-produced (*F*: 5.1 J cm^{-2}). (a) BF-TEM, HRTEM with FFT/mask (inset) and IFFT showing single twining; (b) HAADF-STEM image; (c) A composite EDX mapping image showing individual EDX elemental maps for La, Co, and O; (d) EDX spectrum obtained from the BF-TEM area.

Figure S14a shows a BF-TEM image from another distribution of perovskite NPs with specific two areas of interest. The HRTEM image, with a corresponding FFT and mask as an inset, and the resulting IFFT image reveal a single twin boundary. The FFT pattern displays reflections from the (012), (110), and (024) planes correspond to rhombohedral $LaCoO_3$ perovskite. HAADF-STEM, composite map and individual elemental maps demonstrate a distribution of uniform NPs and core-shell structures. Smaller NPs similar to those produced at 5.8 J cm^{-2} appears to be more cobalt rich. HRTEM (i) and its IFFT illustrates a single twin boundary. HRTEM (ii) (Figure S14d) along with its magnified view and IFFT image reveals multiple twining. The FFT of this region shows reflection from (110), and (024) planes.

BF-TEM and HAADF-STEM of another region are shown in Figure S15a. The composite map reveals core-shell NPs. The EDX spectrum obtained from BF-TEM, along with its atomic% table as an inset, is shown in Figure S15c.

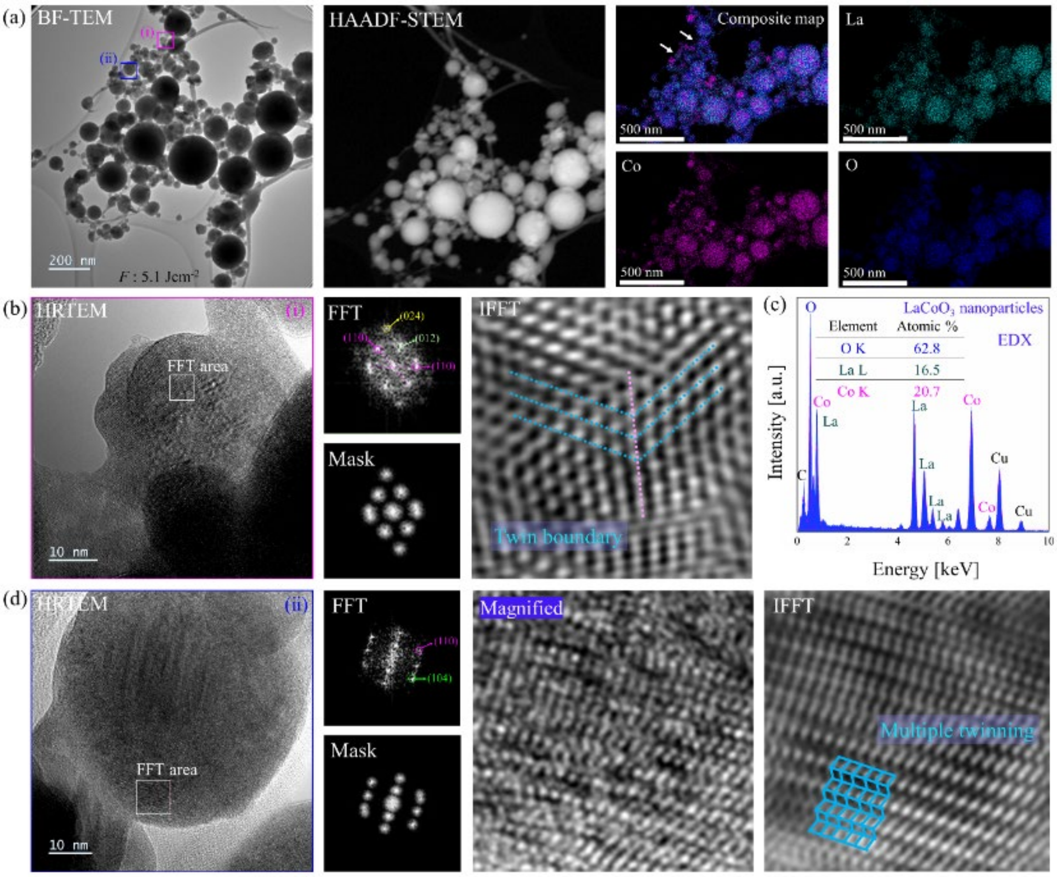

Figure S14. Electron microscopy and EDX mapping analysis of $LaCoO_3$ perovskite nanoparticles femtosecond laser-produced (*F*: 5.1 J cm^{-2}). (a) BF-TEM, HAADF-STEM image and a composite EDX mapping image showing individual EDX elemental maps for La, Co, and O; (b) HRTEM (i), FFT, spot mask, IFFT showing single twinning; (c) EDX spectrum acquired from the BF-TEM area; (d) HRTEM (ii), FFT, spot mask, magnified region of HRTEM and IFFT showing multiple twinning.

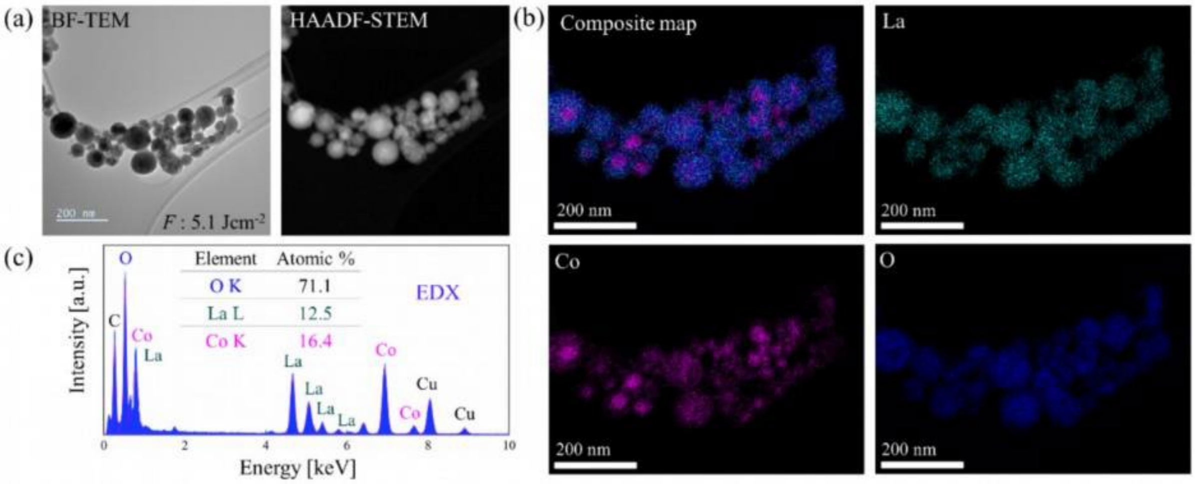

Figure S15. Electron microscopy and EDX mapping analysis of $LaCoO_3$ perovskite nanoparticles femtosecond laser-produced (F: 5.1 J cm^{-2}). (a) BF-TEM and HAADF-STEM image; (b) Composite EDX mapping image indicating individual EDX elemental maps for La, Co, and O; (c) EDX spectrum acquired from the BF-TEM area.

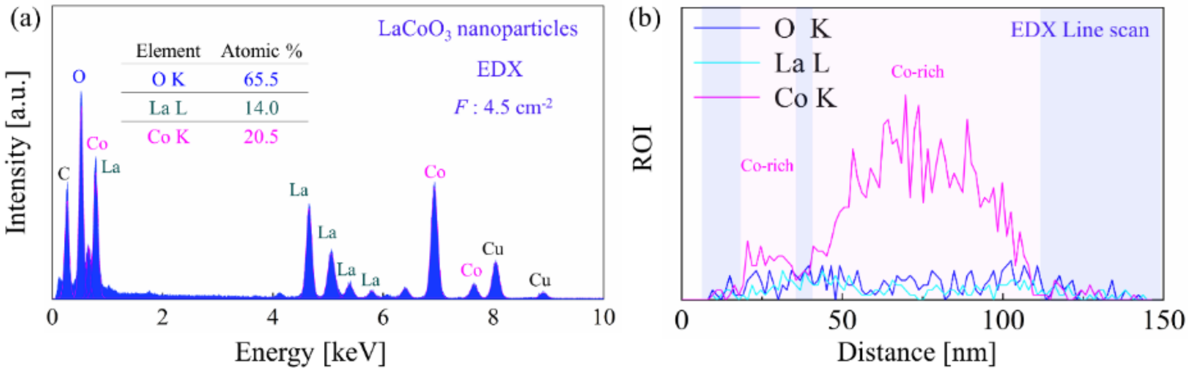

Figure S16. EDX spectrum and corresponding line scan of $LaCoO_3$ perovskite nanoparticles femtosecond laser-produced (F: 4.5 J cm^{-2}). (a) EDX spectrum acquired from the BF-TEM area; (b) line-scan profile obtained from Figure 4d.

Three different distributions of $LaCoO_3$ NPs produced at 4.5 J cm^{-2} are presented in figures S17 to S19. BF-TEM, HAADF-STEM (Figure S17a and S18a) and HADDF-STEM (Figure S19a) shows spherical NPs, and the composite maps in all images show that smaller NPs are cobalt-rich, similar to observations at other applied laser *F*. EDX spectrum acquired from HAADF-STEM area is shown in Figure S17d, S18b and Figure S19b.

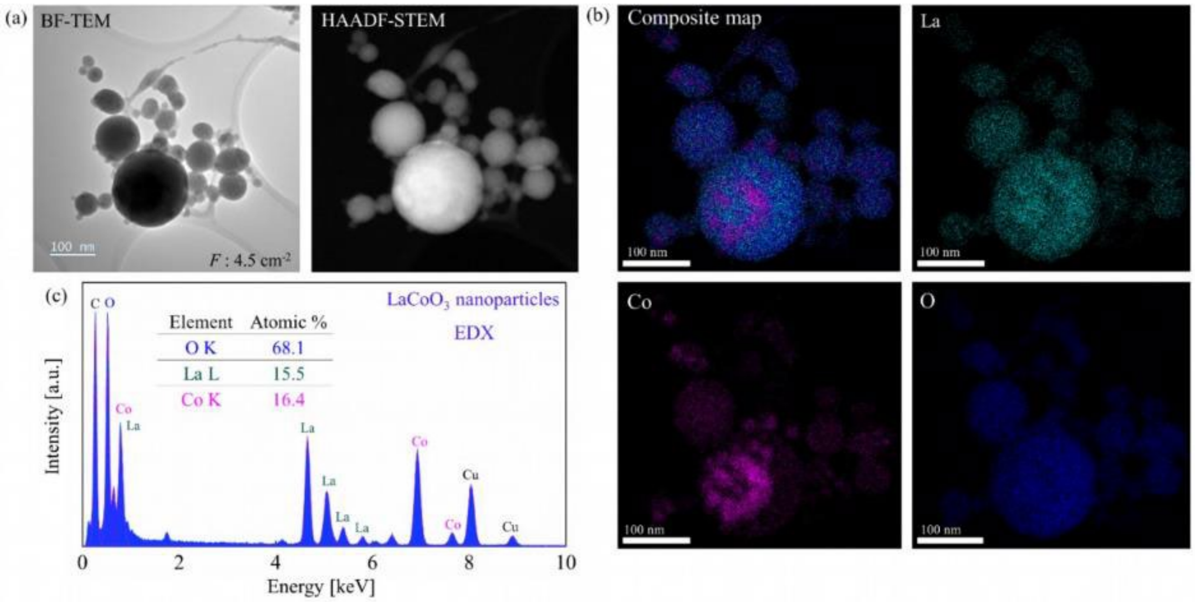

Figure S17. Electron microscopy and EDX mapping analysis of $LaCoO_3$ perovskite nanoparticles femtosecond laser-produced (*F*: 4.5 J cm^{-2}). (a) BF-TEM and HAADF-STEM image; (b) Composite EDX mapping image indicating individual EDX elemental maps for La, Co, and O; (c) EDX spectrum acquired from the BF-TEM area.

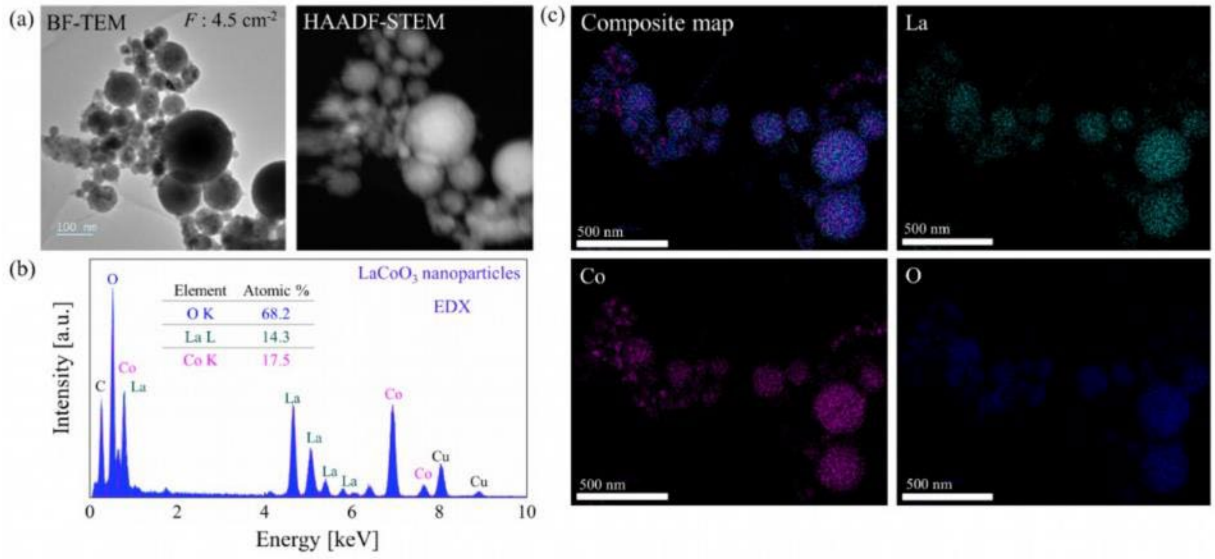

Figure S18. Electron microscopy and EDX mapping analysis of $LaCoO_3$ perovskite nanoparticles femtosecond laser-produced (*F*: 4.5 J cm^{-2}). (a) BF-TEM and HAADF-STEM image; (b) EDX spectrum acquired from the BF-TEM area. (c) Composite EDX mapping image indicating individual EDX elemental maps for La, Co, and O.

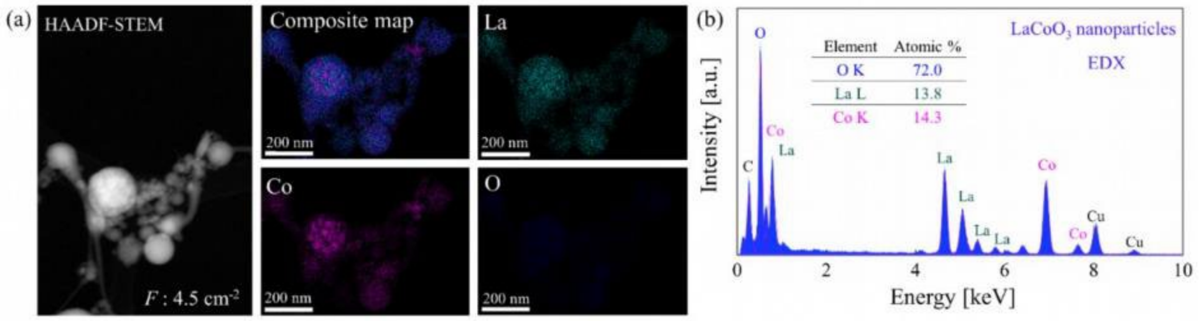

Figure S19. Electron microscopy and EDX mapping analysis of $LaCoO_3$ perovskite nanoparticles femtosecond laser-produced (*F*: 4.5 J cm^{-2}). (a) HAADF-STEM image along with Composite EDX mapping image indicating individual EDX elemental maps for La, Co, and O; (b) EDX spectrum obtained from the HAADF-STEM area.

The laser F was decreased to 3.8 J cm^{-2} to study both crystallinity and defect availability. Identifying a distribution of defective structures was challenging due to the low frequency of such structures. Since the focus is on the analysis of defective NPs, distributions containing defects are studied.

BF-TEM in Figure S20 also shows spherical $LaCoO_3$ NPs. Two areas were chosen for further analysis. HAADF-STEM imaging and composite maps with individual maps for La, Co, and O are shown. A mixture of core-shell and homogeneous distributions is observed. HRTEM (i) and IFFT (Figure S20b) show a tilted grain boundary. HRTEM (ii) and its IFFT display a single twin boundary. The FFT reflects the (104) and (110) planes, corresponding to a rhombohedral perovskite NP. The EDX spectrum obtained from the BF-TEM area, with its atomic% elemental composition shown as an inset, is presented in Figure S20c. Figure S20d shows the NP distribution of another region along with its HAADF-STEM image. HRTEM and its corresponding IFFT display a single twin boundary. The FFT reflects the (012), (006), (024), and (232) planes. The EDX spectrum and composite map show a higher Co contribution in this distribution, and core-shell structures are visible. A spot mask was applied to both distributions. Figure S21 shows a line scan profile from Figure S20d. This profile indicates the possibility of a high amount of Co in some areas.

A different area of the distribution is selected for further analysis. HRTEM (Figure S22a) image and its corresponding IFFT show various atomic orientations and grain boundaries. FFT reflects (110) and (104) planes. The composite map (Figure S22b) reveals that approximately half of this distribution exhibits stoichiometric $LaCoO_3$. The other half, consisting of smaller NPs similar to those observed at other F, is cobalt-rich. The EDX spectrum (Figure S22c) obtained from BF-TEM also indicates stoichiometric $LaCoO_3$.

Figures S23 and S24 also illustrate further distributions of perovskite NPs, notably those that are cobalt-rich as it shown in composite map Figure S23b and S24b and their corresponding EDX spectra in Figures S23c and 24c.

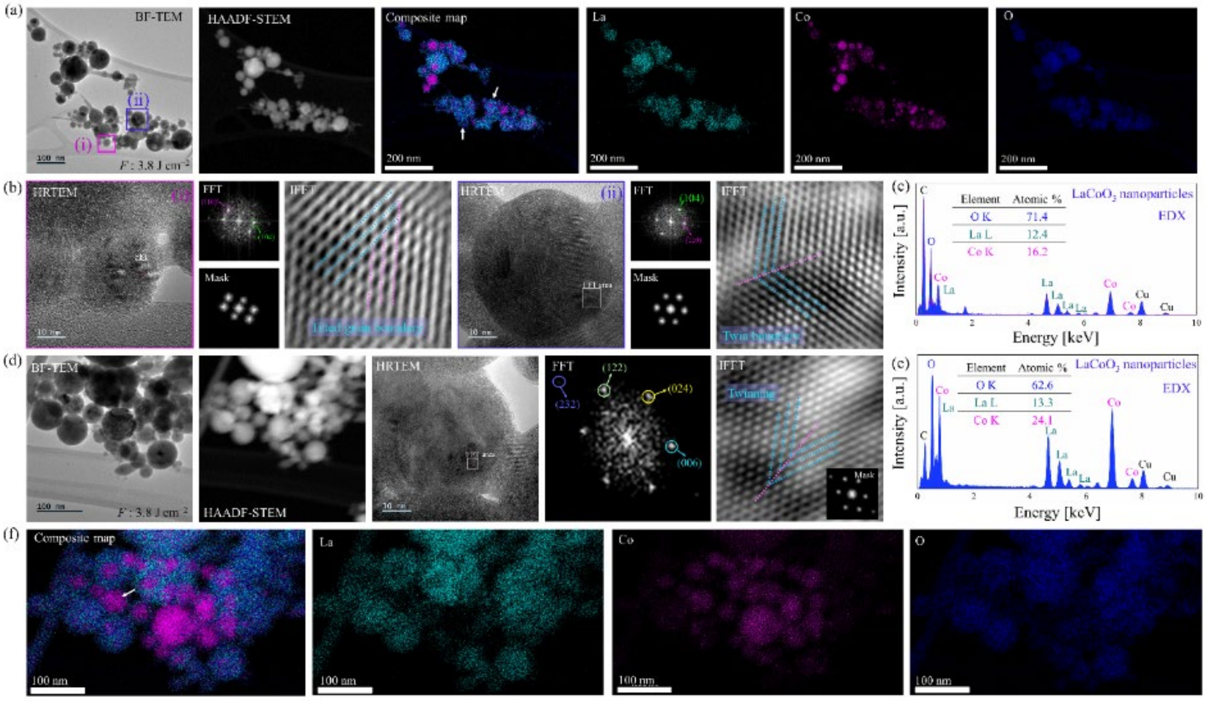

Figure S20. Electron microscopy and EDX mapping analysis of $LaCoO_3$ perovskite nanoparticles femtosecond laser-produced (*F*: 3.8 J cm^{-2}). (a) BF-TEM, HAADF-STEM, and composite EDX mapping image indicating individual EDX elemental maps for La, Co, and O; (b) HRTEM (i), FFT/mask and its corresponding IFFT show titled grain boundary. HRTEM (ii), FFT, spot mask and IFFT show single twin boundary; (c) EDX spectrum acquired from the BF-TEM area; (d) BF-TEM from another region, HAADF-STEM, HRTEM, FFT. IFFT shows also single twining; (e) EDX spectrum acquired from the BF-TEM area; (f) Composite EDX mapping image indicating individual EDX elemental maps for La, Co, and O.

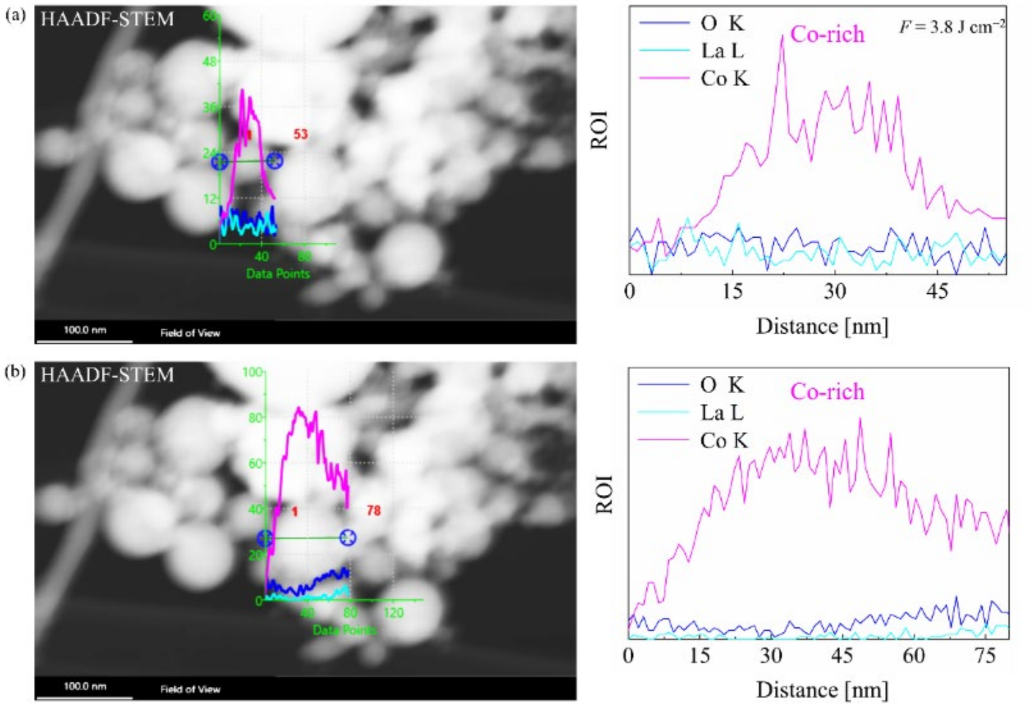

Figure S21. HAADF-STEM images and corresponding line scan profiles of $LaCoO_3$ perovskite nanoparticles femtosecond laser-produced (F: 3.8 J cm^{-2}).

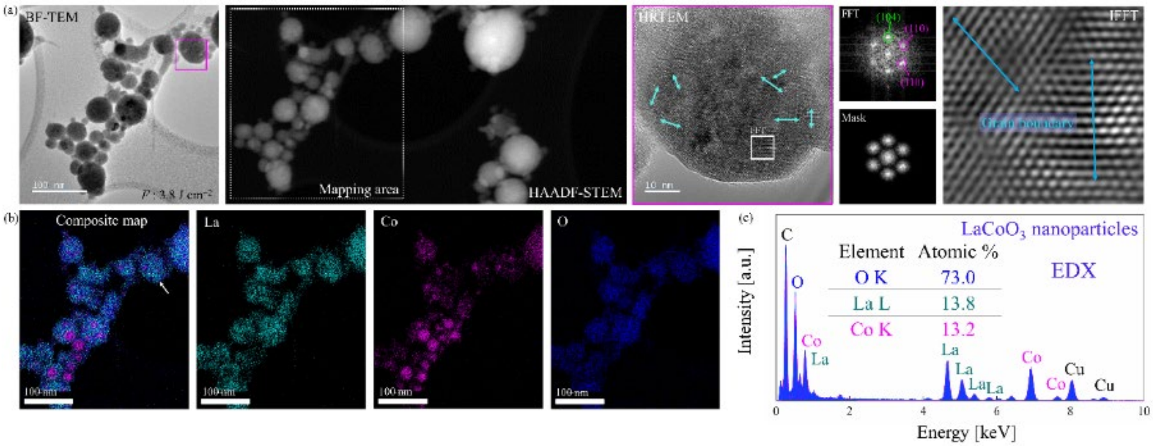

Figure S22. Electron microscopy and EDX mapping analysis of $LaCoO_3$ perovskite nanoparticles femtosecond laser-produced (F: 3.8 J cm^{-2}); (a) BF-TEM, HAADF-STEM, HRTEM, FFT, spot mask and IFFT all illustrate grain boundaries; (b) Composite EDX mapping image indicating individual EDX elemental maps for La, Co, and O; (c) EDX spectrum acquired from the BF-TEM area.

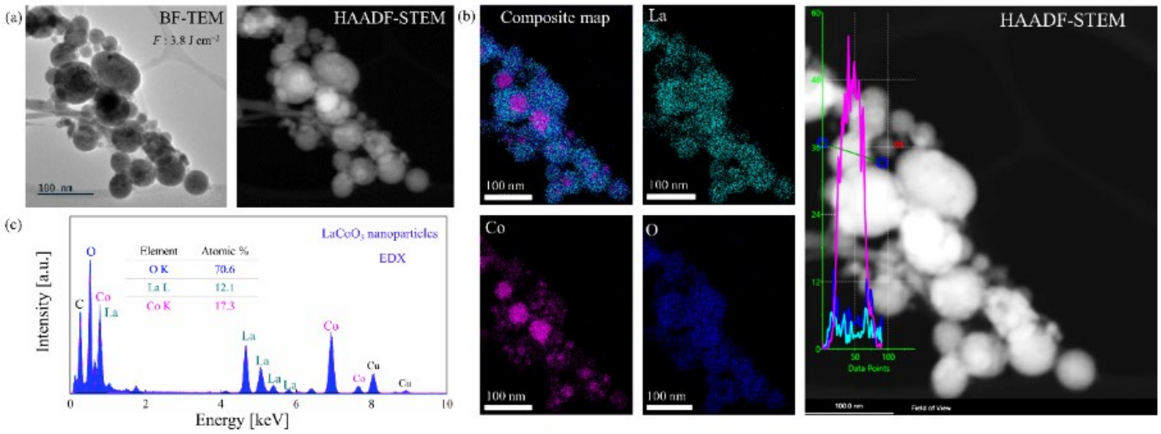

Figure S23. Electron microscopy and EDX mapping analysis of $LaCoO_3$ perovskite nanoparticles femtosecond laser-produced (*F*: 3.8 J cm^{-2}); (a) BF-TEM and HAADF-STEM images; (b) Composite EDX mapping image indicating individual EDX elemental maps for La, Co, and O, along with HAADF-STEM image shows line scan profile; (c) EDX spectrum corresponds to the BF-TEM area.

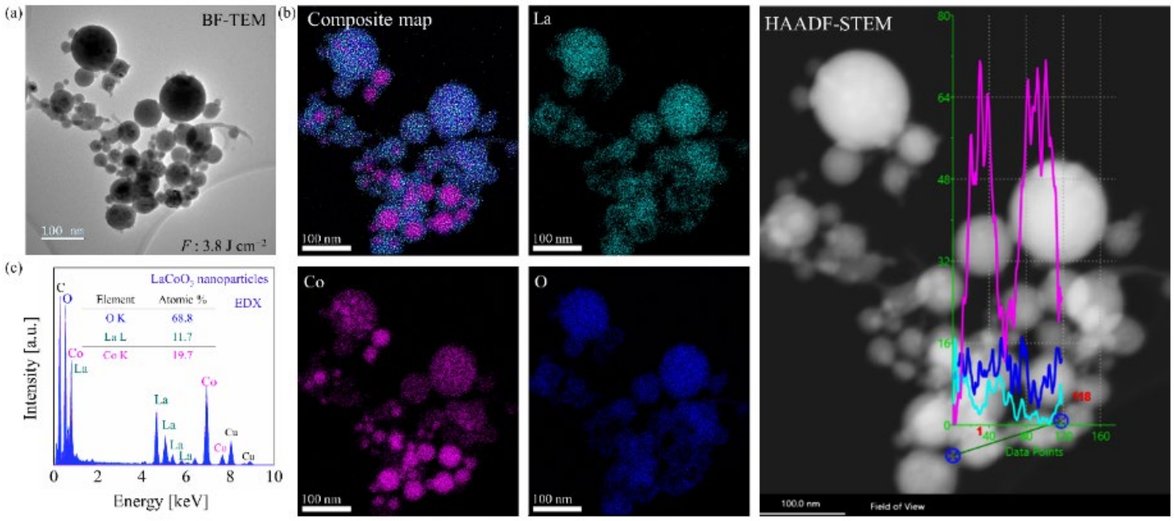

Figure S24. Electron microscopy and EDX mapping analysis of $LaCoO_3$ perovskite nanoparticles femtosecond laser-produced (*F*: 3.8 J cm^{-2}); (a) BF-TEM; (b) Composite EDX mapping image indicating individual EDX elemental maps for La, Co, and O, along with HAADF-STEM image shows line scan profile; (c) EDX spectrum corresponds to the BF-TEM area.

The lowest laser fluence used for the perovskite NPs was 3.1 J cm^{-2}. Therefore, electron microscopy analysis was applied, as before, to study the nature and probability of defects. BF-TEM and HAADF-STEM images, along with EDX elemental maps for La, Co, and O, are shown in Figure S25a. HRTEM (i) and its corresponding IFFT (Figure S25b) show a single twin boundary and multiple twin boundaries coexisting closely. The FFT shows reflections from the (110) and (104) planes. HRTEM (ii) and IFFT show twinning. Its FFT reflects reflections from the (012), (110), (104), and (202) planes. The EDX line scan profile (Figure S25c) obtained from the area shown in Figure S25a further reveals a cobalt-rich region. The corresponding EDX spectrum is presented in Figure S25d.

Figure S26a shows BF-TEM and HAADF-STEM images from another region. HRTEM and its IFFT display a single twin boundary. The FFT shows reflections from the (006) and (024) planes. The composite map (Figure S25d) shows a combination of uniform and core-shell perovskite NP distributions. The EDX average spectrum acquired from the BF-TEM area (Figure S26c) also confirms the stoichiometry. Similar to NPs produced at other laser *F*, the line scan profile (Figure S26e) from the HRTEM area (Figure S26b) shows a combination of stoichiometric $LaCoO_3$ and a cobalt-rich area.

Figure S27a also shows another distribution of NPs, with a stoichiometric NP distribution evident from both EDX spectrum (Figure S27b) and composite elemental mapping image (Figure S27c). Figure S28 also shows a stoichiometric distribution of NPs. Similar to other regions, Figure S29 also shows a mostly uniform distribution of La, Co, and O, which should correspond to stoichiometric $LaCoO_3$ NPs.

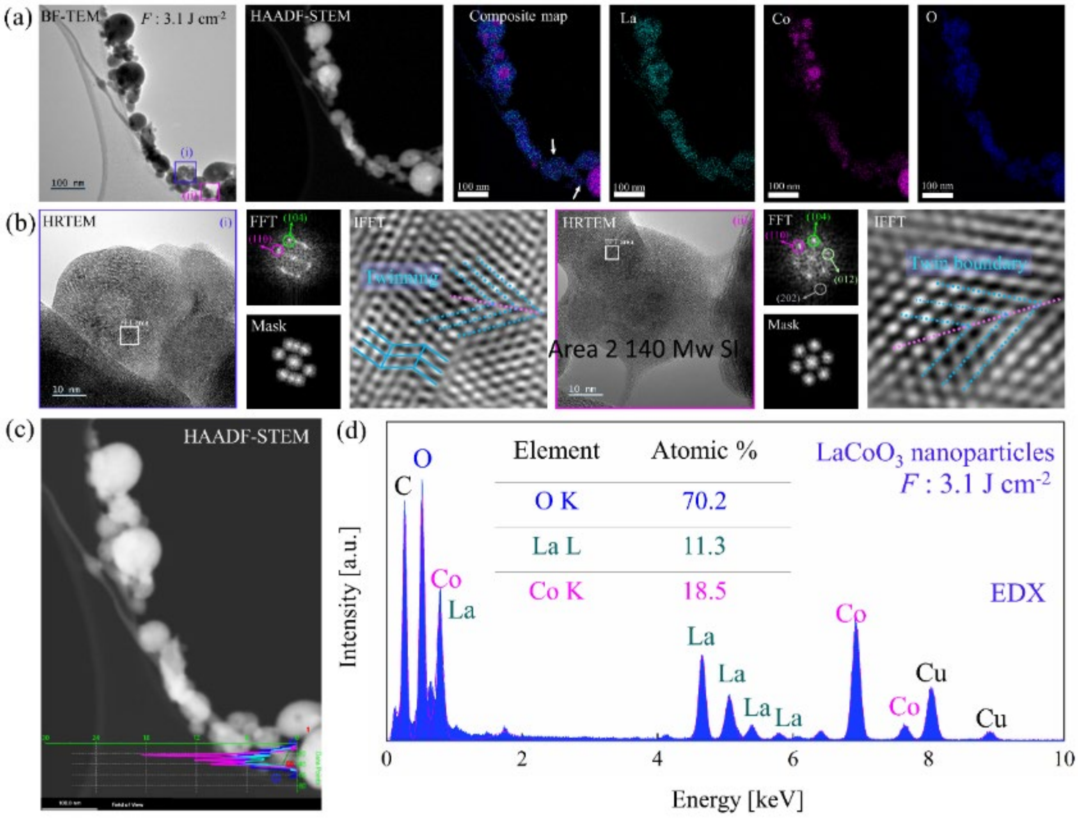

Figure S25. Electron microscopy and EDX mapping analysis of $LaCoO_3$ perovskite nanoparticles femtosecond laser-produced (*F*: 3.1 J cm^{-2}). (a) BF-TEM, HAADF-STEM and a composite EDX mapping image and its corresponding individual EDX elemental maps for La, Co and O; (b) HRTEM (i) FFT, spot mask and IFFT showing single and multiple twinning; another HRTEM (ii) with corresponding FFT/mask and its FFT, showing a single twin boundary; (c) HAADF-STEM image and its corresponding line scan profile; (d) EDX spectrum obtained from BF-TEM area.

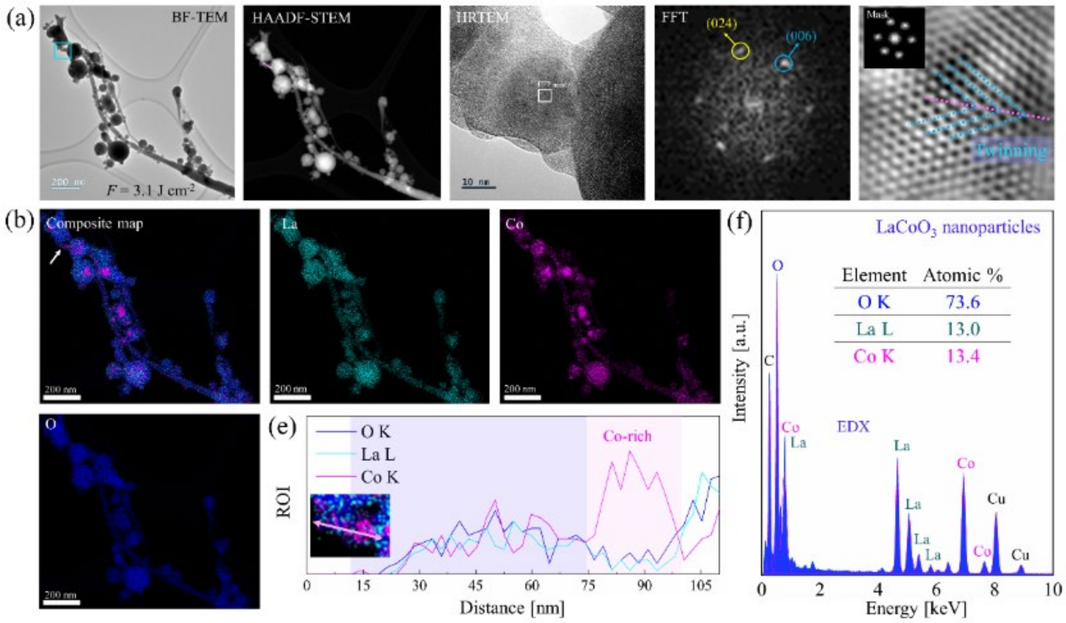

Figure S26. Electron microscopy and EDX mapping analysis of $LaCoO_3$ perovskite nanoparticles femtosecond laser-produced (*F*: 3.1 J cm^{-2}). (a) BF-TEM image, HAADF-STEM, HRTEM, FFT, IFFT and mask as inset. HRTEM also shows a single twin boundary; (b) Composite EDX mapping image indicating individual elemental maps for La, Co and O; (c) EDX spectrum obtained from the BF-TEM area; (d) Line scan profile form HRTEM area.

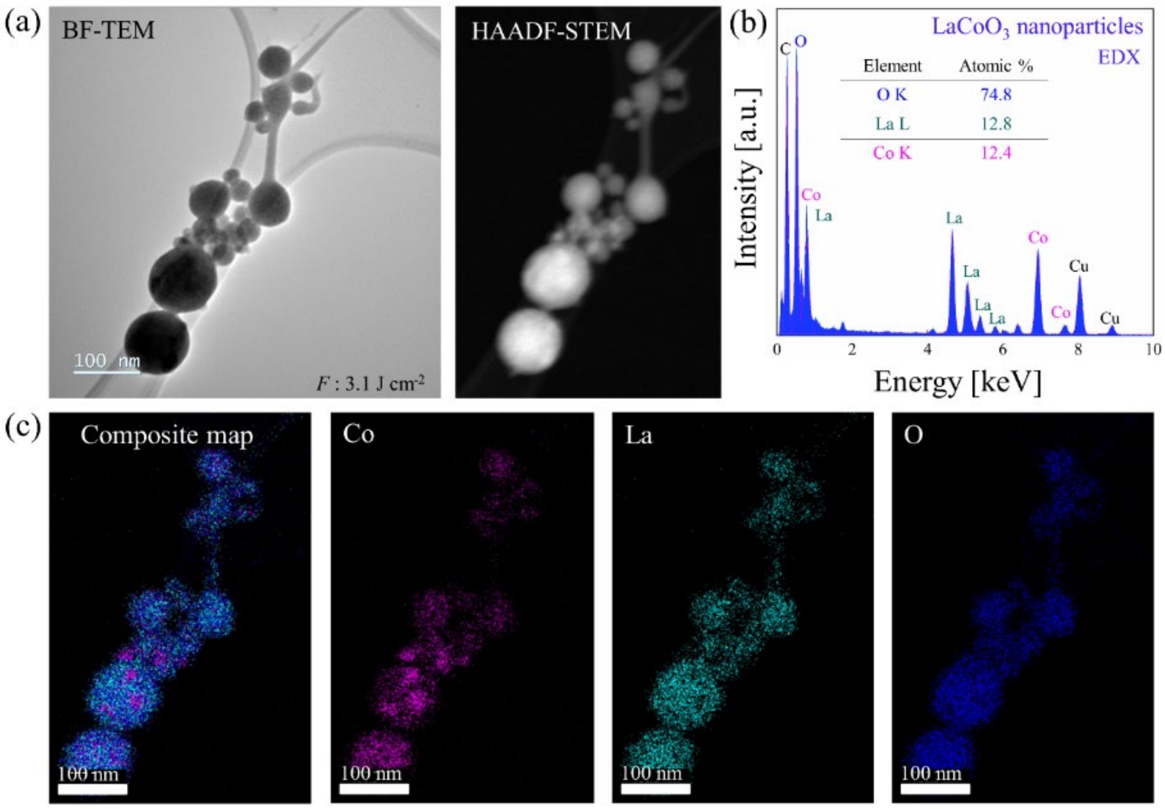

Figure S27. Electron microscopy analysis of $LaCoO_3$ perovskite nanoparticles femtosecond laser-produced (*F*: 3.1 J cm^{-2}). (a) BF-TEM and HAADF-STEM; (b) EDX spectrum obtained from the BF-TEM; (c) EDX composite map with individual elemental maps (La, Co and O).

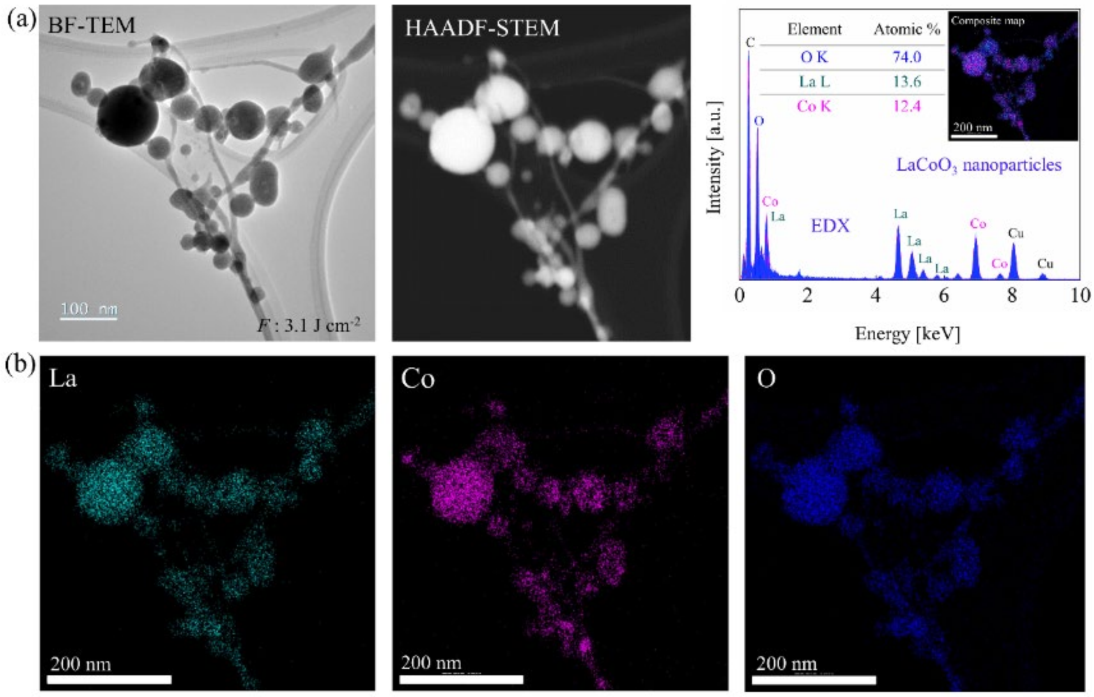

Figure S28. Electron microscopy analysis of $LaCoO_3$ perovskite nanoparticles femtosecond laser-produced (*F*: 3.1 J cm^{-2}). (a) BF-TEM, HAADF-STEM and EDX spectrum with composite map (inset); (b) Individual elemental maps for La, Co and O.

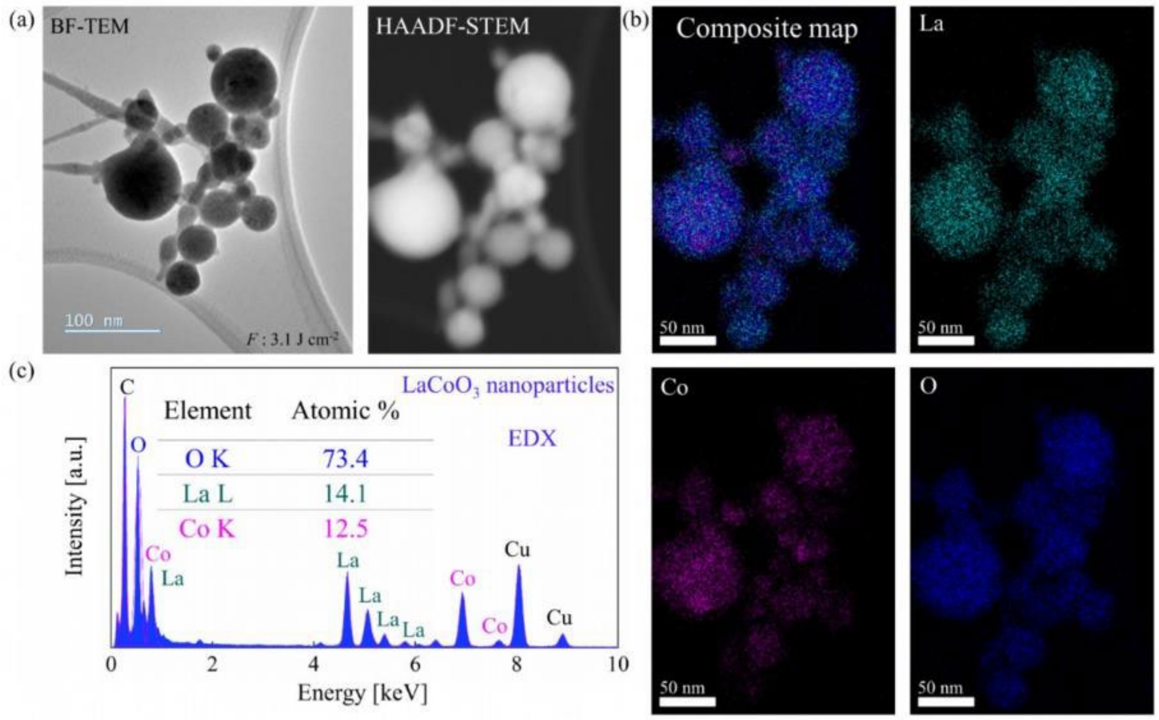

Figure S29. Electron microscopy analysis of $LaCoO_3$ perovskite nanoparticles femtosecond laser-produced (*F*: 3.1 J cm^{-2}). (a) BF-TEM and HAADF-STEM images; (b) EDX composite map with individual elemental maps (La, Co and O); (c) EDX spectrum obtained from the BF-TEM area.

Table S4. Summary of atomic% ratio of Co/La in various *F* obtained from EDX analysis.

F [J cm^{-2}]	Ratio Co/La %
3.1	1.06
3.8	1.42
4.5	1.19
5.1	1.24
5.8	1.11

4.2. Defect density calculation

To accurately characterize the level of structural disorder, the total number of distinct defects within individual nanoparticles was first quantified. Thus, HRTEM images were used to count all visible structural defects, including twinning, dislocations, and grain boundaries within each NP individually. For example, a single NP exhibiting one twin boundary, one dislocation, and one grain boundary was recorded as having three total defects.

Next, we calculated the average number of defects (no SI unit) and determined the average size of the defective nanoparticles (d in nm). Finally, the surface defect concentration ($\rho_{surface\ defect}$ in nm^{-2}) was calculated by dividing the average defect number by the average surface area of the defective nanoparticles.

d is the average NP diameter containing defects [nm]

$A = \pi d^2$ is the surface area assuming spherical NPs [nm^2]

Note:

Example for NPs synthesized at F : 5.8 J cm^{-2}

Average defect number : 1.91 [no SI unit]

Average d for NPs for which defects were visible = 30.5 nm

$\rho_{surface\ defect} =$ 1.91/2920.98 [nm^2] = 6.5×10^{-4} [nm^{-2}]

4.3. Surface area and volume distribution

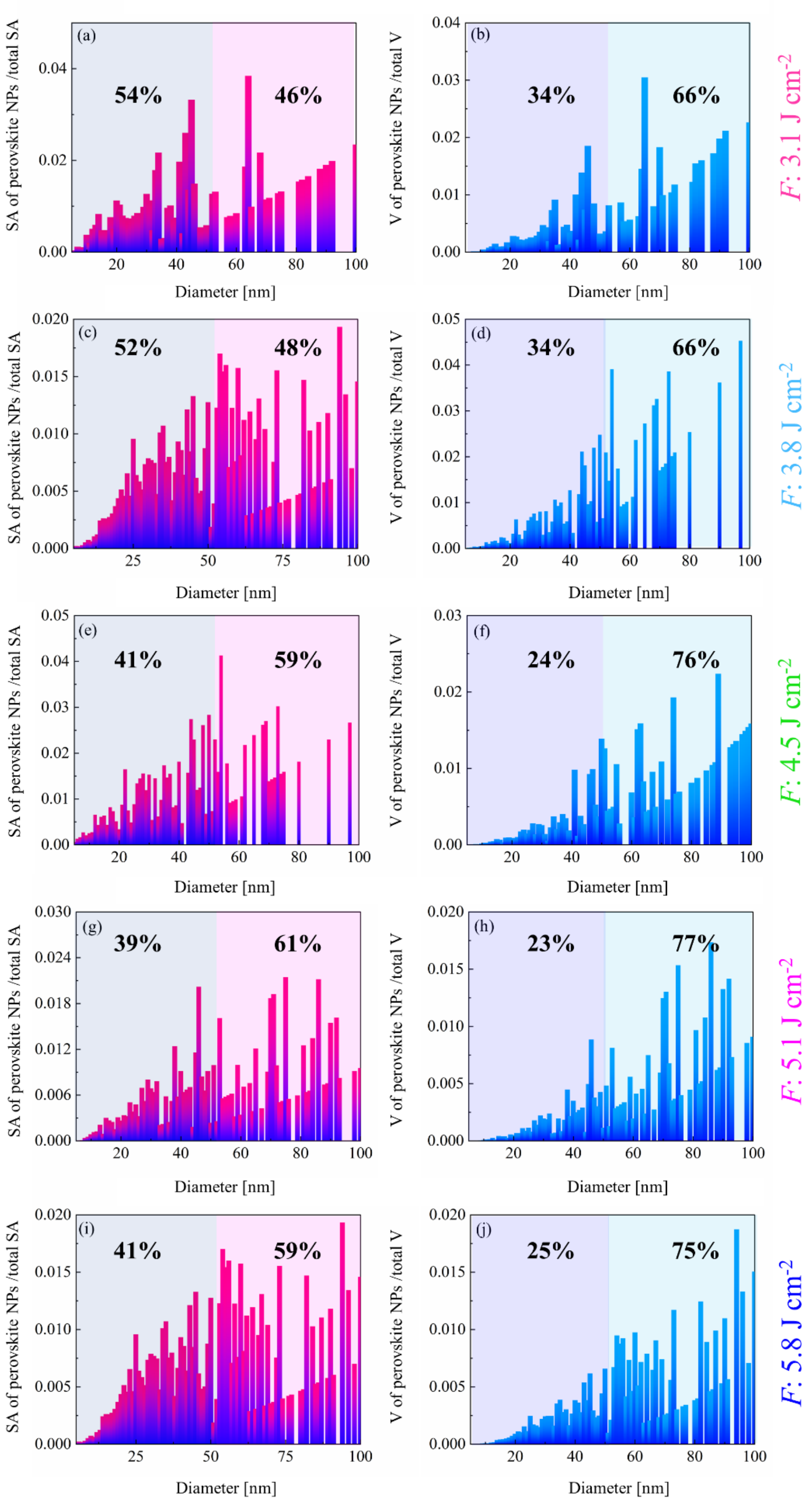

Figure S30. Surface area distribution (a, c, e, g, i) and volume distribution (b, d, f, h, j) of $LaCoO_3$ perovskite nanoparticles femtosecond laser-produced at fluences of 3.1, 3.8, 4.5, 5.1, and 5.8 J cm^{-2}.

Table S5. Summary of the number-weighted size distribution of $LaCoO_3$ perovskite nanoparticles femtosecond laser-produced at *F*: 3.1 J cm^{-2}.

Diameter [nm]	Counts	$SA/SA_{total\%}$	SUM	$V/V_{total\%}$	SUM
5	4	0.02		V%	
6	4	0.03		0.00	
7	10	0.12		0.00	
8	7	0.11		0.01	
9	5	0.10		0.01	
10	16	0.38		0.01	
11	7	0.20		0.05	
12	15	0.51		0.03	
13	15	0.59		0.08	
14	18	0.83		0.10	
15	9	0.48		0.14	
16	8	0.48		0.09	
17	7	0.47		0.10	
18	10	0.76		0.10	
19	6	0.51		0.17	
20	12	1.13		0.12	
21	10	1.04		0.28	
22	7	0.80		0.27	
23	6	0.74		0.22	
24	5	0.68		0.21	
25	5	0.73		0.20	
26	5	0.79		0.23	
27	5	0.86		0.26	
29	5	0.99		0.29	
30	6	1.27		0.35	
31	5	1.13		0.47	
32	2	0.48		0.43	
33	7	1.79		0.19	
34	8	2.17		0.73	
35	1	0.29		0.91	
36	1	0.30		0.12	
37	3	0.96		0.14	
38	3	1.02		0.44	
39	2	0.71		0.48	
40	2	0.75		0.34	
41	5	1.97		0.37	
42	1	0.41		1.00	
43	6	2.60		0.22	
44	3	1.36		1.39	
45	7	3.33		0.74	
46	3	1.49		1.85	
48	1	0.54		0.85	
50	1	0.59	**54%**	0.32	**34%**
52	2	1.27		0.36	
53	2	1.32		0.82	
57	1	0.76		0.87	
58	1	0.79		0.54	

60	1	0.84		0.57	
63	2	1.86		0.63	
64	4	3.85		1.45	
65	1	0.99		3.05	
68	2	2.17		0.80	
70	1	1.15		1.83	
71	1	1.18		1.00	
74	1	1.29		1.04	
75	1	1.32		1.18	
81	1	1.54		1.23	
82	1	1.58		1.54	
84	1	1.66		1.60	
88	1	1.82		1.72	
90	1	1.90		1.98	
92	1	1.99		2.12	
100	1	2.35	46%	2.26	66%
	295		100		100

Table S6. Summary of the number-weighted size distribution of $LaCoO_3$ perovskite nanoparticles femtosecond laser-produced at *F*: 3.8 J cm^{-2}.

Diameter [nm]	Counts	$SA/SA_{total\%}$	SUM	$V/V_{total\%}$	SUM
5	9	0.06		0.01	
6	13	0.13		0.01	
7	12	0.17		0.02	
8	15	0.27		0.04	
9	9	0.21		0.03	
10	9	0.26		0.04	
11	8	0.27		0.05	
12	16	0.65		0.14	
13	9	0.43		0.10	
14	11	0.61		0.15	
15	10	0.64		0.17	
16	6	0.44		0.12	
17	10	0.82		0.24	
18	8	0.73		0.23	
19	5	0.51		0.17	
20	3	0.34		0.12	
21	7	0.87		0.32	
22	12	1.65		0.63	
23	5	0.75		0.30	
24	3	0.49		0.21	
25	5	0.89		0.39	
26	7	1.34		0.61	
27	7	1.45		0.68	
28	7	1.55		0.76	
29	5	1.19		0.61	
30	6	1.53		0.80	
31	2	0.54		0.30	
32	5	1.45		0.81	
33	2	0.62		0.36	
34	3	0.98		0.59	
35	5	1.74		1.06	
36	4	1.47		0.93	
37	4	1.55		1.01	
38	2	0.82		0.54	
39	2	0.86		0.59	
40	4	1.81		1.27	
41	1	0.48		0.34	
43	3	1.57		1.18	
44	5	2.74		2.11	
45	4	2.30		1.81	
46	2	1.20		0.97	
47	2	1.25		1.03	
48	4	2.61		2.20	
49	1	0.68		0.58	
50	4	2.83	52%	2.48	34%

51	1	0.74		0.66	
52	3	2.30		2.09	
53	2	1.59		1.48	
54	5	4.13		3.91	
56	2	1.78		1.74	
58	1	0.95		0.97	
57	1	0.92		0.92	
59	1	0.99		1.02	
61	1	1.05		1.13	
62	2	2.18		2.37	
65	2	2.39		2.73	
68	2	2.62		3.12	
69	2	2.70		3.26	
70	1	1.39		1.70	
71	1	1.43		1.78	
72	1	1.47		1.85	
73	2	3.02		3.86	
74	1	1.55		2.01	
75	1	1.59		2.09	
80	1	1.81		2.54	
90	1	2.30		3.62	
97	1	2.67		4.53	
100	1	3.06	48%	5.58	66%
	312		100		100

Table S7. Summary of the number-weighted size distribution of $LaCoO_3$ perovskite nanoparticles femtosecond laser-produced at *F*: 4.5 J cm^{-2}.

Diameter [nm]	Counts	$SA/SA_{total\%}$	SUM	$V/V_{total\%}$	SUM
4	2	0.005		0.000	
5	7	0.026		0.001	
6	4	0.021		0.001	
7	6	0.043		0.003	
8	12	0.112		0.010	
9	6	0.071		0.007	
10	11	0.161		0.017	
11	12	0.212		0.025	
12	9	0.189		0.025	
13	12	0.296		0.042	
14	11	0.315		0.048	
15	14	0.460		0.075	
16	10	0.374		0.065	
17	4	0.169		0.031	
18	10	0.473		0.093	
19	9	0.475		0.098	
20	7	0.409		0.089	
21	6	0.387		0.088	
22	6	0.424		0.101	
23	9	0.696		0.174	
24	9	0.757		0.198	
25	6	0.548		0.149	
26	7	0.691		0.195	
27	9	0.959		0.281	
28	4	0.458		0.139	
29	7	0.860		0.271	
30	6	0.789		0.257	
31	3	0.421		0.142	
32	1	0.150		0.052	
33	4	0.637		0.228	
34	6	1.014		0.374	
35	3	0.537		0.204	
36	4	0.757		0.296	
37	5	1.000		0.402	
38	4	0.844		0.348	
39	2	0.445		0.188	
41	9	2.211		0.985	
42	1	0.258		0.118	
43	3	0.811		0.379	
44	2	0.566		0.270	
46	6	1.855		0.927	
47	6	1.937		0.989	
48	3	1.010		0.527	
49	2	0.702		0.374	
50	7	2.557	**41%**	1.389	**24%**
51	6	2.280		1.264	

52	2	0.790		0.446	
53	2	0.821		0.473	
54	2	0.852		0.500	
55	4	1.768		1.056	
56	1	0.458		0.279	
60	2	1.052		0.686	
61	1	0.544		0.360	
62	4	2.247		1.513	
63	4	2.320		1.588	
64	2	1.197		0.832	
65	1	0.617		0.436	
66	1	0.637		0.456	
67	2	1.312		0.955	
68	1	0.676		0.499	
70	2	1.432		1.089	
72	1	0.757		0.593	
74	3	2.400		1.930	
75	1	0.822		0.670	
76	1	0.844		0.697	
80	1	0.935		0.813	
82	1	0.983		0.875	
85	1	1.056		0.975	
87	1	1.106		1.045	
88	1	1.132		1.082	
89	2	2.315		2.238	
93	1	1.264		1.277	
94	1	1.291		1.319	
95	1	1.319		1.361	
97	1	1.375		1.449	
98	1	1.403		1.494	
99	1	1.432		1.540	
100	1	1.461	**59%**	1.588	**76%**
	343		100		100

Table S8. Summary of the number-weighted size distribution of $LaCoO_3$ perovskite nanoparticles femtosecond laser-produced at *F*: 5.1 J cm^{-2}.

Diameter [nm]	Counts	$SA/SA_{total\%}$	SUM	$V/V_{total\%}$	SUM
5	2	0.005		0.000	
6	2	0.007		0.000	
7	2	0.009		0.001	
8	6	0.037		0.003	
9	7	0.054		0.005	
10	9	0.086		0.008	
11	10	0.115		0.012	
12	9	0.124		0.014	
13	13	0.210		0.026	
14	5	0.094		0.012	
15	14	0.301		0.043	
16	11	0.269		0.041	
17	9	0.248		0.040	
18	5	0.155		0.027	
19	9	0.310		0.056	
20	7	0.267		0.051	
21	8	0.337		0.067	
22	7	0.323		0.068	
23	10	0.505		0.111	
24	7	0.385		0.088	
25	8	0.477		0.114	
26	5	0.323		0.080	
27	10	0.696		0.179	
28	7	0.524		0.140	
29	10	0.803		0.222	
30	8	0.687		0.197	
31	7	0.642		0.190	
32	8	0.782		0.239	
33	2	0.208		0.065	
34	2	0.221		0.072	
35	5	0.584		0.195	
36	2	0.247		0.085	
37	4	0.523		0.184	
38	9	1.240		0.449	
39	4	0.581		0.216	
40	6	0.916		0.349	
41	4	0.642		0.251	
42	4	0.673		0.270	
43	4	0.706		0.289	
44	1	0.185		0.078	
45	6	1.159		0.497	
46	10	2.019		0.886	
47	4	0.843		0.378	
48	1	0.220		0.101	
49	4	0.916		0.428	
48	3	0.660		0.302	

50	1	0.239	**39**	0.114	**23%**
51	4	0.993		0.483	
52	1	0.258		0.128	
53	6	1.608		0.813	
54	2	0.557		0.287	
55	2	0.577		0.303	
56	2	0.599		0.320	
57	2	0.620		0.337	
58	1	0.321		0.178	
59	3	0.997		0.561	
60	1	0.344		0.197	
61	2	0.710		0.413	
63	2	0.757		0.455	
64	1	0.391		0.239	
65	3	1.210		0.750	
67	1	0.428		0.274	
69	2	0.909		0.598	
70	4	1.870		1.248	
71	4	1.924		1.303	
72	2	0.989		0.679	
73	1	0.509		0.354	
74	1	0.523		0.369	
75	4	2.147		1.535	
76	1	0.551		0.399	
79	1	0.596		0.449	
81	2	1.252		0.967	
82	1	0.642		0.502	
83	1	0.657		0.520	
84	2	1.347		1.079	
86	3	2.117		1.736	
88	1	0.739		0.620	
89	1	0.756		0.641	
90	2	1.546		1.327	
92	2	1.615		1.417	
93	1	0.825		0.732	
98	1	0.916		0.856	
100	1	0.954	**61%**	0.910	**77%**
	362		100		100

Table S9. Summary of the number-weighted size distribution of $LaCoO_3$perovskite nanoparticles femtosecond laser-produced at *F*: 5.8 J cm^{-2}.

Diameter [nm]	Counts	SA/SA$_{total\%}$	SUM	V/V$_{total\%}$	SUM
6	8	0.02		0.001	
7	5	0.02		0.001	
8	6	0.03		0.002	
9	9	0.05		0.005	
10	10	0.07		0.008	
11	7	0.06		0.007	
12	10	0.11		0.013	
13	10	0.12		0.017	
14	17	0.24		0.035	
15	16	0.26		0.041	
16	14	0.26		0.043	
17	13	0.27		0.048	
18	13	0.31		0.057	
19	14	0.37		0.072	
20	15	0.44		0.090	
21	16	0.51		0.111	
22	13	0.46		0.104	
23	17	0.66		0.156	
24	11	0.46		0.114	
25	21	0.96		0.247	
26	13	0.64		0.172	
27	11	0.58		0.163	
28	9	0.51		0.149	
29	12	0.74		0.220	
30	12	0.79		0.244	
31	11	0.77		0.247	
32	10	0.75		0.247	
33	6	0.48		0.162	
34	12	1.01		0.355	
35	12	1.07		0.387	
36	8	0.76		0.281	
37	8	0.80		0.305	
38	4	0.42		0.165	
39	6	0.67		0.268	
40	8	0.93		0.385	
41	7	0.86		0.363	
42	5	0.64		0.279	
43	9	1.21		0.538	
44	6	0.85		0.385	
45	9	1.33		0.617	
46	4	0.62		0.293	
47	3	0.48		0.234	
48	3	0.50		0.250	
49	5	0.88		0.443	
50	7	1.28	**41%**	0.658	**25%**
51	1	0.19		0.100	

52	2	0.39		0.212	
53	6	1.23		0.672	
54	8	1.70		0.948	
55	7	1.54		0.876	
56	7	1.60		0.925	
57	3	0.71		0.418	
58	5	1.23		0.734	
59	3	0.76		0.464	
60	6	1.58		0.975	
61	3	0.81		0.512	
62	4	1.12		0.717	
63	1	0.29		0.188	
64	4	1.19		0.789	
65	1	0.31		0.207	
66	3	0.95		0.649	
67	4	1.31		0.905	
68	1	0.34		0.237	
69	3	1.04		0.742	
70	1	0.36		0.258	
71	1	0.37		0.269	
72	2	0.76		0.562	
73	4	1.55		1.171	
74	1	0.40		0.305	
76	1	0.42		0.330	
77	1	0.43		0.344	
80	1	0.47		0.385	
81	1	0.48		0.400	
82	3	1.47		1.245	
84	2	1.03		0.892	
85	1	0.53		0.462	
86	1	0.54		0.479	
87	2	1.10		0.991	
89	1	0.58		0.530	
90	2	1.18		1.097	
91	1	0.60		0.567	
94	3	1.93		1.875	
96	2	1.34		1.331	
98	1	0.70		0.708	
100	2	1.46	59%	1.505	75%
	551		100		100

5. Catalysis section

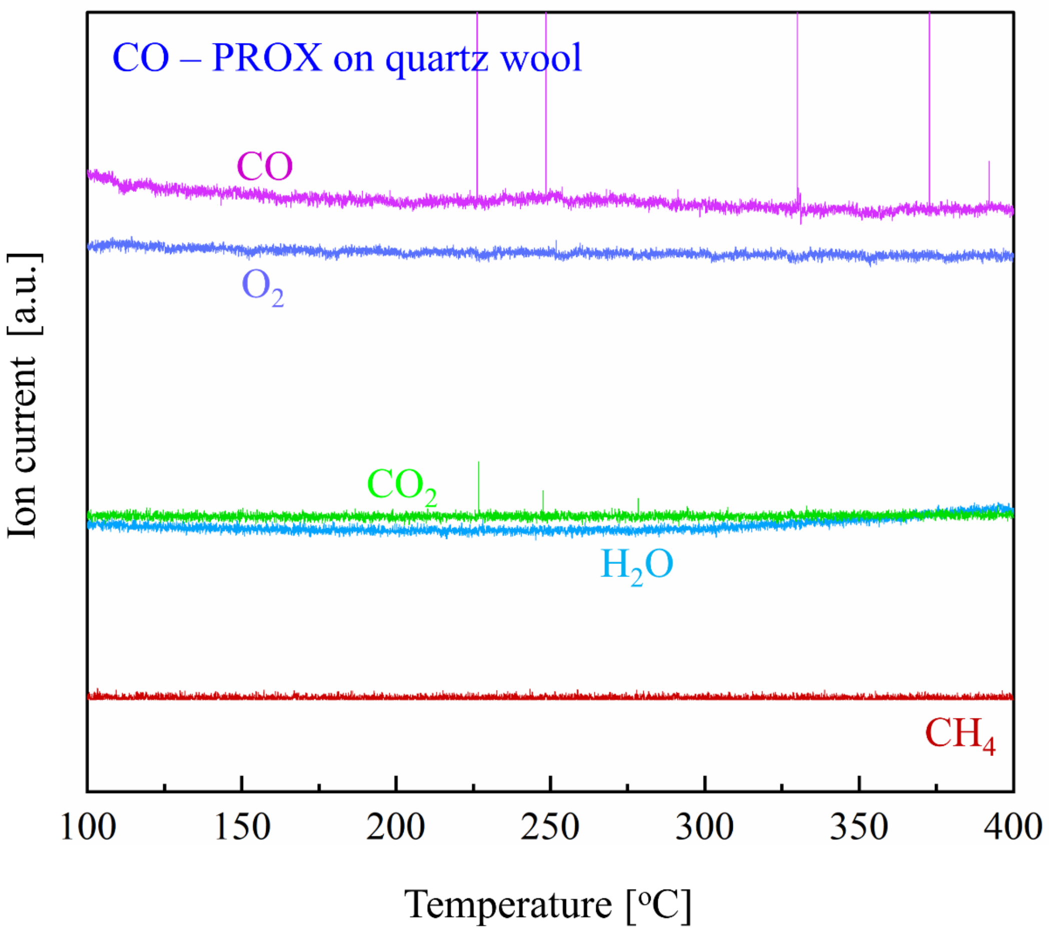

Figure S31. MS analysis of CO-PROX on inert quartz wool.

5.1. Selectivity of CO_2 (S_{CO2})

Selectivity of CO_2 (S_{CO2}) = [CO_2 MS intensity (I_{CO2})/RSF / (CO_2 MS intensity (I_{CO2})/RSF + H_2O MS intensity (I_{H2O})/RSF)] × 100

***Note*:** RSF is relative sensitivity factor; CO_2: 1.05 and H_2O: 1.42

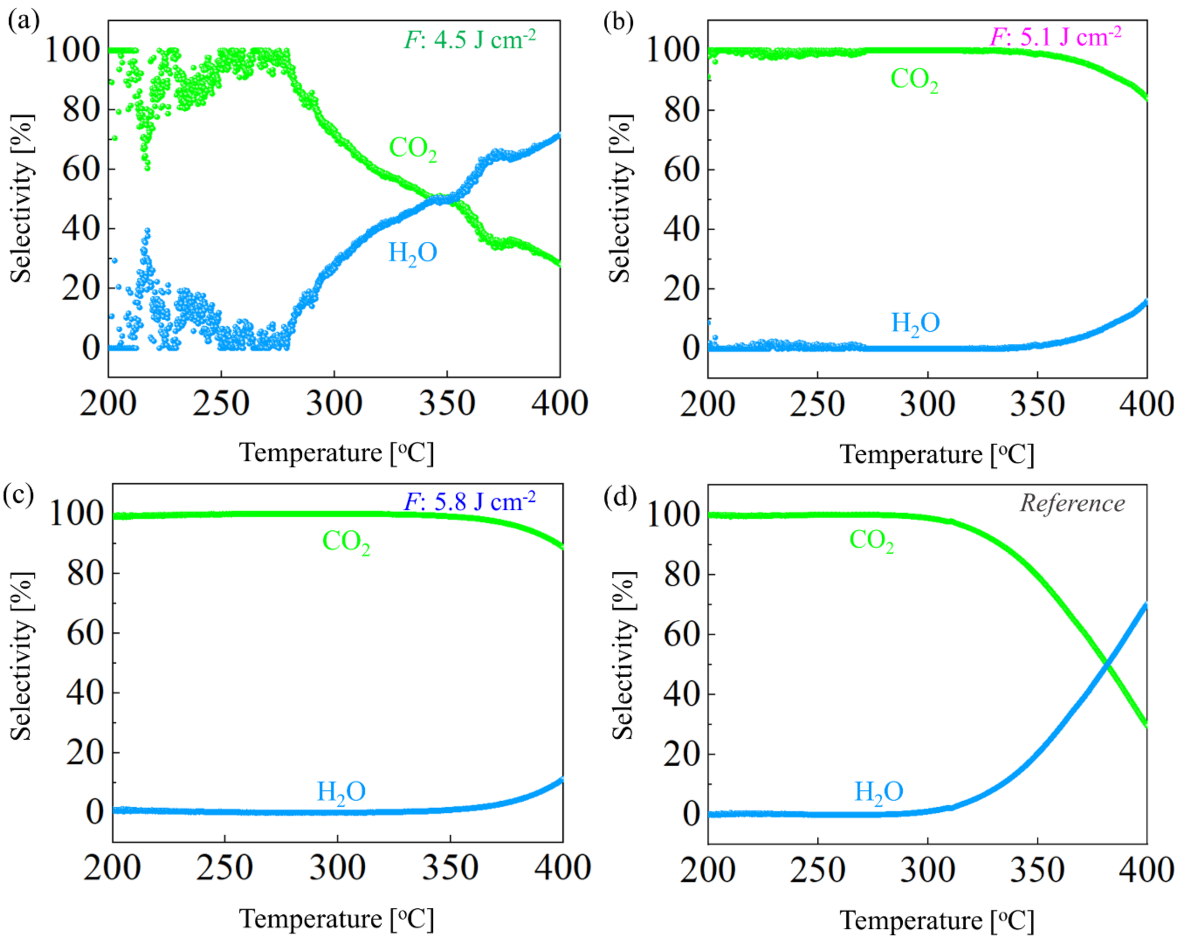

Figure S32. Corrected MS data and their calculated selectivity of $LaCoO_3$ for CO-PROX at different temperatures for CO_2 and H_2O. (a) F : 5.8 J cm^{-2}; (b) F : 5.1 J cm^{-2}; (c) F : 4.5 J cm^{-2}; (d) F : Reference sample J cm^{-2}.

5.2. Specific activity calculation

We illustrate the specific activity calculation for just one sample and temperature here. The other calculations were performed in the same way.

***F*: 5.8 J cm^{-2}, *T*: 400° C, CO conversion: 43.25%**

Total Gas Flow Rate (V): 50 ml/min × 1L/1000 ml × 1 min/ 60 s = 0.0008333 L/s

Moles of total gas entering per second (n_{total}): [P × V/R × T]

R = 0.08206 L atm mol^{-1} K^{-1}

[1 atm × 0.0008333 L/s] / [0.08206 L atm mol^{-1} K^{-1} × 298 K] = 3.407 × 10^{-5} mol/s

Moles of 1 vol.% CO Initial: 0.00003407 mol/s ×10^{-2} = 3.407 × 10^{-7} mol/s

Moles of CO Converted: 3.407 × 10^{-7} mol/s × 0.4325 = 1.4735 ×10^{-7} mol/s

Catalyst Mass: 0.6 × 10^{-3} g

Activity: [1.4735 x 10^{-7} mol/s] / [0.6 × 10^{-3} g] = 24.56 × 10^{-5} mol s^{-1} g^{-1}

5.3. Specific surface area calculations

Table S10. Summarized specific surface area of $LaCoO_3$ perovskite NPs (femtosecond laser-produced and chemically synthesized)

Sample	Size [nm]	SSA [m^2 g^{-1}]
5.8 J cm^{-2}	27	30.48
5.1 J cm^{-2}	25	32.92
4.5 J cm^{-2}	21	39.19
Reference	45	18.29

Note: specific surface area (SSA) = Total surface area/catalyst mass = 6/ρ·d

Table S11. Summary of nominal-TOF calculations at different temperatures, assuming specific (110) planes of $LaCoO_3$ perovskite NPs produced at femtosecond laser fluences of 4.5, 5.1, and 5.8 J cm^{-2}, and a reference catalyst.

T [°C]	TOF [s^{-1}] 4.5 J cm^{-2}	TOF [s^{-1}] 5.1 J cm^{-2}	TOF [s^{-1}] 5.8 J cm^{-2}	TOF [s^{-1}] *Reference*
175	-	-	0.04	0.10
200	-	0.03	0.09	0.29
250	-	0.07	0.25	0.89
300	0.04	0.16	0.46	2.19
350	0.10	0.30	0.72	2.45
400	0.20	0.48	0.99	1.24

Table S12. Summarized selectivity at different temperatures for CO-PROX reaction on $LaCoO_3$ perovskite NPs femtosecond laser-produced at fluences of 4.5, 5.1, and 5.8 J cm^{-2}, and a reference catalyst.

T [° C]	[S_{CO2} %] 4.5 J cm^{-2}	[S_{CO2} %] 5.1 J cm^{-2}	[S_{CO2} %] 5.8 J cm^{-2}	[S_{CO2} %] *Reference*
175	100	100	100	100
200	100	100	100	100
250	98	100	100	100
300	71	100	100	100
350	50	99	99	79
400	28	83	89	29

5.4. Nominal-TOF calculation

This example illustrates the TOF calculation for one specific sample and temperature. All other calculations were performed analogously.

Crystallographic parameters

Reference code:	04-006-2093
Crystal system:	Hexagonal
Space group:	R3c
Space group number:	167
a (Å):	5.42
b (Å):	5.42
c (Å):	13.11
Alpha (°):	90
Beta (°):	90
Gamma (°):	120

Density of $LaCoO_3$ = 7.29 g/cm^3

Catalyst mass = 0.6 mg

Number of Co atom per unit cell: 6

Area for (110) plane = c × a × sqrt(3) = 1.23 × 10^{-18} m^2

Gas Constant (R): 8.314 J/mol K

Avogadro's Number: 6.022×10^{23} mol^{-1}

Standard pressure : 101325 Pascal (equivalent to 1 atm)

Moles of surface Co per gram of catalyst (for sample F: 5.8 J cm^{-2}, at 400 °C):

[Number of Co atoms per plane × SSA (m^2/g)] / [Area (110) plane (m^2) × Avogadro's Number (mol^{-1})]

Moles of surface Co per gram = 2.469 × 10^{-4} mol/g

Total moles of active sites = 2.469 × 10^{-4} mol/g × 0.6 × 10^{-3} g (catalyst mass) = 1.4815 × 10^{-7} mol

Total molar flow rate = [P × V/R × T] = 3.407 × 10^{-5} mol/s

Moles of 1 vol.% CO Initial: 3.407 × 10^{-5} mol/s × 10^{-2} = 3.407 × 10^{-7} mol/s

Molar flow rate of converted CO = 3.407 × 10^{-7} mol/s × 43.25 × 0.01 = 1.4735 × 10^{-7} mol/s

TOF = Molar rate for converted CO / total moles of active sites

TOF = 1.4735 × 10^{-7} mol/s / 1.4815 × 10^{-7} mol = 0.994 s^{-1}

5.5. Analysis of reference catalyst

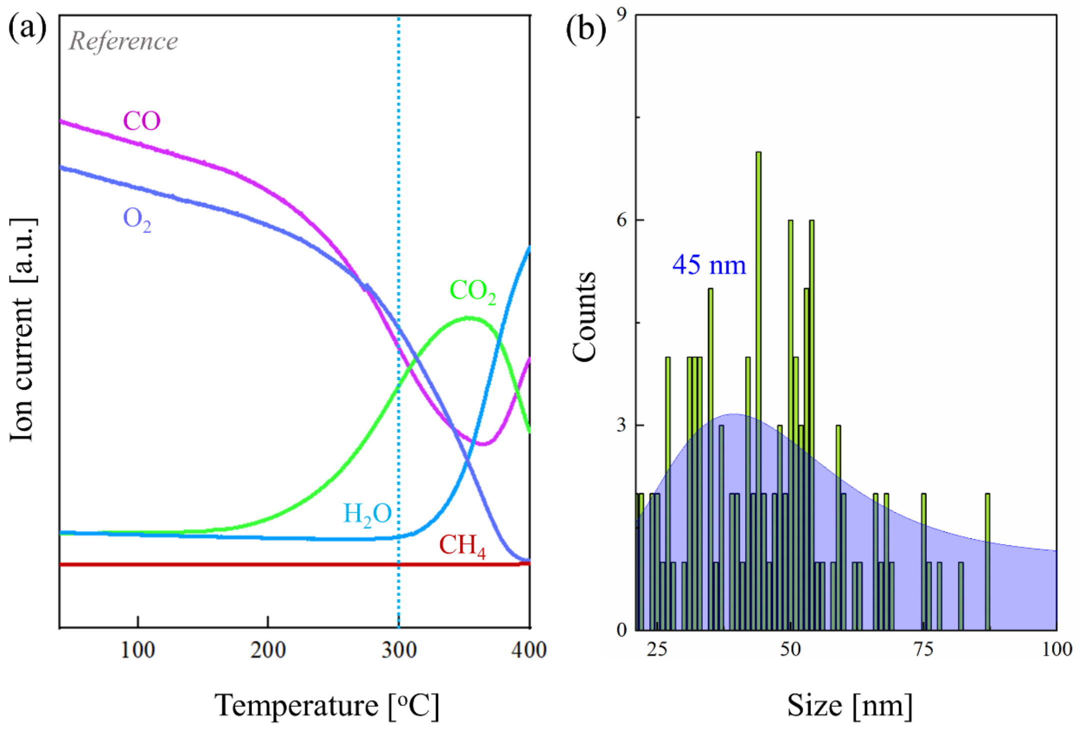

Figure S33. Analysis of (chemically-synthesized) reference sample NPs (sol-gel method)[8] : (a) MS data for perovskite NPs indicating RWGS above 350 °C; (b) Size distribution analysis. Dashed lines indicate the onset of water formation.

6. Electron microscopy of the reference catalyst

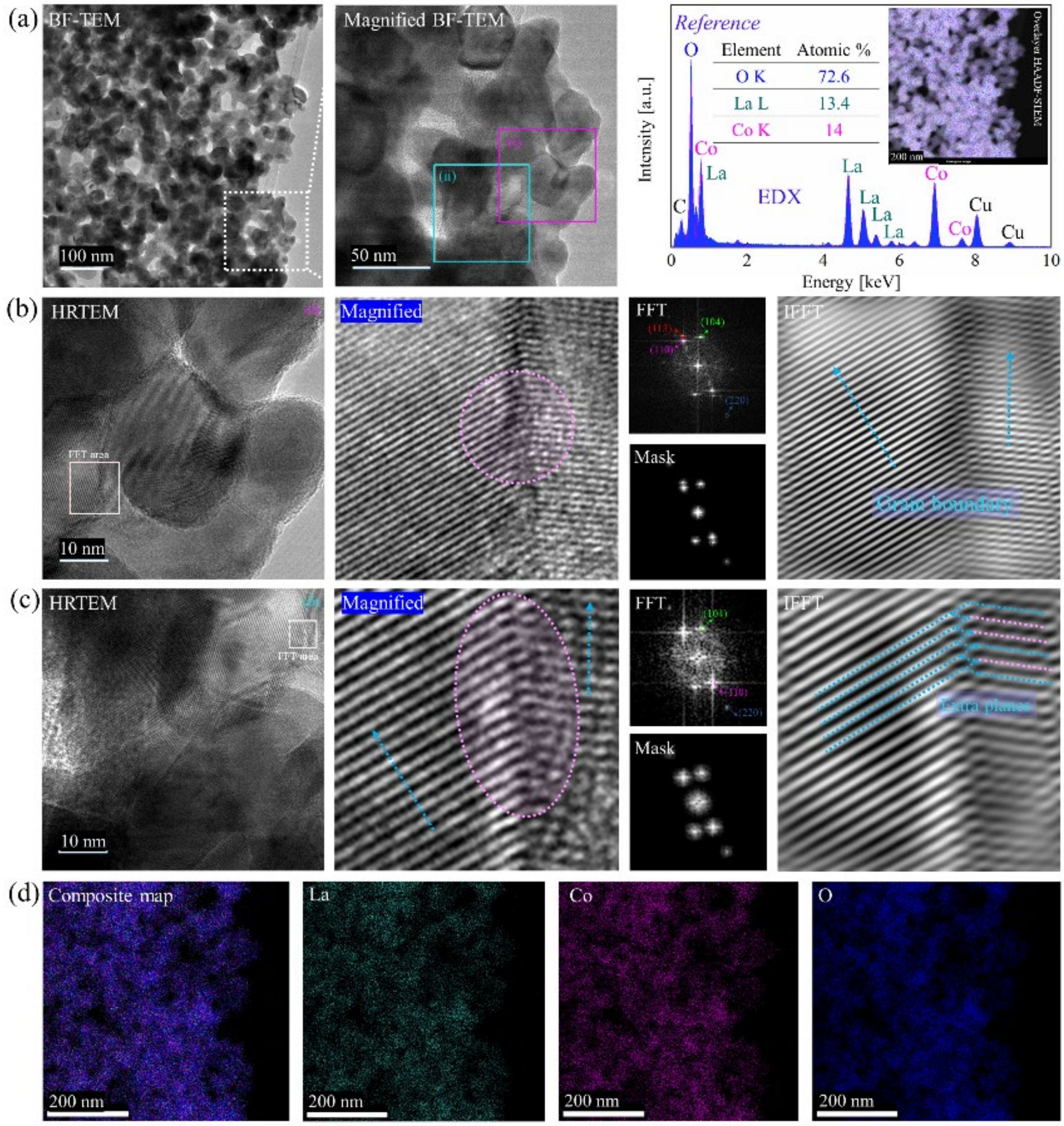

Figure S34. Electron microscopy and EDX mapping analysis of chemically synthesized $LaCoO_3$ perovskite nanoparticles. (a) BF-TEM, magnified BF-TEM and EDX spectrum obtained from BF-TEM area with overlayer HAADF-STEM (inset); (b) HRTEM (i), magnified area, FFT/mask and IFFT and displays grain boundary. An EDX spectrum is also provided; (c) HRTEM (ii), magnified area, FFT/mask and its FFT shows extra planes; (d) Composite EDX map, along with individual elemental maps for La, Co and O.

Table S13. Phase and reference lattice distances of $LaCoO_3$ perovskite NPs based on SAED pattern from ICDD database.

Indexed	04-006-2093	SAED ring No	h	k	l	d [nm]
Material	$LaCoO_3$	(1)	0	1	2	0.382
Mineral name	Rhombohedral	(2)	1	1	0	0.271
Space group number	165	(3)	1	0	4	0.268
		(4)	1	1	3	0.230
		(5)	0	2	4	0.191
		(6)	1	1	6	0.170
		(7)	3	0	0	0.156
		(8)	1	2	5	0.147

7. Stability test and post-reaction electron microscopy analysis of PLAL $LaCoO_3$

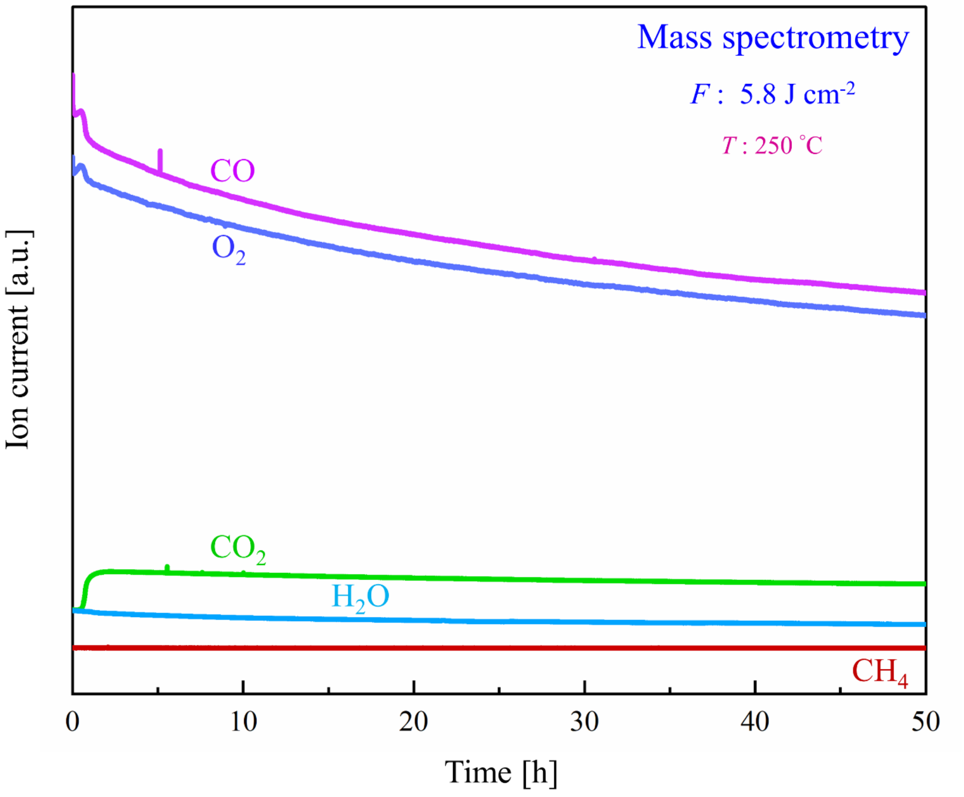

Figure S35. MS data for the stability test of $LaCoO_3$ perovskite nanoparticles femtosecond laser-produced (*F*: 5.8 J cm^{-2}) at 250 °C over a period of 2 days.

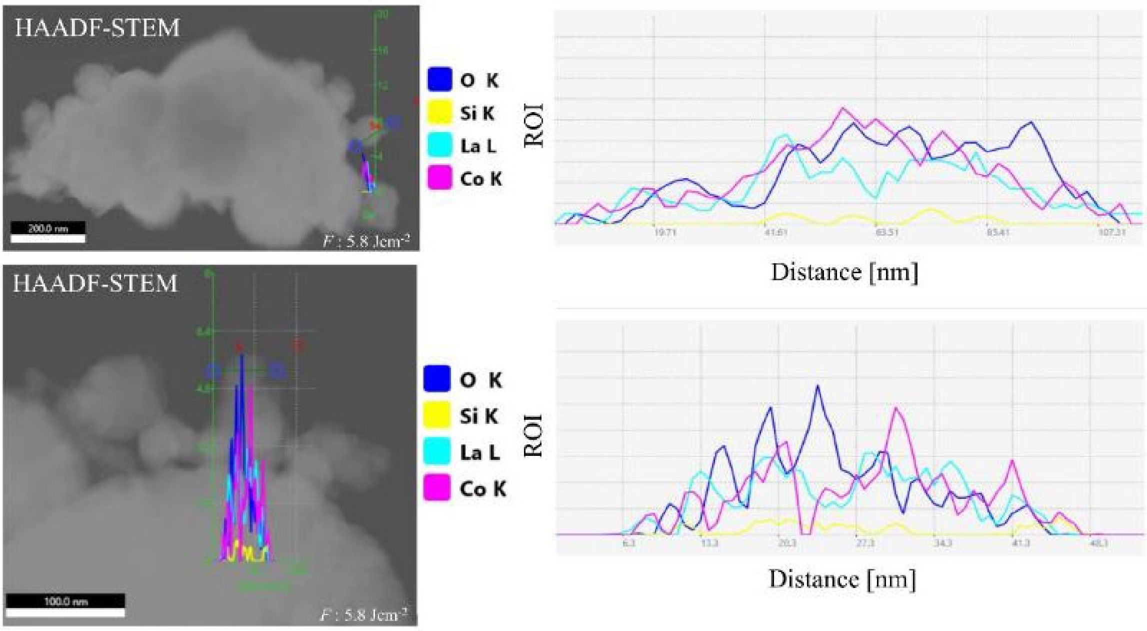

Figure S36. Post-reaction, HAADF-STEM images and corresponding line scan profiles of $LaCoO_3$ perovskite nanoparticles femtosecond laser-produced (*F*: 5.8 J cm^{-2}).

8. Supplementary references

[1] Jee, Y.; Becker, M. F.; Walser, R. M. Laser-induced damage on single-crystal metal surfaces. *Journal of the Optical Society of America B* **1988,** 5 (3), 648.

[2] Döring, S.; Richter, S.; Nolte, S.; Tünnermann, A. In situ imaging of hole shape evolution in ultrashort pulse laser drilling. *Optics Express* **2010,** 18 (19), 20395.

[3] Doñate-Buendía, C.; Fernández-Alonso, M.; Lancis, J.; Mínguez-Vega, G. Overcoming the barrier of nanoparticle production by femtosecond laser ablation in liquids using simultaneous spatial and temporal focusing. *Photonics Research* **2019,** 7 (11), 1249.

[4] Lasemi, N.; Rentenberger, C.; Pospichal, R.; Cherevan, A. S.; Pfaffeneder-Kmen, M.; Liedl, G.; Eder, D. Femtosecond laser-assisted synthesis of Ni/Au BONs in various alcoholic solvents. *Applied Physics A* **2019,** 125 (8), 544.

[5] Lasemi, N.; Rentenberger, C.; Liedl, G.; Eder, D. The influence of the fluid nature on femtosecond laser ablation properties of a SiO_2/Si target and synthesis of ultrafine-grained Si nanoparticles. *Nanoscale Advances* **2020,** 2 (9), 3991.

[6] Lasemi, N.; Wicht, T.; Bernardi, J.; Liedl, G.; Rupprechter, G. Defect-Rich CuZn Nanoparticles for Model Catalysis Produced by Femtosecond Laser Ablation. *ACS Applied Materials & Interfaces* **2024,** 16 (29), 38163.

[7] Lasemi, N.; Liedl, G.; Rupprechter, G. Formation of Periodic Surface Structures by Multipulse Femtosecond Laser Processing of Au-Coated Ni in Various Fluids. *ACS Applied Engineering Materials* **2023,** 1 (4), 1263.

[8] Yigit, N.; Föttinger, K.; Bernardi, J.; Rupprechter, G. Preferential CO oxidation (PROX) on $LaCoO_3$–based catalysts: Effect of cobalt oxidation state on selectivity. *Journal of Catalysis* **2025,** 443, 115973.
